# Reactive Oxygen Species Localization Programs Inflammation to Clear Microbes of Different Size

**DOI:** 10.1016/j.immuni.2017.02.013

**Published:** 2017-03-21

**Authors:** Annika Warnatsch, Theodora-Dorita Tsourouktsoglou, Nora Branzk, Qian Wang, Susanna Reincke, Susanne Herbst, Maximiliano Gutierrez, Venizelos Papayannopoulos

**Affiliations:** 1 Antimicrobial Defence Laboratory, The Francis Crick Institute, 1 Midland Rd, London NW1 1AT, UK; 2 Host-Pathogen Interactions in Tuberculosis Laboratory, The Francis Crick Institute, 1 Midland Rd, London NW1 1AT, UK

**Keywords:** neutrophil, reactive oxygen species, inflammation, microbe size, interleukin-1β, swarm, cluster, cellular stoichiometry, *Candida albicans*, fungal infection

## Abstract

How the number of immune cells recruited to sites of infection is determined and adjusted to differences in the cellular stoichiometry between host and pathogen is unknown. Here, we have uncovered a role for reactive oxygen species (ROS) as sensors of microbe size. By sensing the differential localization of ROS generated in response to microbes of different size, neutrophils tuned their interleukin (IL)-1β expression via the selective oxidation of NF-κB, in order to implement distinct inflammatory programs. Small microbes triggered ROS intracellularly, suppressing IL-1β expression to limit neutrophil recruitment as each phagocyte eliminated numerous pathogens. In contrast, large microbes triggered ROS extracellularly, amplifying IL-1β expression to recruit numerous neutrophils forming cooperative clusters. Defects in ROS-mediated microbe size sensing resulted in large neutrophil infiltrates and clusters in response to small microbes that contribute to inflammatory disease. These findings highlight the impact of ROS localization on signal transduction.

## Highlights

• The clearance of microbes of different size involves distinct inflammatory programs
• ROS adjusts neutrophil recruitment to the stoichiometry of host-pathogen interactions
• ROS localization acts as a sensor of microbe size to tune IL-1-β-driven inflammation
• IL-1β amplifies neutrophil recruitment and clustering to clear large microbes

## Graphical Abstract

**Figure:**
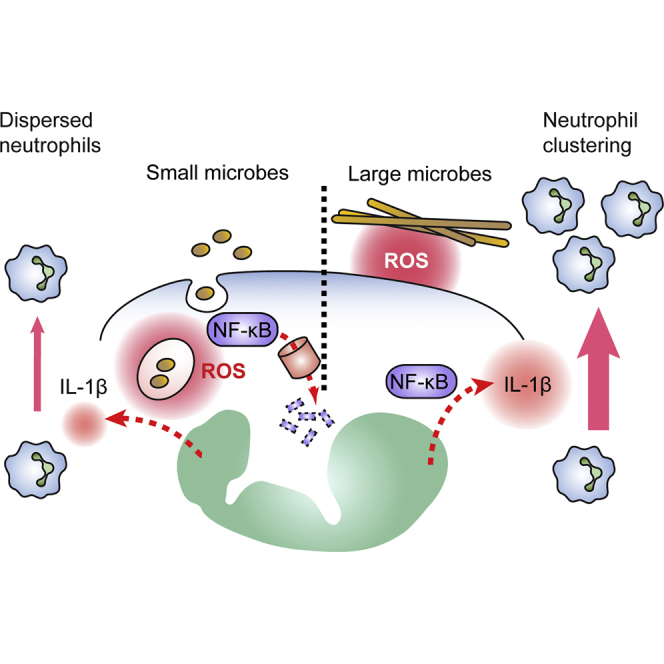

## Teaser

Inflammation recruits neutrophils to fight invading pathogens of different size. Warnatsch et al. show that reactive oxygen species localization tunes inflammation to compensate for differences in the number of neutrophils required to clear microbes of different size.

## Introduction

Neutrophil recruitment is a central aspect of the inflammatory process and is critical for clearing a variety of pathogens. The number of neutrophils at sites of infection must be tightly regulated to ensure that sufficient neutrophils are recruited for efficient clearance while minimizing excess recruitment that drives immune pathology (Medzhitov, 2008). The mechanisms that define the optimum number of neutrophils at sites of inflammation are unknown.

Microbe size plays a critical role in pathogen virulence as with the large invasive filamentous hyphae of *Candida albicans* and *Aspergillus fumigatus* (Brown et al., 2012). Conceivably, microbe size could greatly influence the stoichiometry of host-pathogen interactions. Furthermore, neutrophils may have to act cooperatively to combat large microbes. Yet, whether the clearance of microbes of different size involves different numbers of neutrophils and cooperative strategies is unknown.

Neutrophil recruitment is regulated by pro-inflammatory cytokines such as interleukin-1β (IL-1β) (Amulic et al., 2012). Microbe sensing activates NF-κB to transcribe IL-1β, which inflammasomes process into a mature form (Latz et al., 2013, Netea et al., 2008, Plato et al., 2015). In turn, IL-1β upregulates interleukin-17 (IL-17) and chemokines such as CXCL1 and CXCL2 to recruit neutrophils (Miller et al., 2006, Miller et al., 2007, Park et al., 2005). Fungal hyphae activate the inflammasome more potently than yeast in isolated macrophages but the in vivo relevance of this disproportionate response has not been investigated (Joly et al., 2009, Saïd-Sadier et al., 2010). Furthermore, differential cytokine expression in macrophages and dendritic cells has been attributed to the selective activation of innate immune receptors (Gantner et al., 2005, Kashem et al., 2015, van der Graaf et al., 2005).

Recent work has implicated neutrophils in IL-1β production upon bacterial infection (Chen et al., 2014, Cho et al., 2012, Karmakar et al., 2015), suggesting that neutrophils could play more central roles in modulating inflammation. In comparison to other phagocytes, neutrophils generate higher concentrations of ROS (Devalon et al., 1987, Nathan and Shiloh, 2000, Silva, 2010). In addition to their cytotoxic role, ROS regulate cell signaling (Murphy et al., 2011) and inhibit inflammasome activation and cytokine expression (Bagaitkar et al., 2015, Han et al., 2013, Harbort et al., 2015, Huang et al., 2015, Meissner et al., 2008, Meissner et al., 2010, Morgenstern et al., 1997). These observations are consistent with hyper-inflammatory pathology in chronic granulomatous disease (CGD) caused by mutations in the NOX2 NADPH oxidase (Rieber et al., 2012). However, the physiological purpose of this oxidative regulatory mechanism is still unknown. Using microbes with varying sizes, we have demonstrated that the ability to sense the differential localization of ROS allows neutrophils to adjust inflammation by modulating their own recruitment and cooperation to effectively clear microbes of different size.

## Results

### Microbe Size Regulates Neutrophil Recruitment and Clustering

**Figure 1:**
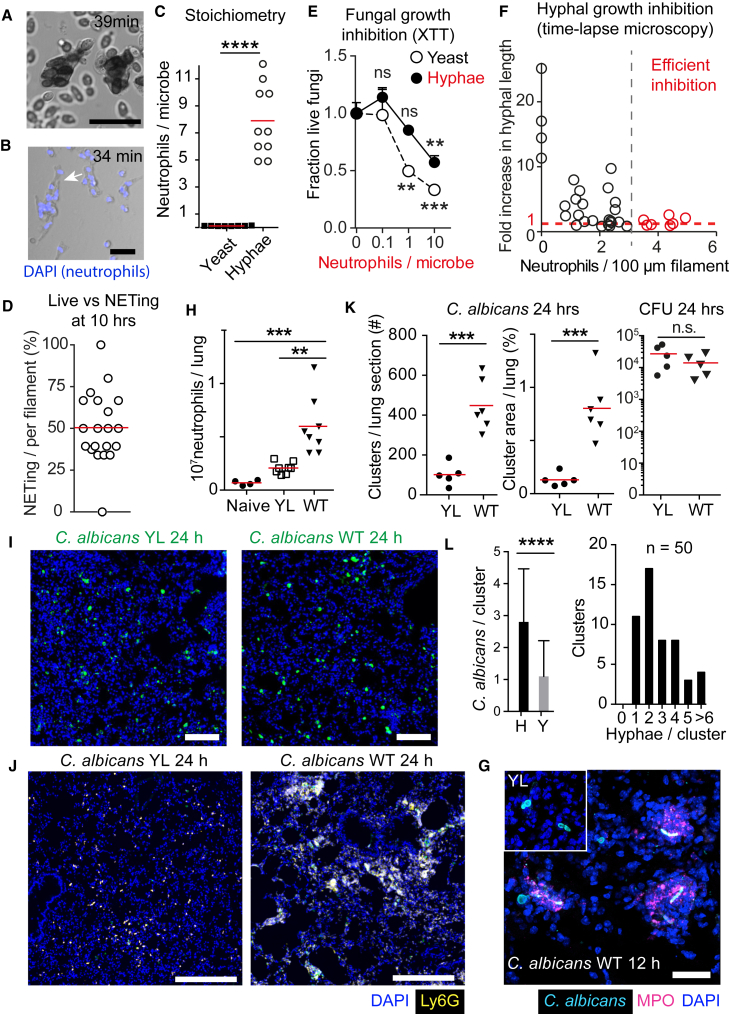
Microbe Size Regulates Inflammation (A and B) Time-lapse microscopy of neutrophils during infection with *C. albicans* yeast (MOI 40) (A) and hyphae (B). Extracellular DNA stained with Sytox Green (A and B) and total DNA with DAPI (B) (blue). Scale bars represent 10 μm (A) and 100 μm (B). Representative of three (A) and five (B) experiments. (C) Ratio of neutrophils interacting with *C. albicans* yeast or hyphae. Statistics by two-tailed Student’s t test. (D) Percent of total neutrophils attached to fungal filaments that released NETs. (E) *C. albicans* growth inhibition by neutrophils compared to fungus alone monitored by an enzymatic XTT assay at different neutrophil to microbe ratios. Data are means ± SD of technical duplicates. Representative of three independent experiments. Statistics by two-tailed Student’s t test comparing changes over baseline (*C. albicans* alone). (F) Fold increase in hyphal length during a 10 hr incubation (at 12 hr) plotted against the number of attached neutrophils at 2 hr, analyzed by time-lapse microscopy. (G) Immunofluorescence confocal micrographs depicting YL and WT *C. albicans* hyphae (cyan) and MPO-positive neutrophils (magenta) in thick lung sections 12 hr after infection. Scale bar represents 30 μm. (H) Number of neutrophils in lungs of naive WT mice or 24 hr after infection with yeast-locked (YL) or WT *C. albicans*, measured by FACS, gated on live CD45^+^, CD11b^+^, and Ly6G^+^. Each point represents one animal. Statistics by one-way ANOVA and Tukey’s multiple comparison post-test. Please also see Figure S1A. (I) WT or yeast-locked (YL) *C. albicans* (green) distribution in lungs after 24 hr. Scale bars represent 100 μm. (J) Immunofluorescence confocal micrographs depicting dispersed and clustering neutrophils in lung sections of mice infected with yeast-locked or WT *C. albicans*, 24 hr after infection stained for DNA (DAPI, blue), neutrophils (Ly6G, yellow), and *C. albicans* (green). Scale bars represent 250 μm. Please also see Figure S1B. (K) Number of neutrophil clusters, average cluster area per lung section, and *C. albicans* colony forming units (cfu) 24 hr after infection with YL or WT *C. albicans*. Each point represents one animal. Statistics by two-tailed Student’s t test. (L) Number of yeast (Y) and hyphae (H) in 50 randomly selected neutrophil clusters in mice infected with WT *C. albicans* (left) and the distribution of clusters with respect to the number of hyphae per cluster (right). Data are means ± SD. Statistics by two-tailed Student’s t test. ^∗∗∗∗^p < 0.0001, ^∗∗∗^p < 0.001, ^∗∗^p < 0.01.

To examine the impact of microbe size on neutrophil responses, we took advantage of the fungal pathogen *C. albicans*, which grows as small yeast or as large filamentous hyphae. First, we investigated how microbe size impacts the stoichiometry of the interaction between neutrophils and *C. albicans* yeast and hyphae by time-lapse video microscopy (Figures 1A and 1B). On average, neutrophils ingested 8 yeast particles (Figure 1C), whereas each 100-μm-long hyphal filament was engaged by 8 neutrophils exhibiting a 100-fold difference in stoichiometry (Figure 1C). Half of the neutrophils interacting with hyphae released neutrophil extracellular traps (NETs). The rest of the population remained alive for at least 10 hr (Figure 1D). NETs control hyphae (Bianchi et al., 2011, Branzk et al., 2014) but we suspected that long-lived neutrophils might play a regulatory role.

To determine whether the clearance of yeast and hyphae required different numbers of neutrophils, we monitored their impact on the viability of a yeast-locked (YL) *C. albicans* mutant that is unable to form hyphae (Δ*hgc1*) or pre-formed WT hyphae. Neutrophils restricted yeast viability at a 1:1 ratio. In contrast, a 10:1 ratio of neutrophils to hyphae was required to control hyphae (Figure 1E). To define the number of neutrophils required to control hyphae more accurately, we examined filament growth suppression by time-lapse microscopy. At least 3 neutrophils per 100 μm filament were needed for efficient growth suppression (Figure 1F). Therefore, few neutrophils were required to clear small yeast and a higher number to control large hyphae.

Next, we examined whether neutrophil recruitment in vivo depended on microbe size. In the lung, WT fungi switch to hyphae but the YL mutant remained in the yeast form (Figure 1G). Neutrophil recruitment occurred within 24 hr of infection, whereas fungal clearance took place 24–48 hr after infection. Infection with WT *C. albicans* yielded a 3-fold higher number of infiltrating neutrophils compared to the YL *Δhgc1* strain (Figures 1H and S1A). While both strains dispersed homogeneously at 24 hr (Figure 1I), the lungs of mice infected with WT *C. albicans* contained neutrophils organized in large clusters (Figures 1J, 1K, and S1B) predominately around hyphae (Figures 1G and 1L). In contrast, the lungs of mice infected with the YL strain contained significantly fewer neutrophil clusters, with neutrophils being more evenly dispersed, despite bearing a comparable fungal load at 24 hr after infection (Figure 1K). These data pointed to an unknown mechanism that adjusts neutrophil recruitment and organization to the distinct requirements associated with microbes of different size.

### IL-1β Regulates Neutrophil Recruitment and Clustering

**Figure 2:**
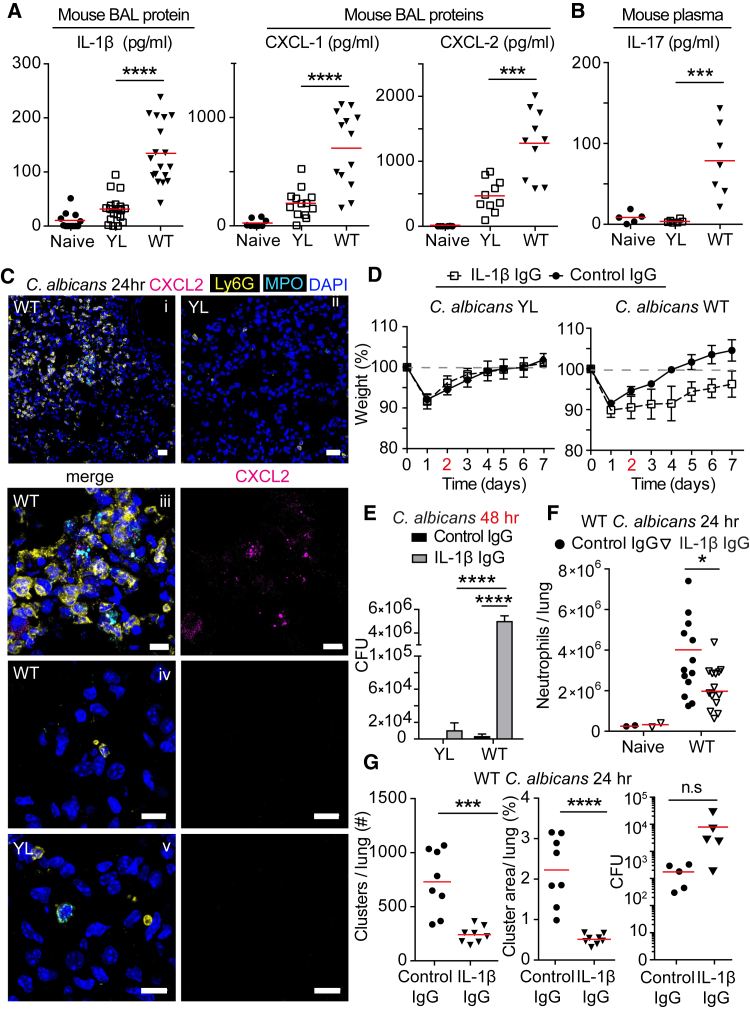
IL-1β Is Critical for Clustering to Control Hyphae (A and B) Cytokine analysis in WT mice after intratracheal infection with 10^5^ yeast-locked (YL) or WT *C. albicans*. IL-1β, CXCL-1, and CXCL-2 protein in the BAL 24 hr after infection (A). IL-17 protein in plasma 48 hr after infection (B). Each point represents one animal. Statistics by one-way ANOVA followed by Tukey’s multiple comparison post-test. Please also see Figures S2A and S2B. (C) Immunofluorescence confocal micrographs of lung sections from WT mice infected with 10^5^ yeast-locked (YL; ii, zoomed image in v) or WT *C. albicans* (i, zoomed image in iii, zoomed image from area with dispersed neutrophils in iv) after 24 hr, stained for DNA (DAPI, blue), neutrophils (Ly6G, yellow), and CXCL2 (magenta) and MPO (cyan). Scale bars in (i) and (ii) represent 20 μm; in (iii)–(v) 10 μm. (D–G) WT mice treated with isotype control or anti-IL-1β neutralizing antibody after intratracheal infection of 10^5^ yeast-locked (YL) or WT *C. albicans* 24 hr after infection. Please also see Figure S2C. (D) Mean weight monitoring for seven animals per group. Representative of two experiments. Data are means ± SD. (E) *C. albicans* colony forming units (cfu) in the lung 48 hr after infection. Mean ± SD of six animals per group. Statistics by two-way ANOVA followed by Sidak’s multiple comparison post-test. (F) Number of neutrophils per lung analyzed by FACS. Each point represents one animal. Statistics by two-way ANOVA followed by Sidak’s multiple comparison post-test. (G) Number of neutrophil clusters, average cluster area per lung section, and fungal burden. Each point represents one animal. Please also see Figure S3A. Statistics by two-tailed Student’s t test. ^∗∗∗∗^p < 0.0001, ^∗∗∗^p < 0.001, ^∗^p < 0.05.

To understand how neutrophil clustering is regulated, we first examined whether microbe size regulated cytokine responses. YL *C. albicans* infection triggered reduced IL-1β, IL-6, CXCL-1, and CXCL-2 in the bronchoalveolar lavage (BAL) after 24 hr (Figures 2A, S2A, and S2B) and IL-17 in circulation after 48 hr (Figure 2B). Consistently, CXCL-2 expression concentrated around neutrophil clusters only in mice infected with WT *C. albicans* (Figure 2C). Then, we investigated whether the clearance of yeast and hyphae had different IL-1β requirements using a blocking antibody that depleted IL-1β efficiently in vivo (Figure S2C). IL-1β blockade had a minor effect on the clearance of the YL strain (Figure 2E) as both isotype and anti-IL-1β-treated mice made a full recovery (Figure 2D). In contrast, WT *C. albicans* infection induced persistent weight loss and increased pulmonary fungal load at 48 hr, upon IL-1β suppression (Figures 2D and 2E). Consistently, IL-1β blockade reduced neutrophil recruitment (Figure 2F) and clustering (Figures 2G and S3A) in response to WT *C. albicans* or *A. fumigatus* infection (Figures S3B–S3D) despite the trend for an increase in the fungal load already by 24 hr (Figure 2G, right). Therefore, IL-1β signaling was critical for amplifying neutrophil recruitment and clustering to clear hyphae but was dispensable against yeast.

### Neutrophils Are Required for Selective IL-1β Induction

**Figure 3:**
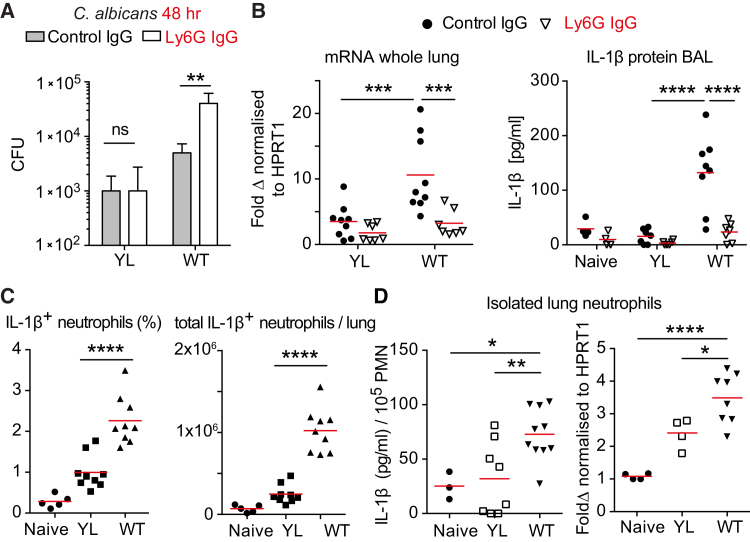
Neutrophils Produce IL-1β in a Microbe-Size-Dependent Manner (A and B) WT mice treated with isotype control IgG or anti-Ly6G antibody prior to intratracheal infection with 10^5^ yeast-locked (YL) or WT *C. albicans*. Please also see Figure S4A. (A) *C. albicans* burden in the lung 48 hr after infection. Mean ± SD of three animals per group. Statistics by two-way ANOVA followed by Sidak’s multiple comparison post-test. (B) IL-1β mRNA in the lung normalized to HPRT1 expression and IL-1β protein expression in bronchoalveolar lavage 24 hr after infection. Each point represents one animal. Statistics by two-way ANOVA followed by Sidak’s multiple comparison post-test. (C) Fraction and total number of lung neutrophils positive for intracellular IL-1β staining measured by FACS. Neutrophils were gated as live CD45^+^, CD11b^+^, Ly6G^+^ cells. Each point represents one animal. Statistics by one-way ANOVA followed by Tukey’s multiple comparison post-test. Please also see Figure S4C. (D) IL-1β protein and mRNA in neutrophils isolated by negative selection from lungs of WT mice infected with 10^5^*C. albicans* YL or WT. Each point represents one animal. Statistics by one-way ANOVA followed by Tukey’s multiple comparison post-test. ^∗∗∗∗^p < 0.0001, ^∗∗∗^p < 0.001, ^∗∗^p < 0.01, ^∗^p < 0.05.

To confirm that IL-1β controlled hyphae by regulating neutrophils, we tested whether depleting neutrophils would also differentially affect the clearance of the two fungal forms. Neutrophil depletion (Figure S4A) did not affect the YL yeast clearance (Figure 3A) but resulted in higher fungal load upon infection with WT *C. albicans* hyphae 48 hr after infection. To examine whether neutrophils contributed to IL-1β production, we measured IL-1β upon neutrophil depletion. Neutrophil depletion abrogated IL-1β protein release and mRNA expression and attenuated CXCL-1 and CXCL-2 in response to *C. albicans* infection (Figures 3B and S4B). Confirming that neutrophils produce IL-1β in a microbe-size-dependent manner in vivo, we detected a higher number of IL-1β-positive neutrophils in the lungs of mice infected with WT *C. albicans* than mice infected with the YL strain, in the absence of restimulation (Figures 3C and S4C). Similarly, affinity-purified neutrophils from mice infected with WT *C. albicans* contained higher concentrations of IL-1β mRNA and protein than neutrophils purified from mice infected with the yeast-locked strain (Figure 3D). These data identified neutrophils as important sources of IL-1β in fungal infection, adjusting cytokine production according to the size of the microbe they encountered.

### ROS Localization Regulates IL-1β-Driven Neutrophil Clustering

**Figure 4:**
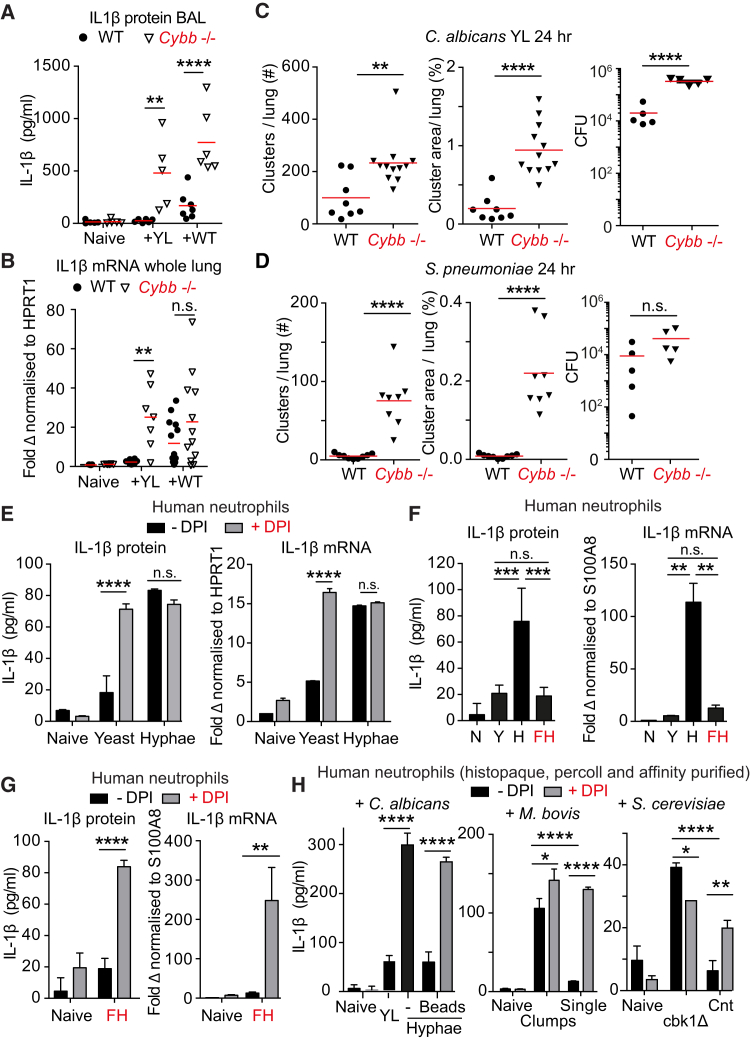
ROS Regulates Inflammation in a Microbe-Size-Dependent Manner (A and B) IL-1β protein in the BAL (A) and mRNA in whole lung (B) of WT and *Cybb*^−/−^ mice, 24 hr after infection with 10^5^ yeast-locked (YL) or WT *C. albicans*. Each point represents one animal. Statistics by two-way ANOVA followed by Sidak’s multiple comparison post-test. (C and D) Number of neutrophil clusters, average cluster size per lung section, and microbe burden 24 hr after infection in WT and *Cybb*^−/−^ mice infected with (C) 10^5^ yeast-locked (YL) *C. albicans* or (D) 10^4^*S. pneumonia*. Each point represents one animal. Statistics by two-tailed Student’s t test. Please also see Figures S5C and S5E. (E) IL-1β protein released after 4 hr (left) and IL-1β transcript after 1 hr (right) from purified human neutrophils untreated or treated with DPI and yeast or preformed hyphae. (F) IL-1β protein after 4 hr and transcript after 1 hr from naive purified human neutrophils or treated with yeast (Y), hyphae (H), or fragmented hyphae (FH). (G) IL-1β protein after 4 hr and transcript after 1 hr in naive purified human neutrophils untreated or treated with DPI or fragmented hyphae (FH). (H) IL-1β protein released by neutrophils purified over Histopaque and Percoll as well as an affinity-based negative selection kit (99.2% pure, please see Figure S6A). Naive neutrophils or activated with *C. albicans* yeast or hyphae in the presence of 4 μm opsonized polystyrene-coated beads (left), with single bacteria or large aggregates of *M. bovis* BCG (middle), or with aggregates of *cbk1Δ S. cerevisiae* or single cells of the control strain (right) in the absence or presence of DPI. Total IL-1β protein was measured in response to *S. cerevisiae*. (E–H) Stimulation at MOI 10. Data are means ± SD of technical duplicates. Representative of three independent experiments. Statistics by two-way ANOVA followed by Sidak’s multiple comparison post-test. ^∗∗∗∗^p < 0.0001, ^∗∗∗^p < 0.001, ^∗∗^p < 0.01, ^∗^p < 0.05.

Next, we explored how microbe size tunes cytokine expression. Neutrophil-derived NETs regulate IL-1β expression during sterile inflammation (Warnatsch et al., 2015). NETs are selectively induced by hyphae (Branzk et al., 2014) and could amplify IL-1β induction in a microbe-size-specific manner. To test this hypothesis, we employed mice deficient in the ROS-producing enzyme myeloperoxidase (MPO) that is required for NET formation (Branzk et al., 2014, Kolaczkowska et al., 2015, Metzler et al., 2011, Metzler et al., 2014, Papayannopoulos et al., 2010). MPO deficiency led to elevated pulmonary IL-1β expression in response to WT *C. albicans* (Figure S5A) indicating that during infection, neutrophils upregulate IL-1β production in an alternative ROS-dependent manner. Consistently, infection with YL *C. albicans* strains yielded elevated IL-1β protein in *Cybb*-deficient mice, which lack the gp91phox subunit of the NADPH oxidase and do not produce superoxide (Figure 4A; Pollock et al., 1995), and a 9-fold upregulation of IL-1β transcript compared to only a 2-fold non-significant increase in response to WT *C. albicans* (Figure 4B) likely due to a minor yeast population (Figure 1L). Therefore, ROS suppressed cytokine transcription in a microbe-size-dependent manner in vivo.

Since elevated IL-1β expression drove neutrophil clustering, we examined whether the lack of ROS would upregulate neutrophil clustering in response to YL *C. albicans*. Consistently, neutrophil numbers (Figure S5B) and clustering were significantly higher in *Cybb*-deficient animals infected with YL *C. albicans* (Figures 4C and S5C). The elevated fungal burden in *Cybb*-deficient mice is consistent with impaired phagocytosis and pathogen clearance due to the lack of ROS. In contrast there was no increase in clustering in *Cybb*-deficient mice upon infection with WT *C. albicans* (Figure S5D). Similarly, neutrophil clusters were nearly absent in the lungs of WT mice infected with the small bacterium *S. pneumonia* 24 hr after infection but potent clustering was elicited in *Cybb*-deficient animals (Figure 4D) along with elevated IL-1β, CXCL1, and CXCL2 expression without a significant increase in bacterial burden (Figures 4D, right, and S5E). Overall, we found no correlation between fungal burden and neutrophil clustering at 24 hr but consistent correlation with microbe size, IL-1β signaling, or the absence of ROS (Figure S5F). Moreover, blood circulating neutrophil numbers were identical in *Cybb*-deficient mice (Figure S5G). Together, these data indicate that ROS regulated neutrophil clustering by selectively suppressing cytokine expression in response to small microbes, rather than via the amplification of expression in response to large hyphae.

To investigate whether neutrophils were sufficient to generate IL-1β in a microbe-size-dependent manner, we incubated histopaque- and percoll-purified primary human neutrophils with YL yeast or WT pre-formed hyphae in vitro. Hyphae triggered potent IL-1β expression and protein release into cell culture supernatants within 4 hr of stimulation. By comparison, YL *C. albicans* triggered significantly lower concentrations of IL-1β (Figure 4E). Pretreatment of neutrophils with the NOX2 inhibitor diphenyleneiodonium (DPI) led to a substantial increase in IL-1β protein release upon infection with YL yeast to concentrations that were comparable to induction by WT hyphae. In contrast, DPI had no effect on cytokine release by neutrophils activated with hyphae. Similar results were obtained with murine bone marrow neutrophils isolated from WT or *Cybb*-deficient mice (Figure S5H). Since the differential cytokine expression was completely abrogated in the absence of ROS, these data pointed to the absence of mechanisms that depend on differences in microbe surface molecules.

To confirm the dependence of cytokine expression on microbe size rather than other factors such as difference in microbial surface molecules or released metabolites, we incubated human neutrophils with fragmented hyphae of sizes comparable to yeast. Fragmentation of hyphae disrupted their ability to potently upregulate mature IL-1β protein and transcript (Figure 4F). The ability of fragmented hyphae to induce IL-1β release was rescued by blocking NOX2 activity with DPI, indicating that hyphal fragments had not lost their ability to activate neutrophils but instead had suppressed cytokine expression in a ROS-dependent manner (Figure 4G). Moreover, using ultra-pure human neutrophils (Figure S6A), we found that phagocytosis of small 4 μm beads blocked cytokine expression in response to hyphae in a ROS-dependent manner (Figure 4H, left). Similarly, large *M. bovis* Bacillus Calmette-Guérin (BCG) aggregates, but not single bacteria, upregulated IL-1β and *Saccharomyces cerevisiae cbk1Δ* mutants, which fail to separate during cell division and form large multinucleated cells (Racki et al., 2000), selectively induced IL-1β expression compared to a control single-cell strain. Importantly, inhibiting the ROS burst with DPI restored IL-1β upregulation in response to small BCG and *S. cerevisiae* single cells (Figure 4H, middle and right). Furthermore, large β-glucan-coated beads induced higher cytokine expression than small beads, where inhibition of expression could be alleviated with DPI. This ROS-mediated suppression of cytokine expression depended on the phagocytosis of small particles because treatment with cytochalasin D, which blocks phagocytosis, upregulated mature IL-1β release in response to yeast (Figure S6B). These data uncover a phagocytosis-dependent oxidative mechanism that enables neutrophils to tune cytokine production to microbe size. Consistently, coinfection with YL fungi reduced cytokine expression (Figure S6C) and led to more diffuse neutrophil clustering in response to WT fungi in vivo (Figure S6D), confirming the ability of phagocytosis to suppress inflammation.

### Microbes of Different Size Induce ROS at Different Locations

**Figure 5:**
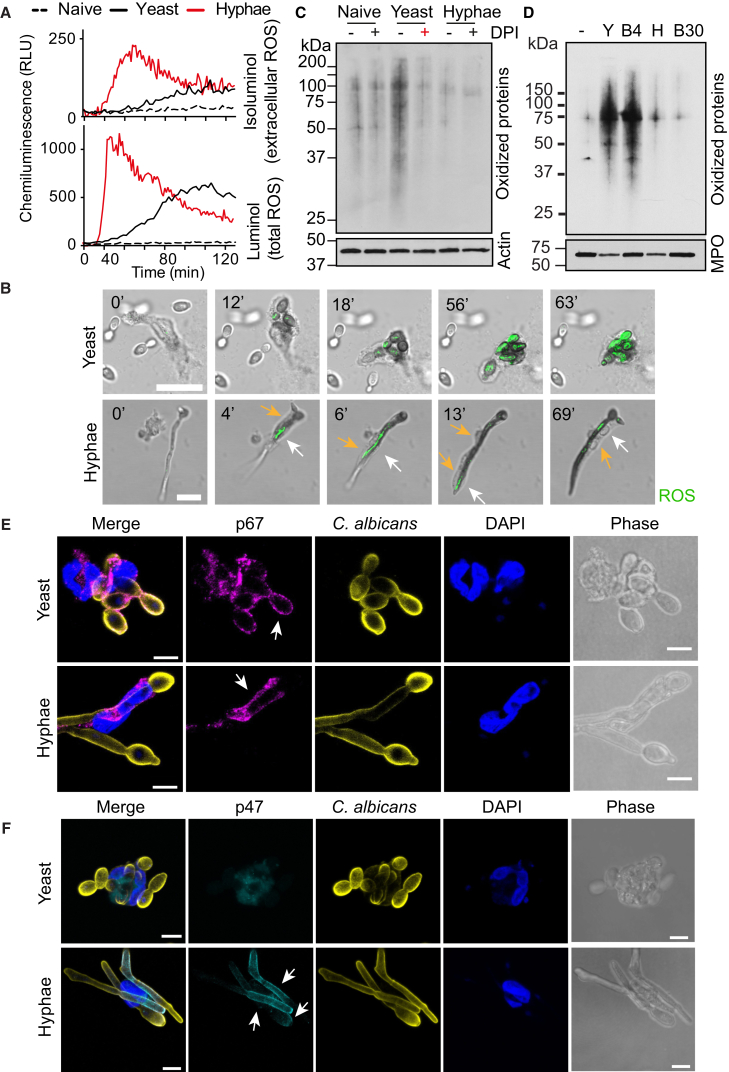
Differential ROS Localization Induces Selective Neutrophil Protein Oxidation in a Microbe-Size-Dependent Manner (A) ROS production by naive human blood-derived neutrophils or in the presence of YL yeast or WT preformed hyphae, detected by chemiluminescence of the cell-impermeable isoluminol to detect extracellular ROS (top) or by the cell-permeable luminol to detect both intracellular and extracellular ROS (bottom), in the presence of exogenous horseradish peroxidase to detect superoxide production. (B) Representative time-lapse microscopy depicting neutrophil ROS production in response to yeast or hyphae monitored by NBT staining. Dynamic ROS production in response to hyphae denoted by white arrows. Neutrophil cell body indicated by yellow arrows. Scale bars represent 10 μm. (C) Carbonylated proteins in lysates of human neutrophils 2 hr after incubation with yeast (YL) or hyphae untreated or treated with DPI and analyzed by western immunoblotting. Representative of five independent experiments. (D) Carbonylated proteins in lysates of human neutrophils treated with either yeast (Y), hyphae (H), 4 μm (B4), or 30 μm (B30) β-glucan-coated beads. (E and F) Phase contrast and immunofluorescence staining of human neutrophils interacting with yeast or hyphae stained for (E) p67-phox (magenta), *C. albicans* (yellow), DNA (DAPI, blue), or (F) p47-phox (cyan). Arrows indicate assembly of the NADPH oxidase components on membranes associated with *C. albicans*. Scale bars represent 5 μm.

Next, we explored whether there were fundamental differences in the ROS burst elicited by small and large microbes via two chemiluminescent probes: luminol, which is cell permeable and detects both intracellular and extracellular ROS, and isoluminol, which detects only extracellular ROS. Both yeast and hyphae elicited a potent ROS burst, albeit with somewhat different dynamics. However, only hyphae were able to induce strong extracellular ROS (Figure 5A). The initial delay in ROS detection in response to yeast during the reaction was consistent with ROS being consumed intracellularly. In contrast, in response to hyphae, ROS were released extracellularly where they could interact more efficiently with luminol. To confirm this hypothesis, we detected ROS by monitoring nitro blue tetrazolium (NBT) oxidation by microscopy. Neutrophils phagocytizing yeast exhibited slightly slower ROS dynamics but nevertheless yielded a powerful and persistent ROS burst within 20–60 min (Figure 5B). In contrast, neutrophils interacting with hyphae yielded faster but highly transient ROS bursts that relocated dynamically as the points of contact between neutrophil and filament shifted (Figure 5B, white arrows). Next, we investigated the impact of ROS localization on protein oxidation by analyzing protein carbonylation in neutrophil lysates. Yeast triggered potent protein oxidation, which was blocked by DPI and absent in neutrophils incubated with hyphae (Figure 5C). The vast majority of the total protein content was of neutrophil origin in these reactions (Figure S6E), suggesting that it is the neutrophil and not fungal proteins being oxidized. This hypothesis was validated by the strong protein oxidation of neutrophil proteins in cells treated with small 4 μm β-glucan-coated beads. Large 30 μm beads or hyphae did not trigger neutrophil protein oxidation (Figure 5D).

To uncover the basis for the differential ROS localization, we examined the localization of the NADPH oxidase. During neutrophil activation, cytoplasmic NOX2 oxidase subunits associate with membrane-bound subunits to assemble the active oxidase enzyme. p67phox colocalized both with the plasma membrane regions contacting hyphae and with phagosomal membranes surrounding yeast (Figure 5E). p47phox associates with the NADPH oxidase complex on the plasma membrane, but not the phagosome (Ueyama et al., 2007). As neutrophils stretched in an attempt to phagocytize hyphae, the NADPH oxidase complex assembled selectively on plasma membrane areas contacting hyphae as indicated by the co-localization of p47phox with the fungal cell wall staining (Figure 5F, arrows). In contrast, p47phox did not colocalize with phagocytized yeast. Hence, the differential assembly of the NADPH oxidase allowed yeast to trigger intracellular ROS driving potent cellular oxidation and hyphae to elicit ROS extracellularly, sparing neutrophil proteins from oxidation.

### ROS Localization Regulates NF-κB in a Microbe-Size-Dependent Manner

**Figure 6:**
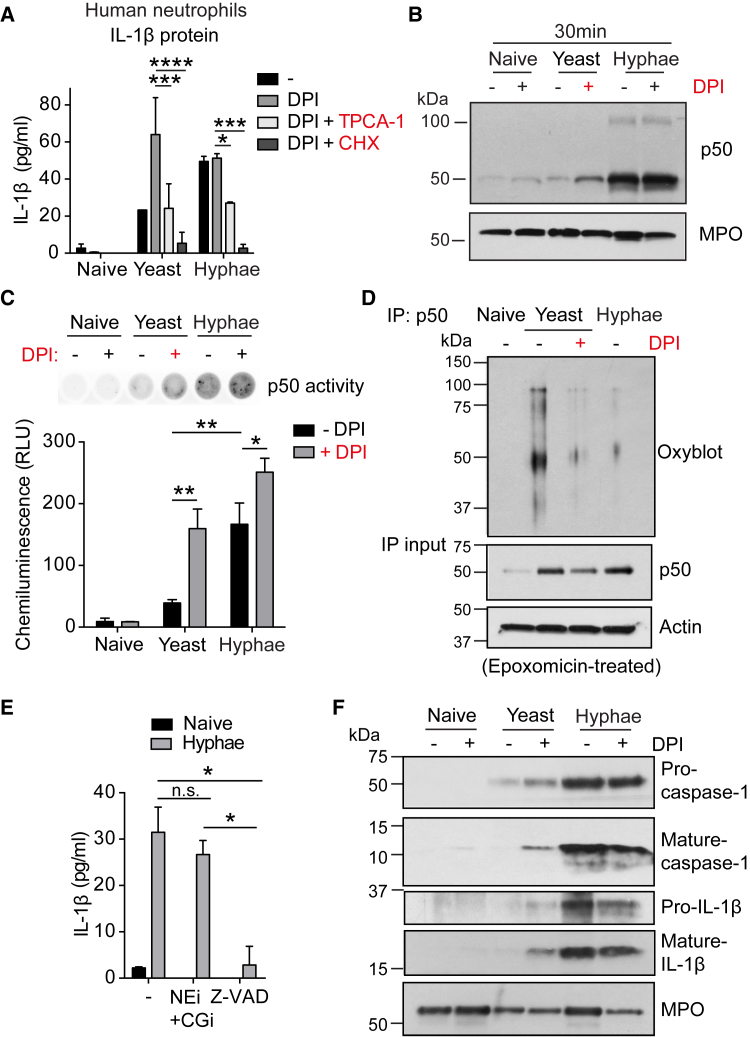
ROS Localization Regulates NF-κB in a Microbe-Size-Dependent Manner (A) IL-1β release in response to yeast or hyphae by human neutrophils treated with NOX2 inhibitor DPI or in combination with IKK1 and 2 inhibitor TPCA-1 or translation inhibitor cycloheximide (CHX). Data are means ± SD of technical duplicates. Representative of three independent experiments. (B) Immunoblot analysis of NF-κB p50 and myeloperoxidase (MPO) in human neutrophil lysates 30 min after exposure to yeast or hyphae. (C) p50 activity in human neutrophil lysates 45 min after *C. albicans* exposure in the presence or absence of DPI measured by p50 binding to its consensus DNA sequence in coated 96-well plates. Data are means ± SD of technical duplicates. Representative of three independent experiments. (D) Carbonylation of p50 immunoprecipitated from lysates of epoxomicin-treated human neutrophils 45 min after exposure to yeast or hyphae. Representative of three independent experiments. (E) Effect of protease inhibitors on IL-1β release. Human neutrophils untreated or treated with the neutrophil elastase inhibitor GW311616A (NEi), in combination with cathepsin G inhibitor (CGi) or the pan-caspase inhibitor Z-VAD-fmk and exposed to hyphae. Data are means ± SD of technical duplicates. Representative of three independent experiments. (F) Western immunoblot of caspase-1 and IL-1β maturation in lysates of human neutrophils after 1 hr of exposure to yeast or hyphae untreated or treated with DPI. MPO serves as loading control. Representative of two independent experiments. Statistics by two-way ANOVA followed by Sidak’s multiple comparison post-test. ^∗∗∗∗^p < 0.0001, ^∗∗∗^p < 0.001, ^∗∗^p < 0.01, ^∗^p < 0.05.

To investigate whether ROS regulated cytokines in a microbe-size-dependent manner at the transcriptional level, we incubated human neutrophils with the translation inhibitor cycloheximide (CHX) and stimulated them with yeast or hyphae. CHX efficiently blocked mature IL-1β protein release in response to hyphae as well as yeast upon NOX2 inhibition with DPI (Figure 6A). Furthermore, TPCA-1, an inhibitor of IKK1 and IKK2 kinases upstream of NF-κB, also suppressed mature IL-1β release in DPI-treated neutrophils in response to yeast (Figure 6A). These data suggest that ROS suppresses NF-κB activity selectively in response to yeast and hyphae. IL-1β is transcribed by a heterodimer composed of the NF-κB p50 and p65 subunits. Among all the potential targets for oxidation in the pathway, we focused on p50 because oxidation inhibits its transcriptional activity (Pineda-Molina et al., 2001, Toledano and Leonard, 1991) and would override any upstream regulatory events. Assuming ROS might regulate IL-1β release by interfering with NF-κB signaling directly, we analyzed p50 protein expression in neutrophil lysates 30 min after *C. albicans* exposure. In neutrophils treated with YL *C. albicans*, p50 was not upregulated. In contrast, p50 was strongly upregulated in response to hyphae (Figure 6B). Inhibiting the ROS burst with DPI was sufficient to partially restore p50 in yeast-infected neutrophils. To examine whether the partial restoration of p50 expression in DPI-treated neutrophils was sufficient to restore NF-κB transcriptional activity, we employed a transcription factor-binding assay that measures the binding of active p50 to its consensus sequence. In contrast to neutrophils incubated with hyphae, p50 activity was not upregulated in yeast-treated neutrophils (Figure 6C). Inhibition of ROS production was sufficient to restore p50 transcriptional activity in yeast-treated cells comparable to neutrophils treated with hyphae (Figure 6C). Hence, upon phagocytosis of small microbes, intracellular oxidation attenuates NF-κB activity.

To establish whether p50 was differentially oxidized and degraded in response to stimulation with different fungal forms, we pretreated neutrophils with the proteasome inhibitor epoxomicin prior to incubation with *C. albicans*, which was sufficient to prevent p50 degradation (Figure 6D, IP input). Subsequent to fungal exposure, we collected neutrophil lysates, immunoprecipitated p50, and analyzed its oxidation. Treatment with yeast resulted in potent p50 oxidation (Figure 6D) and ubiquitination (Figure S6F) that could be blocked with DPI and restored to concentrations that were comparable to neutrophils stimulated with hyphae. These data confirmed that yeast but not hyphae drove ROS-mediated p50 oxidation, targeting the protein for degradation. Thus, NF-κB oxidation and transcriptional activity depend on the differential localization of ROS.

Finally, we investigated which neutrophil enzymes process IL-1β into its mature p17 form in response to hyphae using pharmacological inhibitors. Inhibitors of neutrophil elastase (NE) and cathepsin G (CG) did not affect IL-1β release, but caspase inhibition with z-VAD-fmk completely abolished cytokine production (Figure 6E), confirming macrophage data showing that ROS regulate IL-1β production by suppressing caspase-1 (Meissner et al., 2008, Meissner et al., 2010). However, the effect of ROS on the inflammasome has been demonstrated with soluble agonists in macrophages that generate only a fraction of the ROS produced by neutrophils. Therefore, we investigated how the differential ROS localization generated by live fungi affected inflammasome activation in neutrophils. Pro-caspase-1 was cleaved into its active p10 subunit upon incubation with hyphae, accompanied by maturation of the p17 IL-1β subunit. Inflammasome activation was insensitive to ROS suppression as it remained unaffected by DPI in response to hyphae (Figure 6F). In contrast, yeast failed to induce caspase-1 activation and IL-1β maturation but ROS inhibition partially restored mature Caspase-1 p10 and IL-1β p17 to 5% and 36% of the concentrations induced by hyphae. Therefore, both NF-κB signaling and inflammasome activation are sensitive to ROS localization.

## Discussion

Our findings demonstrated that neutrophils were the major drivers of microbe-size-dependent IL-1β amplification during pulmonary infection but that alveolar macrophages are likely to recruit the first wave of neutrophils. The selective oxidative regulation of neutrophil-derived cytokines tuned inflammation to microbe size. Neutrophils ingested small microbes and produced ROS intracellularly by assembling the NADPH oxidase on the phagosome membrane. ROS permeated into the cytosol and oxidized p50, promoting its degradation and limiting IL-1β release to attenuate excessive neutrophil recruitment and minimize the risk for immune pathology. In response to large pathogens, the NADPH oxidase assembled on the plasma membrane, pumping ROS in the extracellular space, thus sparing p50 from oxidation. Consequently, active NF-κB amplified IL-1β expression to recruit additional neutrophils and organize them into clusters required to clear large pathogens. Similarly, phagocytosis regulates NET formation by sequestration of neutrophil elastase (Branzk et al., 2014). These findings place phagocytosis as a central regulator of microbe-size-dependent neutrophil responses via different effector mechanisms.

This model counters the hypothesis that “frustrated phagocytosis” augments phagocyte activation by allowing prolonged receptor signaling from the phagocyte cell surface. Instead, receptor signaling in response to microbes of different size is comparable in neutrophils (Branzk et al., 2014), but selective phagocytosis of small microbes suppresses cytokine transcription by generating ROS intracellularly. Furthermore, β-glucan purified from *C. albicans* hyphae cell walls is more potent than yeast-derived β-glucan (Lowman et al., 2014). Yet, β-glucan is buried under a mannan layer in hyphae which is likely to counter these intrinsic properties (Steele et al., 2005). Given that soluble microbial agonists are likely to generate ROS extracellularly as they are not phagocytized (Goodridge et al., 2011), experiments with these compounds might not take into account the effects of ROS localization. Employing ROS as a signaling intermediate enables the immune system to sense the size in a variety of microbes irrespectively of specific microbial surface attributes.

Neutrophil clustering has also been reported in response to bacterial skin infection with *S. aureus* (Abtin et al., 2014) and sterile focal skin injury (Lämmermann et al., 2013). While we also observed some degree of neutrophil clustering upon infection with small microbes, the response is dramatically upregulated against large pathogens. Important differences exist between pulmonary infection that spreads microbes across the lung and the skin models that generate a challenge at a single location. Bacterial aggregation may also play a role in these responses because coagulates and clumping factor A induce *S. aureus* agglutination in vivo (Malachowa et al., 2016, McAdow et al., 2011). In addition, one can’t exclude that skin tissue disruption by growing hyphae, which constitutes a major fungal virulence mechanism, contributes to neutrophil activation in vivo.

Due to the lower amplitude of the ROS burst, macrophages and dendritic cells might be more sensitive to differences in the activation of innate immune receptors. Consistently, experiments with *C. albicans* skin infections show that Langerhans cells (LCs) distinguish yeast from hyphae by detecting β-glucan on the surface of yeast via the c-type lectin receptor Dectin-1 (Kashem et al., 2015). Selective IL-6 expression in response to yeast drives adaptive T helper 17 (Th17) cell responses. However, we detected lower concentrations of IL-17 upon infection with the yeast-locked mutant, reflecting potential differences between innate lymphoid cell and γδ-T cell responses that depend on IL-1β and adaptive Th17 cell polarization. However, neutrophil-derived extracellular ROS might influence neighboring DCs (Huang et al., 2015).

Recent work in apoptosis-associated speck-like protein containing a carboxy-terminal CARD (ASC)-deficient mice suggested that IL-1β plays no role in neutrophil recruitment and clearance of *A. fumigatus*, implicating monocyte-derived IL-1α instead (Caffrey et al., 2015). The study employed 500-fold higher infectious doses, which is likely to mask the requirement for neutrophil organization by saturating the lung with neutrophils as a result of an overwhelming fungal burden.

The sensitivity to the location of ROS may regulate many transcripts. ROS suppresses cytokine production by activating ataxia-telangiectasia mutated (ATM) (Harbort et al., 2015) to inhibit the p38 MAP kinase, which triggers the alternative NF-κB pathway. Patients with mutations in ATM suffer from chronic inflammation and it will be interesting to investigate whether they fail to respond selectively to microbe size. Moreover, neutrophils produce IL-17 upon *A. fumigatus* infection. IL-17 expression is unaltered in NOX2-deficient neutrophils, which might be consistent with RORγt being insensitive to ROS (Taylor et al., 2014).

Defending against microbes of different size relies on distinct innate inflammatory programs that may be relevant in helminth infection (Sutherland et al., 2014). This model explains the inflammatory pathology associated with CGD and potentially other conditions. Without ROS to detect microbe size, the innate immune system of CGD patients responds to every infection by erroneously mounting a hyper-inflammatory program designed to clear large pathogens.

## Experimental Procedures

### Mice

All experiments were conducted with age-matched, 8- to 12-week-old male or female C57BL/6J, gp91phox (*Cybb*)-deficient (B6.129S6-*Cybb*^*tm1Din*^/J), and MPO-deficient (B6.129X1-*Mpo*^*tm1Lus*^/J) mice, according to local guidelines and UK Home Office regulations under the Animals Scientific Procedures Act 1986 (ASPA).

### Microbes

Wild-type *C. albicans* (clinical isolate SC5314) and yeast-locked *C. albicans Δhgc1* (Zheng et al., 2004) were cultured overnight at 37°C in yeast extract peptone dextrose (YEPD) medium. YL *C. albicans* was subcultured in YEPD medium. WT *C. albicans* was subcultured for 4 hr either in RPMI medium to induce hyphal growth or in YEPD medium for in vivo infection experiments. Subcultures were centrifuged and resuspended in phosphate-buffered saline (PBS) immediately prior to infection. Fragmented hyphae approximately 5 μm long were generated by heat inactivation for 30 min at 90°C and shearing in an EmulsiFlex-C5 high-pressure homogenizer (Avestin). *S. pneumoniae* TIGR4 (Serotype 2) was cultured in brain-heart infusion broth at 37°C overnight, subcultured to an optical density of 0.4, and resuspended in PBS. *S. cerevisiae cbk1*Δ mutant (T74) (Racki et al., 2000) and control strains (PT31-52A) were cultured overnight at 30°C in YEPD medium. *M. bovis* BCG single cells were grown at 37°C, with shaking at 100 rpm, to an absorbance of 0.8 at 600 nm in Middlebrook 7H9 medium supplemented with 10% oleic acid, albumin, dextrose, and catalase (OADC supplement), 0.05% Tween-80, 0.4% glycerol, and 50 μg/mL hygromycin. Bacterial cultures were centrifuged and supernatants were repeatedly passed through a syringe for the removal of large aggregates. Large aggregates were grown without shaking for 3 days and centrifuged through a 50% Percoll bed containing 1× PBS to separate from single bacteria. Wild-type *A. fumigatus* (clinical isolate 13073) was grown on sabourad dextrose agar plates. When plates were fully covered, conidia were harvested by adding 0.1% Tween-20 in PBS and scraping them off. To obtain swollen conidia, *A. fumigatus* was subcultured for 4 hr at 37°C in RPMI medium. Swollen conidia were washed, counted, and resuspended in PBS for in vivo infection experiments.

### Pulmonary Infection Studies

To study the effects of blocking antibodies on mouse weight and fungal load at 48 hr after infection, mice were infected intratracheally with 1 × 10^4^ *C. albicans*. For analysis of cytokines in BAL, expression in lung homogenates, and immunofluorescence microscopy-based neutrophil clustering, 24 hr after infection, 1 × 10^5^ *C. albicans*, 1 × 10^5^ *S. pneumonia*, or 1 × 10^6^ *A. fumigatus* were employed. To analyze the effect of mixed *C. albicans* infection, a suspension of 5 × 10^4^ wild-type and 5 × 10^4^ yeast-locked *C. albicans* in PBS were administered intratracheally.

### Cell Culture

Peripheral blood was collected from healthy adult volunteers according to protocols approved by the ethics board of the Francis Crick Institute. Human neutrophils were freshly isolated over Histopaque 1119 gradient followed by a discontinuous Percoll gradient (Aga et al., 2002) if indicated followed by negative selection with the EasySep human neutrophil enrichment kit (Stemcell). Mouse neutrophils were isolated from *C. albicans*-infected lung cell suspensions or from bone marrow using the EasySep mouse neutrophil enrichment kit (Stemcell).

Mouse and human neutrophils were plated in Hank’s balanced-salt solution plus Ca^2+^ and Mg^2+^ supplemented with 3% human plasma or 10% fetal calf serum for mouse neutrophils, respectively. Cells were pretreated for 1 hr with 10 μM NADPH oxidase inhibitor diphenyleneiodonium (DPI, Sigma), 10 μM Neutrophil elastase inhibitor NEi (GW311616A, Sigma), 5 μM Cathepsin G inhibitor CGi (Millipore), 20 μM pan-caspase inhibitor (Z-VAD-fmk, Calbiochem), 5 μM IKK2 inhibitor 2-[(Aminocarbonyl)amino]-5-(4-fluorophenyl)-3-thiophenecarboxamide (TPCA-1, Biovision), 50 μg/mL translation inhibitor cycloheximide (CHX, Sigma), or 200 nM proteasome inhibitor Epoxomicin (Calbiochem). Neutrophils were stimulated with *C. albicans* yeast-locked, preformed hyphae or fragmented hyphae, as well as *S. cerevisiae* or *M. bovis* at a MOI 10 unless otherwise stated. For bead inhibition studies, neutrophils were pre-incubated with 4 μm polystyrene-coated flow cytometry beads (Kisker Biotech) that were opsonized for 1 hr in 100% human plasma and washed 3× in PBS, 30 min prior to stimulation with *C. albicans* preformed hyphae. For stimulation with β-glucan-coated beads, 4 μm (Kisker Biotech) and 30 μm (Sigma) polystyrene-based beads were incubated for 1 hr with 2 mg/mL β-glucan in PBS/EDTA and subsequently washed 3× in PBS/EDTA to remove soluble β-glucan.

## Author Contributions

A.W. designed and performed experiments, analyzed data, and wrote the manuscript with V.P., who designed and supervised the project. N.B. and T.-D.T. performed live imaging and fungal growth inhibition assays. S.H. and M.G. prepared BCG samples. T.-D.T., Q.W., and S.R. contributed to experiments.

## Supplemental Information

Document S1. Figures S1–S6, Table S1, and Supplemental Experimental Procedures

Document S2. Article plus Supplemental Information
