# Supplementary material for: Reactive Oxygen Species Localization Programs Inflammation to Clear Microbes of Different Size
Source: Immunity. 2017 Mar 21;46(3):421–32. doi: 10.1016/j.immuni.2017.02.013 (PMC5965455; doi:10.1016/j.immuni.2017.02.013)
Supplement: Document S1. Figures S1–S6, Table S1, and Supplemental Experimental Procedures [file mmc1.pdf]

Immunity, Volume *46*

Supplemental Information

Reactive Oxygen Species Localization Programs Inflammation to Clear Microbes of Different Size

Annika Warnatsch, Theodora-Dorita Tsourouktsoglou, Nora Branzk, Qian Wang, Susanna Reincke, Susanne Herbst, Maximiliano Gutierrez, and Venizelos Papayannopoulos

Figure S1

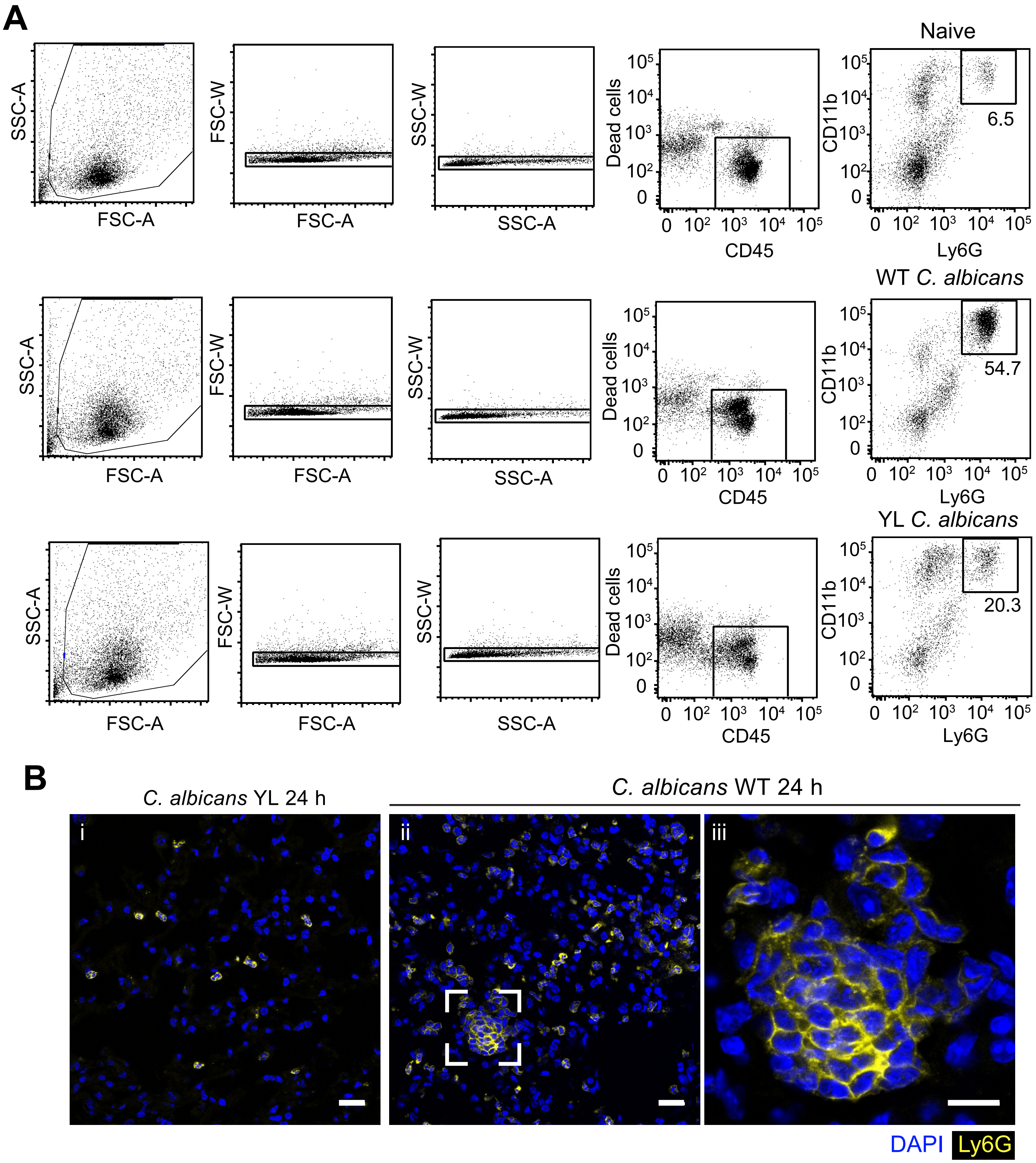

Figure S1, related to Figure 1H. Gating strategy for neutrophil count in infected lungs.

(A) FACS gating strategy for counting neutrophils in whole lungs of mice infected with yeast-locked (YL) or WT *C. albicans*. Neutrophils were gated as live, CD45+, CD11b+, Ly6G+ cells.
(B) Dispersed and clustered neutrophils 24 hrs post infection with YL or WT *C. albicans*. Lung sections stained for Ly6G (yellow), and DAPI (blue). Scale bars: 20μm (i and ii), 10μm (iii).

Figure S2

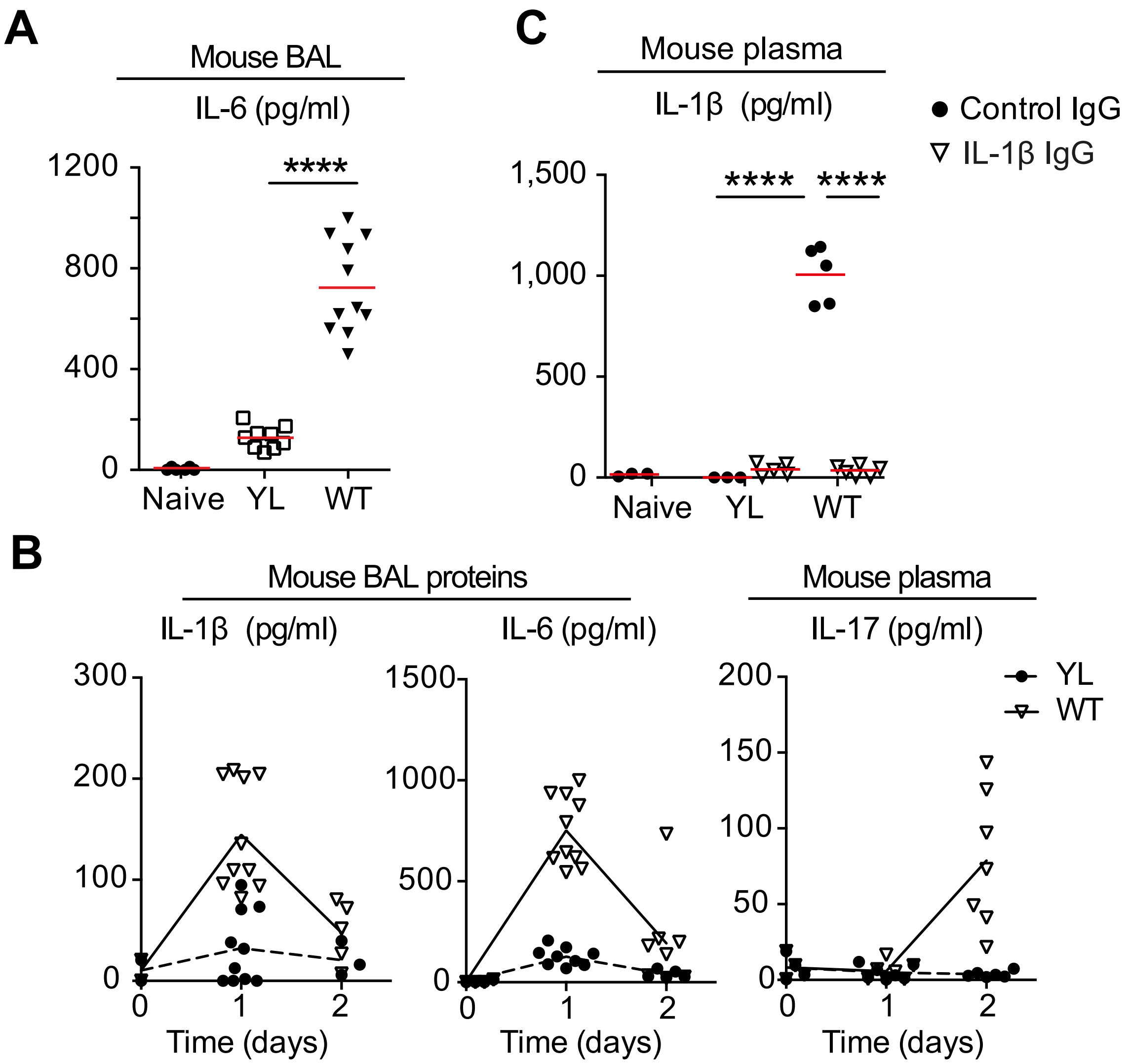

Figure S2, related to Figure 2. Neutrophil clustering and cytokine analysis in mice infected with *C. albicans*.

(A) Differential induction of IL-6 in the BAL of mice infected with 10^5 yeast-locked (YL) or WT *C. albicans*, 24 hrs post-infection. Each point represents one animal. Statistics by 2-way ANOVA and Tukey's post-test. **** $p < 0.0001$.
(B) IL-1β and IL-6 protein in the BAL and IL-17 protein in the plasma of mice infected with 10^5 yeast-locked (YL) or WT *C. albicans* 1 and 2 days post infection. Each point represents one animal.
(C) Upon pulmonary infection with yeast-locked (YL) or WT *C. albicans*, mice treated with an anti-IL-1β antibody do not bear significant amounts of circulating IL-1β in their blood plasma 24 hrs post-infection as compared to isotype control treated mice. Cytokine protein was detected by ELISA. Each point represents one animal. Statistics by 2-way ANOVA and Tukey's post-test. **** $p < 0.0001$.

Figure S3

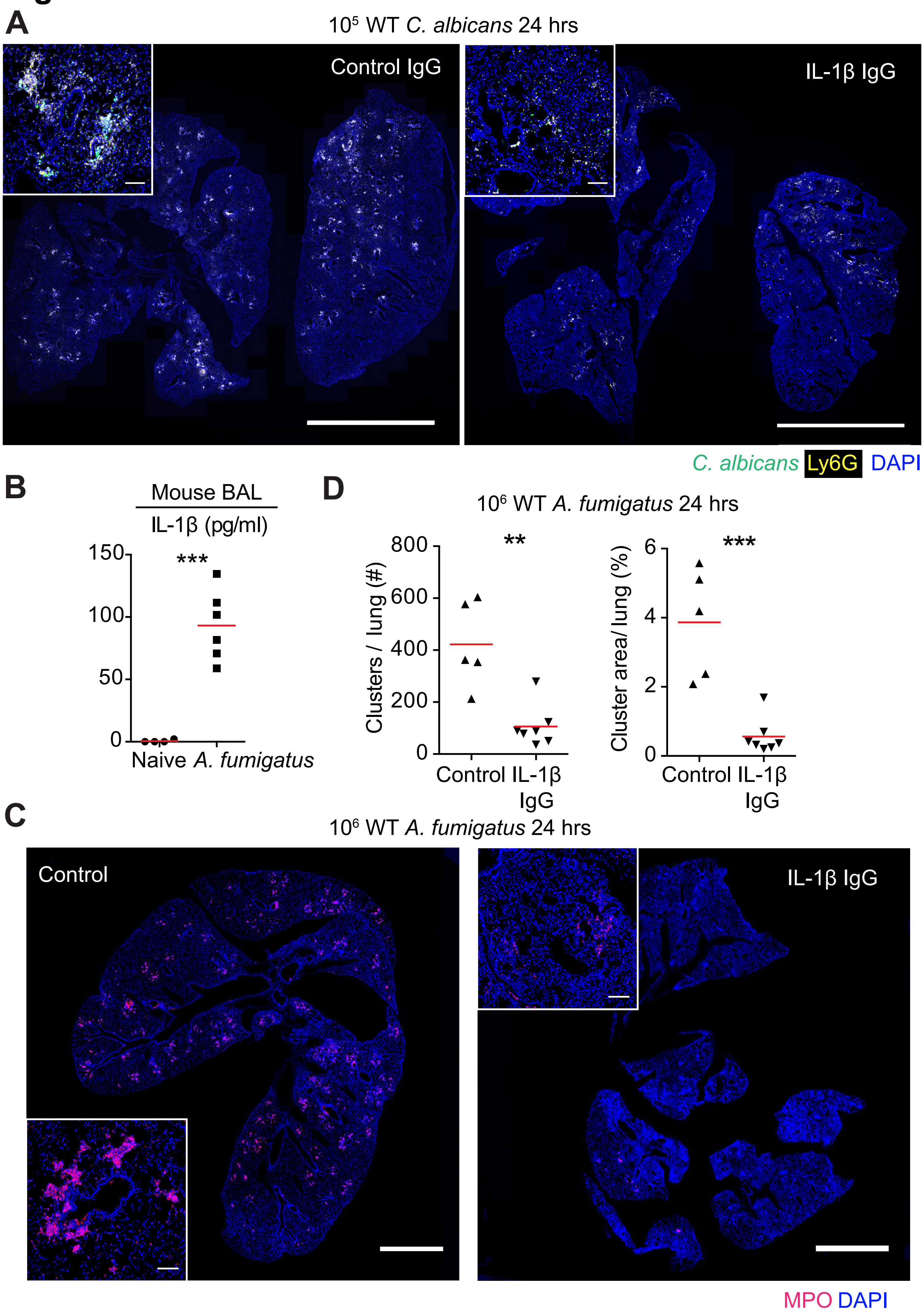
A
10⁵ WT C. albicans 24 hrs
Control IgG
IL-1β IgG
C. albicans Ly6G DAPI
B
Mouse BAL
IL-1β (pg/ml)
150
100
50
0
***
Naive A. fumigatus
D
10⁶ WT A. fumigatus 24 hrs
Clusters / lung (#)
800
600
400
200
0
**
Control IL-1β IgG
Cluster area/ lung (%)
6
4
2
0
***
Control IL-1β IgG
C
10⁶ WT A. fumigatus 24 hrs
Control
IL-1β IgG
MPO DAPI

Figure S3, related to Figure 2. Neutrophil responses to *C. albicans* and *A. fumigatus* pulmonary infection.

(A) Immunofluorescence confocal micrographs of lung sections stained for DNA (DAPI, blue), neutrophils (Ly6G, yellow) and *C. albicans* (green) from WT mice treated with an isotype control or an antibody blocking IL-1β, 24 hrs post infection and infected with 10^5 WT *C. albicans* yeast, which switches to hyphal growth *in vivo*. Scale bars: (A) 5 mm, (B) 2 mm, (a and b) inserts 100 μm.
(B) IL-1β production in the BAL of WT mice infected with 10^6 *A. fumigatus* swollen conidia 24 hrs post-infection measured by ELISA. Each point represents one animal. Statistics by two-tailed student's t-test.
(C) As in (A) but infected with 10^6 swollen *A. fumigatus* conidia, stained for DNA (DAPI, blue), neutrophils (MPO, red). Scale bars: 2 mm, inserts 100 μm.
(D) Number of neutrophil clusters and average cluster size per lung section in (C) for mice infected with 10^6 swollen *A. fumigatus* conidia. Each point represents one animal. Statistics by two-tailed student's t-test. *** $p < 0.001$, ** $p < 0.01$.

Figure S4

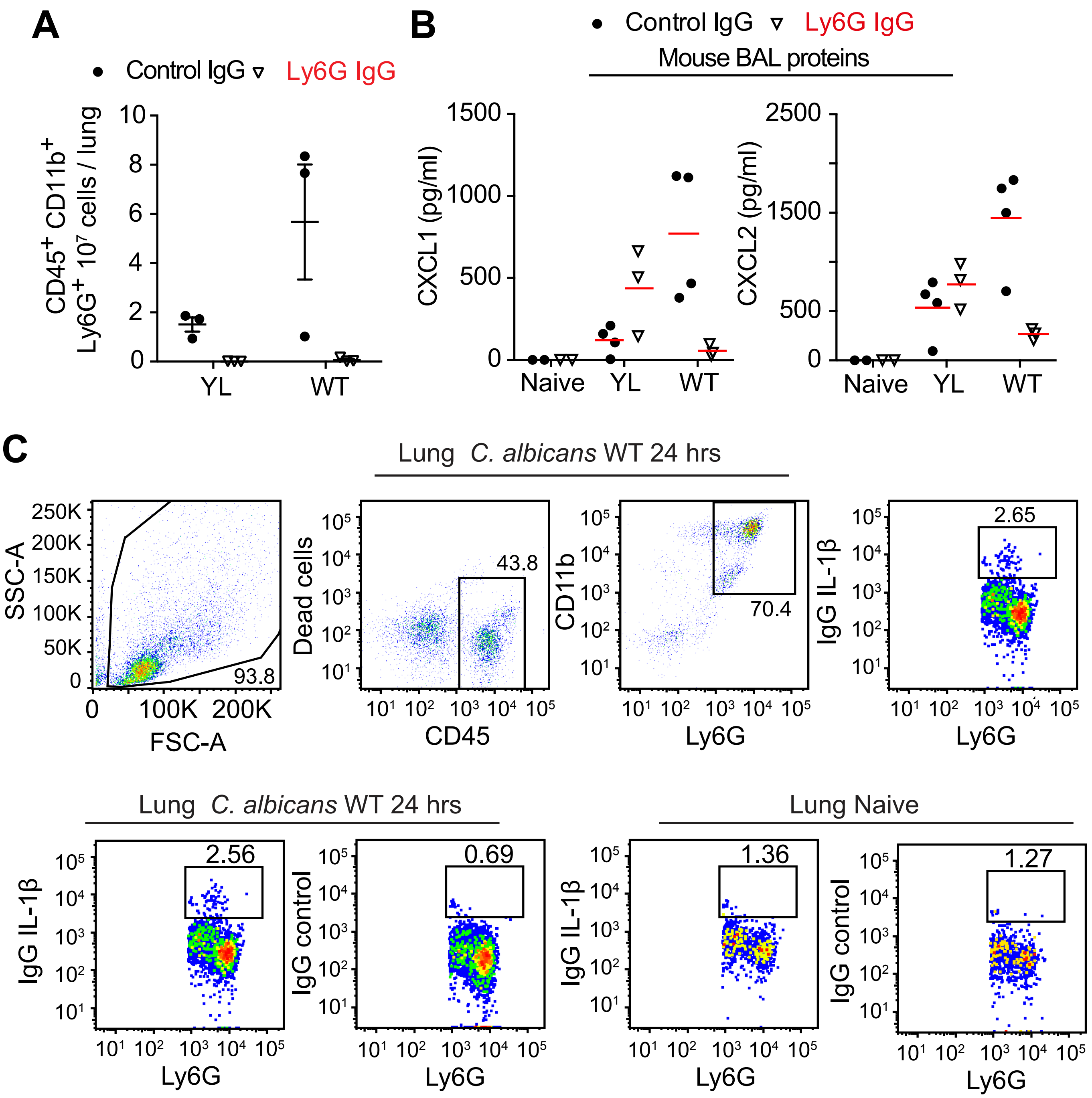

Figure S4, related to Figure 3. Effect of neutrophil depletion on pulmonary neutrophils and neutrophil chemokines.

(A) Effectiveness of neutrophil depletion in lungs of mice infected with yeast-locked (YL) or WT *C. albicans* and pretreated with anti-Ly6G antibody or an isotype control 24 hrs post-infection. Each point represents one animal.
(B) Effect of neutrophil depletion on the neutrophil chemokines CXCL1 and CXCL2 24 hrs post-infection, in the BAL of WT mice infected with yeast-locked (YL) or WT *C. albicans* and pre-treated with anti-Ly6G antibody or an isotype control. Each point represents one animal.
(C) FACS gating strategy for counting IL-1β+ lung neutrophils purified from the lungs of mice either uninfected or infected with WT *C. albicans*.

Figure S5

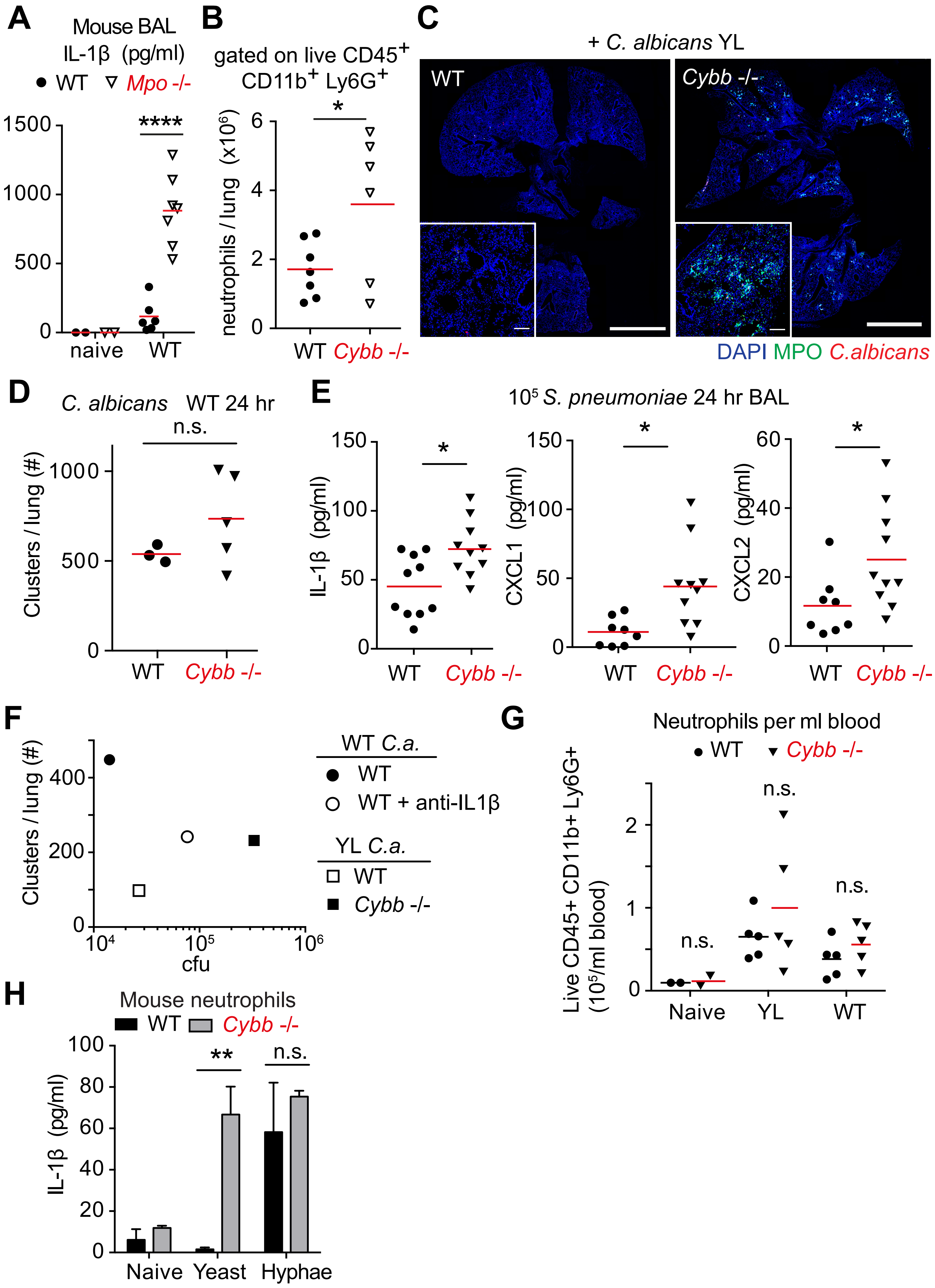

A
Mouse BAL
IL-1β (pg/ml)
WT Mpo -/-
****
naive WT
B
gated on live CD45+ CD11b+ Ly6G+
neutrophils / lung (x10⁶)
*
WT Cybb -/-
C
+ C. albicans YL
WT
Cybb -/-
DAPI MPO C.albicans
D
C. albicans WT 24 hr
n.s.
Clusters / lung (#)
WT Cybb -/-
E
10⁵ S. pneumoniae 24 hr BAL
IL-1β (pg/ml)
CXCL1 (pg/ml)
CXCL2 (pg/ml)
*
WT Cybb -/-
F
Clusters / lung (#)
cfu
WT C.a.
WT
WT + anti-IL1β
YL C.a.
WT
Cybb -/-
G
Neutrophils per ml blood
WT Cybb -/-
Live CD45+ CD11b+ Ly6G+ (10⁵/ml blood)
n.s.
Naive YL WT
H
Mouse neutrophils
WT Cybb -/-
IL-1β (pg/ml)
**
n.s.
Naive Yeast Hyphae

Figure S5, related to Figure 4. Effect of ROS on IL-1β production and neutrophil recruitment.

(A) IL-1β protein in the BAL of MPO-deficient mice infected with WT *C. albicans* 24 hrs post-infection. Each point represents one animal. Statistics by 2-way ANOVA and Sidak's post-test.
(B) Number of neutrophils recruited to the lung of WT and *Cybb* -/- mice analyzed by FACS 24 hrs after infection with yeast-locked (YL) or WT *C. albicans*. Each point represents one animal. Statistics by 2-way ANOVA and Sidak's post-test.
(C) Immunofluorescence micrographs of lung sections of WT and *Cybb* -/- mice infected with YL *C. albicans* 24 hrs post-infection, stained for DNA (DAPI, blue), neutrophils (MPO, green) and *C. albicans* (red). Scale bar: 2.5mm, scale bar of inserts: 100µm.
(D) Number of neutrophil clusters in lungs of WT and *Cybb* -/- mice infected with WT *C. albicans*. Each point represents one animal. Statistics by two-tailed student's t-test.
(E) IL-1β, CXCL1 and CXCL2 protein in the BAL of WT and *Cybb* -/- mice infected with *S. pneumoniae* 24 hrs post infection. Each point represents one animal. Statistics by two-tailed student's t-test.
(F) Number of neutrophil clusters plotted against the corresponding WT or YL *C. albicans* lung burden (CFU) in WT (C57/B6) or *Cybb* -/- mice. IL-1β blocking antibody was added were indicated.
(G) FACS measurement of blood neutrophils in WT versus *Cybb* -/- mice either naive or infected with YL or WT *C. albicans* after 24 hrs. Each point represents one animal. Statistics by 2-way ANOVA and Sidak's post-test.
(H) IL-1β protein production by purified bone-marrow neutrophils from WT or *Cybb* -/- mice exposed to yeast or preformed hyphae. Cytokine production was detected by ELISA. Statistics by 2-way ANOVA and Sidak's post-test.
**** $p < 0.0001$, ** $p < 0.01$, * $p < 0.05$.

Figure S6

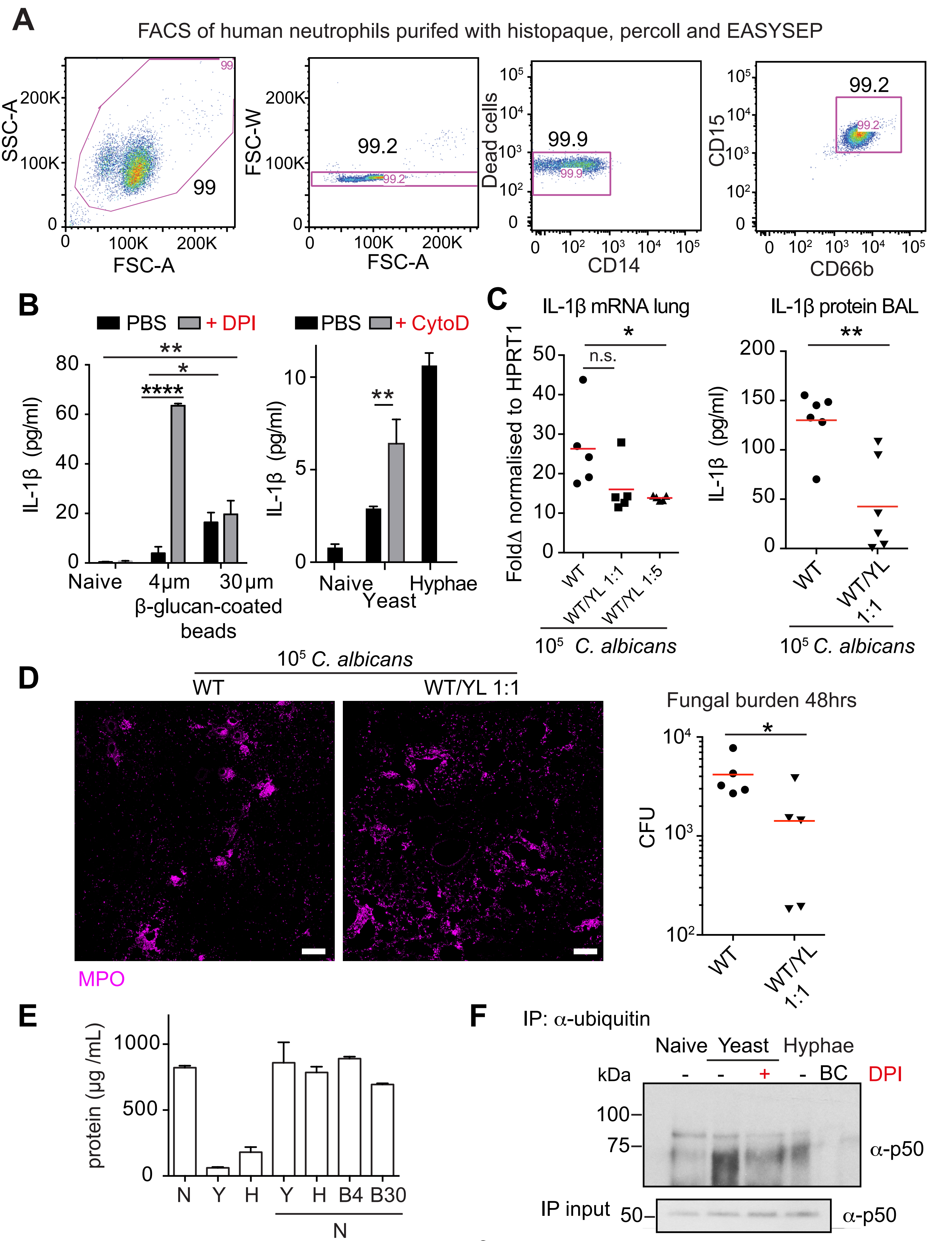

A
FACS of human neutrophils purifed with histopaque, percoll and EASYSEP
SSC-A
FSC-A
FSC-W
Dead cells
CD14
CD15
CD66b
99
99.2
99.9
B
PBS
+ DPI
+ CytoD
IL-1β (pg/ml)
Naive
4μm
30μm
β-glucan-coated beads
Yeast
Hyphae
C
IL-1β mRNA lung
IL-1β protein BAL
FoldΔ normalised to HPRT1
n.s.
WT
WT/YL 1:1
WT/YL 1:5
10⁵ C. albicans
D
WT
WT/YL 1:1
MPO
Fungal burden 48hrs
CFU
E
protein (μg /mL)
N
Y
H
B4
B30
F
IP: α-ubiquitin
Naive
Yeast
Hyphae
kDa
BC
DPI
α-p50
IP input

Figure S6, related to Figures 4, 5 and 6. Effect of microbe size on immune defense and neutrophil signalling.

(A) FACS gating strategy for counting neutrophils purified by Histopaque and Percoll followed by EASYSEP negative selection affinity purification. Neutrophils were gated as live, $CD14^-$, $CD15^+$, $CD66b^+$ cells.
(B) IL-1β protein released by either naive neutrophils or neutrophils treated with 4µm or 30µm wide β-glucan-coated beads (left panel) or yeast and hyphae in the absence or presence of cytochalasin D (right panel). Shown is the mean ±SD of technical duplicates. Statistics by two-way ANOVA and Sidak's post-test. Representative of 3 independent experiments.
(C) IL-1β mRNA in lung or protein in BAL of mice infected with the same inoculum of WT *C. albicans* or a combination of WT and YL strains at 1:1 or 1:5 ratios. Each point represents one animal. Statistics by one-way ANOVA and Tukey's post-test.
(D) Neutrophil clustering in the lung sections of mice from (C) at 24 hrs post infection. Neutrophils were stained for MPO (magenta). Scale bars: 100µm. Fungal burden from the same experiment at 48 hrs post infection. Each point represents one animal. Statistics by one-way ANOVA and Tukey's post-test.
(E) Protein content measured by BCA of neutrophils, yeast or hyphae alone, or neutrophils in combination with either yeast, hyphae at 10:1 MOI (conditions employed in oxidation studies), 4µm or 30µm wide β-glucan-coated beads.
(F) Higher molecular weight, ubiquitinated p50 in lysates of epoxomicin-treated human neutrophils 45 min after exposure to yeast or hyphae untreated or treated with DPI. BC bead control (lysate + beads without anti-ubiquitin antibody). Ubiquitinated proteins were immunoprecipitated with an antibody against ubiquitin, analysed by SDS-PAGE electrophoresis and immunoblotted for p50. p50 content in lysates prior to immunoprecipitation shown below.
**** $p < 0.0001$, ** $p < 0.01$, * $p < 0.05$.

Supplemental experimental procedures

Lung microbe load. After 48 hrs lungs and spleens were homogenized in sterile saline and *C. albicans*-infected tissue plated onto sabourad dextrose agar plates, *S. pneumonia*-infected lungs on blood agar plates.

Depletion studies. Neutrophils were depleted by intraperitoneal administration of 150 µg anti-mouse Ly6G (clone 1A8) or isotype control IgG (clone 2A3) (BioXcell) one day prior to infection and repeated daily. IL-1β was depleted by daily intraperitoneal injections of 100µg anti-mouse IL-1β antibody (clone 1400.24.17, Thermo Scientific) starting 30 min prior to infection.

Analysis of cytokine production in cell culture. 1×10^5 human or mouse neutrophils were plated in 96-well plates and stimulated with *C. albicans*. After 18 hrs supernatant or total cell lysate, obtained by adding 0.1% Nonidet P-40 was collected and cleared by centrifugation at 2000 rpm for 10 minutes. IL-1β content was assessed using mouse and human IL-1β ELISA Ready-SET-Go Kits (eBioscience). **In vivo.** Bronchoalveolar lavage was collected 24 and 48 hrs after infection with 1×10^5 *C. albicans* in 500µl sterile PBS, cleared by centrifugation at 2000 rpm for 10 minutes and stored at -80°C. Cytokine and chemokine protein expression was assayed using the following kits: mouse IL-1β ELISA Ready-SET-Go (eBioscience), mouse IL-6 ELISA (Invitrogen), mouse CXCL1/KC and CXCL2/MIP-2 Quantikine ELSIA kits (R&D) as well as IL-17 Quantikine ELISA kit (R&D) according to the manufacturer's instructions.

Immunoblot analysis. 1×10^6 human peripheral neutrophils were plated in 6-well plates and stimulated with *C. albicans*. At appropriate time points, cells were lysed in SDS sample buffer and separated by SDS-PAGE and western-blotted with anti-human MPO (Dako), anti-human p50 (Millipore), anti-human caspase-1 and IL-1β (R&D) or anti-actin (clone C4, Chemicon) antibodies followed by secondary rabbit anti-mouse IgG or goat anti-rabbit IgG antibodies coupled to HRP (horseradish peroxidase; Thermo Scientific). Oxidized proteins were detected with an OxyBlot™ kit (Chemicon). Samples were lysed in ice-cold

radioimmunoprecipitation assay (RIPA) buffer (50mM Tris-HCl, 150mM NaCl, 1% Nonidet P-40, 0.5% sodium deoxycholate (NaDoc), 0.1% sodium dodecyl sulfate (SDS), 1x Complete (Roche), 1µM Epoxomicin (Calbiochem), 10mM N-ethylmaleimide) and centrifuged at 14000 rpm for 15 min to separate cell debris. Protein concentration was determined using Micro BCA protein assay kit (Pierce) and lysate containing 5µg total protein was employed per reaction.

Immunoprecipitation. 7×10^6 human neutrophils were plated in 10cm cell culture dishes, pre-incubated with 200nM Epoxomicin (Calbiochem) for 1 hr to block degradation of oxidized proteins and stimulated for 1 hr with *C. albicans*. Cells were lysed in RIPA buffer and 150µg whole cell lysate was adjusted to 1ml volume in 20mM Tris-HCl, 10mM ethylenediaminetetraacetic acid (EDTA, Sigma-Aldrich), 100mM NaCl, 1% Nonidet P-40, 1x Complete, 10mM N-ethylmaleimide, 1µM Epoxomicin and 1mg/ml bovine serum albumin. 5µg anti-human p50 (Millipore) or anti-ubiquitin (Thermo Scientific, PA1-187) antibody was added per sample and precipitated over G sepharose 4FF, washed 3X with 0.1% Nonidet P-40/PBS and MilliQ water. For analysis of oxidation beads were taken up in 15µl RIPA buffer and subjected to a modified 2,4-Dinitrophenylhydrazine (DNPH)-derivatisation reaction of the OxyBlot™ protein oxidation detection kit (Chemicon): 6.4µl 20% SDS and 21µl 1x DNPH were added per sample for 15 min at RT. The reaction was terminated with 12.7µl neutralization solution and 5.5µl 1mM Dithiothreitol (DTT). Immune complexes were eluted by incubation at 80°C for 10 min. For analysis of ubiquitination, beads were eluted by adding 2x SDS sample buffer and incubation at 96°C for 5 min. 30µl sample were separated by SDS-PAGE and immunoblotted with the OxyBlot™ protein oxidation detection kit or anti-p50 antibody, respectively.

Subcellular fractionation and p50 binding assay. 5×10^6 human neutrophils were seeded in 10cm cell culture dishes. After stimulation with *C. albicans* for 45 min cells were lysed in 500µl fractionation buffer per plate (250mM sucrose, 20mM 4-(2-hydroxyethyl)-1-piperazineethanesulfonic acid (HEPES), 10mM KCl, 1.5mM $MgCl_2$, 1mM ethylenediaminetetraacetic acid (EDTA), 1mM ethyleneglycoltetraacetic acid (EGTA), 1mM Dithiothreitol (DTT), 1x Complete

(Roche), 1x PhosSTOP (Roche), 1μM Epoxomicin, 10mM N-ethylmaleimide). Lysates were passed 10 times through a 25G needle, incubated for 20 min on ice and centrifuged 5 min at 3000rpm. Pelleted nuclei were washed in 500μl fractionation buffer and resuspended in 50μl RIPA buffer. Nuclear lysate containing 10μg protein was used in a Pierce™ NFκB p50 Transcription Factor Assay (Thermo Scientific).

Quantitative real-time RT-PCR. Total cellular RNA from 1×10^6 human neutrophils in 6-well plates stimulated with *C. albicans* for 1 hr or tissue was isolated using TriReagent-Chloroform extraction followed by isopropanol precipitation (all chemicals from Sigma-Aldrich). 2μg RNA were reverse transcribed to generate cDNA with the Transcriptor high fidelity cDNA synthesis kit from Roche using anchored-oligo$(dT)_{18}$ primer. Gene expression was measured in duplicates using TaqMan Universal PCR Master Mix with gene-specific primers and probes for human and mouse IL-1β, IL-1α and IL-6 as well as HPRT1 or S100A8 on a 7900HT Fast Real Time PCR System (all from Applied Biosystems). The cycling-threshold (CT) for each gene was measured and normalized to that of the housekeeping gene HPRT1 or S100A8, respectively. The relative gene expression was calculated by the change-in-cycling-threshold (ΔΔCT) method using RQ manager software version 1.4 (Applied Biosystems).

Confocal microscopy of fixed cells and tissue. 5×10^4 human peripheral neutrophils were plated on glass coverslips. 1hr after stimulation, cells were fixed for 20 min at 37°C with 2% paraformaldehyde and permeabilized with 0.5% Triton X-100. Cells or lung parafinized tissues fixed in 4% paraformaldehyde were blocked with 2% bovine serum albumin and 2% donkey serum in PBS and incubated with anti-human p47-phox and anti-human p67-phox antibodies (Santa Cruz), anti-human myeloperoxidase (BD Bioscience), anti-mouse myeloperoxidase (R&D) and anti-*C. albicans* (Acris) antibodies followed by Alexa Fluor 488–conjugated donkey anti-mouse, Alexa Fluor 568–conjugated donkey anti-rabbit or Alexa Fluor 568–conjugated donkey anti-goat (Invitrogen), as well as Alexa Fluor 647-conjugated anti-Ly6G (Biolegend) and DAPI (4′,6-diamidino-2-phenylindole dihydrochloride; Life Technologies) before

being mounted in ProLong Gold (Molecular Probes) and imaged by confocal microscopy. For analysis of 200µm thick lung sections, tissue was frozen in OCT compound (VWR), sectioned on a Leica CM3050 S Cryostat, collected in PBS and stained floating before being mounted on glass slides. Images were analyzed with ImageJ v2.0 software. For analysis of neutrophil clustering whole lungs were imaged on an Olympus Slidescanner VS-120 and analysed with OlyVia 2.6 software (Olympus). The lung coverage of clusters was calculated using ImageJ v2.0 software proportional to the area of the whole lung determined by DAPI staining.

ROS and hyphal growth by time-lapse microscopy. 3×10^5 human peripheral neutrophils were plated on glass-bottom Petri dishes (MatTEK) and incubated with heat-inactivated yeast or hyphae at an MOI of 30 and imaged by confocal microscopy every 30 sec at 37°C and 5% CO_2 in the presence of SYTOX (Invitrogen) for NET release or nitroblue tetrazolium chloride (NBT, Sigma-Aldrich) for detection of ROS. Hyphal growth inhibition and the number of associated neutrophils and their NET release were measured by imaging live preformed hyphae and neutrophils every 2 min as above by epifluorescence time-lapse video microscopy. Hyphal length was measured between 2 and 12 hrs of incubation using ImageJ software and correlated to the number of associated neutrophils at 2 hrs.

Flow cytometry. Lungs were harvested, finely minced and digested with 0.4 mg/ml Liberase TL (Roche) for 45 min at 37°C. Subsequently, tissue was meshed through cell strainers to obtain single cell suspensions and erythrocytes were lysed by incubation in ammonium-chloride-potassium (ACK)-lysis buffer for 5 min at room temperature. For intracellular cytokine staining Golgi Stop (BD Bioscience) was added during the isolation of single cells followed by 2 hrs incubation at 37°C. 2×10^6 cells were used for FACS staining with the following antibodies: mouse CD45-APC-Cy7, CD11b-APC (both BD Bioscience), Ly6G-FITC and Ly6C-PE (both purchased from BioLegend) and IL-1β-APC (R&D), human CD14-PE, CD15-Pacific blue and CD66b-FITC (all BioLegend). Dead cells were excluded with the Live/Dead Fixable Aqua Dead Cell Stain Kit purchased from

Life Technologies. Multi-colour cytometry was acquired on BD LSR II Flow Cytometer (BD Bioscience) and analysed with FlowJo v10 (Tree Star) software.

ROS measurements. 1×10^6 human neutrophils were plated in white Nunclon 96-well plates. After 1 hr preincubation at 37°C cells were stimulated with *C. albicans* yeast or preformed hyphae at MOI 2.5. Total ROS production was measured by adding cell-permeable luminol (100µM), extracellular ROS were assessed by adding cell-impermeable isoluminol (100µM) both in the presence of 1.2 U/ml horseradish peroxidase (all chemicals Sigma-Aldrich). Chemiluminescence was measured immediately every 30 seconds over 2 hrs on a FLUOstar Omega plate reader (BMG Labtech).

XTT viability assay. Neutrophils (5×10^3 – 5×10^5) were seeded in 200µl medium in 96-well plates. Subsequently, neutrophils were stimulated with 5×10^4 *C. albicans* preformed hyphae (MOI 10 to 0.1 respectively). At indicated time points 50µl of 6mg/ml 2,3-Bis-(2-Methoxy-4-Nitro-5-Sulfophenyl)-2H-Tetrazolium-5-Carboxanilide (XTT) was added. The plate was incubated for 30 min at 37°C and 100µl of the reaction were transferred into a new 96-well plate and absorbance was read at 450nm. Viability was expressed as fold change compared to *C. albicans* hyphae alone.

Statistical analysis. Statistical significance was assessed by an unpaired, two-tailed Student's *t*-test for single comparison and a two-way analysis of variance (ANOVA) followed by Sidak's multiple comparison post test for multiple comparisons. *P* values of less than 0.05 were considered significant.
