# Supplementary material for: Reactive Oxygen Species Localization Programs Inflammation to Clear Microbes of Different Size
Source: Immunity. 2017 Mar 21;46(3):421–32. doi: 10.1016/j.immuni.2017.02.013 (PMC5965455; doi:10.1016/j.immuni.2017.02.013)
Supplement: Document S2. Article plus Supplemental Information [file mmc2.pdf]

Immunity

Article

Reactive Oxygen Species Localization Programs Inflammation to Clear Microbes of Different Size

Graphical Abstract

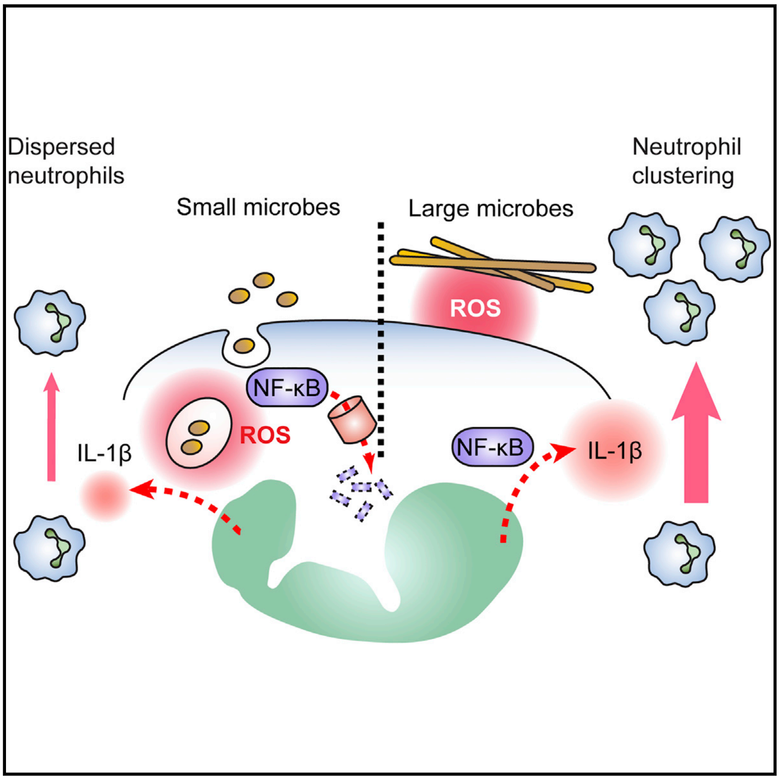

Highlights

- The clearance of microbes of different size involves distinct inflammatory programs
- ROS adjusts neutrophil recruitment to the stoichiometry of host-pathogen interactions
- ROS localization acts as a sensor of microbe size to tune IL-1-β-driven inflammation
- IL-1β amplifies neutrophil recruitment and clustering to clear large microbes

Authors

Annika Warnatsch, Theodora-Dorita Tsourouktsoglou, Nora Branzk, ..., Susanne Herbst, Maximiliano Gutierrez, Venizelos Papayannopoulos

Correspondence

veni.p@crick.ac.uk

In Brief

Inflammation recruits neutrophils to fight invading pathogens of different size. Warnatsch et al. show that reactive oxygen species localization tunes inflammation to compensate for differences in the number of neutrophils required to clear microbes of different size.

Warnatsch et al., 2017, Immunity *46*, 421–432
March 21, 2017 © 2017 The Author(s). Published by Elsevier Inc.
http://dx.doi.org/10.1016/j.immuni.2017.02.013

CellPress

CellPress

Reactive Oxygen Species Localization Programs Inflammation to Clear Microbes of Different Size

Annika Warnatsch,[1] Theodora-Dorita Tsourouktsoglou,[1] Nora Branzk,[1] Qian Wang,[1] Susanna Reincke,[1] Susanne Herbst,[2] Maximiliano Gutierrez,[2] and Venizelos Papayannopoulos[1,3,*]
[1]Antimicrobial Defence Laboratory
[2]Host-Pathogen Interactions in Tuberculosis Laboratory
The Francis Crick Institute, 1 Midland Rd, London NW1 1AT, UK
[3]Lead Contact
*Correspondence: veni.p@crick.ac.uk
http://dx.doi.org/10.1016/j.immuni.2017.02.013

SUMMARY

How the number of immune cells recruited to sites of infection is determined and adjusted to differences in the cellular stoichiometry between host and pathogen is unknown. Here, we have uncovered a role for reactive oxygen species (ROS) as sensors of microbe size. By sensing the differential localization of ROS generated in response to microbes of different size, neutrophils tuned their interleukin (IL)-1β expression via the selective oxidation of NF-κB, in order to implement distinct inflammatory programs. Small microbes triggered ROS intracellularly, suppressing IL-1β expression to limit neutrophil recruitment as each phagocyte eliminated numerous pathogens. In contrast, large microbes triggered ROS extracellularly, amplifying IL-1β expression to recruit numerous neutrophils forming cooperative clusters. Defects in ROS-mediated microbe size sensing resulted in large neutrophil infiltrates and clusters in response to small microbes that contribute to inflammatory disease. These findings highlight the impact of ROS localization on signal transduction.

INTRODUCTION

Neutrophil recruitment is a central aspect of the inflammatory process and is critical for clearing a variety of pathogens. The number of neutrophils at sites of infection must be tightly regulated to ensure that sufficient neutrophils are recruited for efficient clearance while minimizing excess recruitment that drives immune pathology (Medzhitov, 2008). The mechanisms that define the optimum number of neutrophils at sites of inflammation are unknown.

Microbe size plays a critical role in pathogen virulence as with the large invasive filamentous hyphae of *Candida albicans* and *Aspergillus fumigatus* (Brown et al., 2012). Conceivably, microbe size could greatly influence the stoichiometry of host-pathogen interactions. Furthermore, neutrophils may have to act cooperatively to combat large microbes. Yet, whether the clearance of microbes of different size involves different numbers of neutrophils and cooperative strategies is unknown.

Neutrophil recruitment is regulated by pro-inflammatory cytokines such as interleukin-1β (IL-1β) (Amulic et al., 2012). Microbe sensing activates NF-κB to transcribe IL-1β, which inflammasomes process into a mature form (Latz et al., 2013; Netea et al., 2008; Plato et al., 2015). In turn, IL-1β upregulates interleukin-17 (IL-17) and chemokines such as CXCL1 and CXCL2 to recruit neutrophils (Miller et al., 2006, 2007; Park et al., 2005). Fungal hyphae activate the inflammasome more potently than yeast in isolated macrophages but the in vivo relevance of this disproportionate response has not been investigated (Joly et al., 2009; Saïd-Sadier et al., 2010). Furthermore, differential cytokine expression in macrophages and dendritic cells has been attributed to the selective activation of innate immune receptors (Gantner et al., 2005; Kashem et al., 2015; van der Graaf et al., 2005).

Recent work has implicated neutrophils in IL-1β production upon bacterial infection (Chen et al., 2014; Cho et al., 2012; Karmakar et al., 2015), suggesting that neutrophils could play more central roles in modulating inflammation. In comparison to other phagocytes, neutrophils generate higher concentrations of ROS (Devalon et al., 1987; Nathan and Shiloh, 2000; Silva, 2010). In addition to their cytotoxic role, ROS regulate cell signaling (Murphy et al., 2011) and inhibit inflammasome activation and cytokine expression (Bagaitkar et al., 2015; Han et al., 2013; Harbort et al., 2015; Huang et al., 2015; Meissner et al., 2008, 2010; Morgenstern et al., 1997). These observations are consistent with hyper-inflammatory pathology in chronic granulomatous disease (CGD) caused by mutations in the NOX2 NADPH oxidase (Rieber et al., 2012). However, the physiological purpose of this oxidative regulatory mechanism is still unknown. Using microbes with varying sizes, we have demonstrated that the ability to sense the differential localization of ROS allows neutrophils to adjust inflammation by modulating their own recruitment and cooperation to effectively clear microbes of different size.

RESULTS

Microbe Size Regulates Neutrophil Recruitment and Clustering

To examine the impact of microbe size on neutrophil responses, we took advantage of the fungal pathogen *C. albicans*, which grows as small yeast or as large filamentous hyphae. First, we investigated how microbe size impacts the stoichiometry of the interaction between neutrophils and *C. albicans* yeast and

CrossMark

This is an open access article under the CC BY license (http://creativecommons.org/licenses/by/4.0/).

CellPress

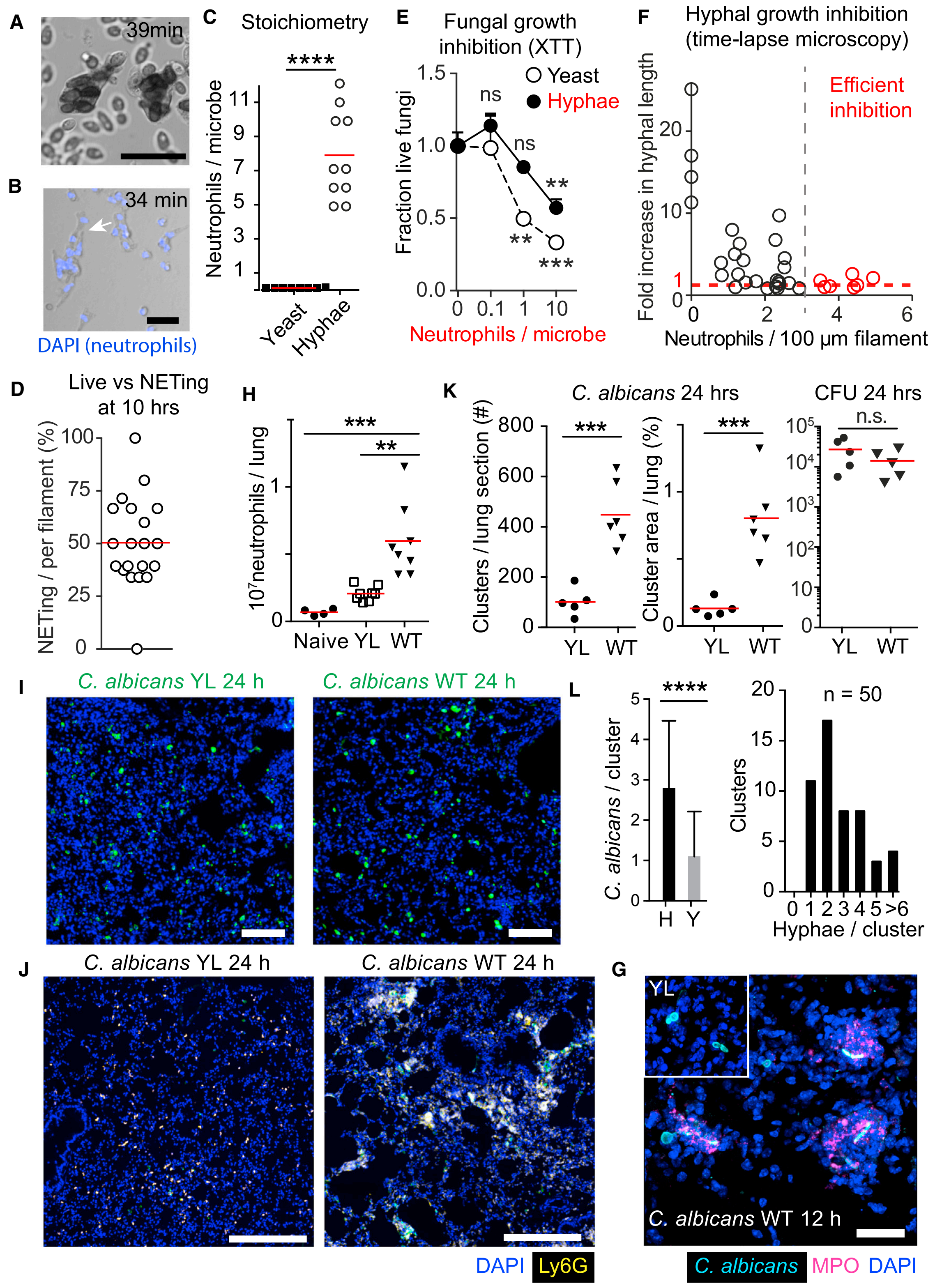

Figure 1. Microbe Size Regulates Inflammation

(A and B) Time-lapse microscopy of neutrophils during infection with *C. albicans* yeast (MOI 40) (A) and hyphae (B). Extracellular DNA stained with Sytox Green (A and B) and total DNA with DAPI (B) (blue). Scale bars represent 10 μm (A) and 100 μm (B). Representative of three (A) and five (B) experiments.

(C) Ratio of neutrophils interacting with *C. albicans* yeast or hyphae. Statistics by two-tailed Student's t test.

(legend continued on next page)

hyphae by time-lapse video microscopy (Figures 1A and 1B). On average, neutrophils ingested 8 yeast particles (Figure 1C), whereas each 100-μm-long hyphal filament was engaged by 8 neutrophils exhibiting a 100-fold difference in stoichiometry (Figure 1C). Half of the neutrophils interacting with hyphae released neutrophil extracellular traps (NETs). The rest of the population remained alive for at least 10 hr (Figure 1D). NETs control hyphae (Bianchi et al., 2011; Branzk et al., 2014) but we suspected that long-lived neutrophils might play a regulatory role.

To determine whether the clearance of yeast and hyphae required different numbers of neutrophils, we monitored their impact on the viability of a yeast-locked (YL) *C. albicans* mutant that is unable to form hyphae (Δ*hgc1*) or pre-formed WT hyphae. Neutrophils restricted yeast viability at a 1:1 ratio. In contrast, a 10:1 ratio of neutrophils to hyphae was required to control hyphae (Figure 1E). To define the number of neutrophils required to control hyphae more accurately, we examined filament growth suppression by time-lapse microscopy. At least 3 neutrophils per 100 μm filament were needed for efficient growth suppression (Figure 1F). Therefore, few neutrophils were required to clear small yeast and a higher number to control large hyphae.

Next, we examined whether neutrophil recruitment in vivo depended on microbe size. In the lung, WT fungi switch to hyphae but the YL mutant remained in the yeast form (Figure 1G). Neutrophil recruitment occurred within 24 hr of infection, whereas fungal clearance took place 24–48 hr after infection. Infection with WT *C. albicans* yielded a 3-fold higher number of infiltrating neutrophils compared to the YL *Δhgc1* strain (Figures 1H and S1A). While both strains dispersed homogeneously at 24 hr (Figure 1I), the lungs of mice infected with WT *C. albicans* contained neutrophils organized in large clusters (Figures 1J, 1K, and S1B) predominately around hyphae (Figures 1G and 1L). In contrast, the lungs of mice infected with the YL strain contained significantly fewer neutrophil clusters, with neutrophils being more evenly dispersed, despite bearing a comparable fungal load at 24 hr after infection (Figure 1K). These data pointed to an unknown mechanism that adjusts neutrophil recruitment and organization to the distinct requirements associated with microbes of different size.

IL-1β Regulates Neutrophil Recruitment and Clustering

To understand how neutrophil clustering is regulated, we first examined whether microbe size regulated cytokine responses. YL *C. albicans* infection triggered reduced IL-1β, IL-6, CXCL-1, and CXCL-2 in the bronchoalveolar lavage (BAL) after 24 hr (Figures 2A, S2A, and S2B) and IL-17 in circulation after 48 hr (Figure 2B). Consistently, CXCL-2 expression concentrated around neutrophil clusters only in mice infected with WT *C. albicans* (Figure 2C). Then, we investigated whether the clearance of yeast and hyphae had different IL-1β requirements using a blocking antibody that depleted IL-1β efficiently in vivo (Figure S2C). IL-1β blockade had a minor effect on the clearance of the YL strain (Figure 2E) as both isotype and anti-IL-1β-treated mice made a full recovery (Figure 2D). In contrast, WT *C. albicans* infection induced persistent weight loss and increased pulmonary fungal load at 48 hr, upon IL-1β suppression (Figures 2D and 2E). Consistently, IL-1β blockade reduced neutrophil recruitment (Figure 2F) and clustering (Figures 2G and S3A) in response to WT *C. albicans* or *A. fumigatus* infection (Figures S3B–S3D) despite the trend for an increase in the fungal load already by 24 hr (Figure 2G, right). Therefore, IL-1β signaling was critical for amplifying neutrophil recruitment and clustering to clear hyphae but was dispensable against yeast.

Neutrophils Are Required for Selective IL-1β Induction

To confirm that IL-1β controlled hyphae by regulating neutrophils, we tested whether depleting neutrophils would also differentially affect the clearance of the two fungal forms. Neutrophil depletion (Figure S4A) did not affect the YL yeast clearance (Figure 3A) but resulted in higher fungal load upon infection with WT *C. albicans* hyphae 48 hr after infection. To examine whether neutrophils contributed to IL-1β production, we measured IL-1β upon neutrophil depletion. Neutrophil depletion abrogated IL-1β protein release and mRNA expression and attenuated CXCL-1 and CXCL-2 in response to *C. albicans* infection (Figures 3B and S4B). Confirming that neutrophils produce IL-1β in a microbe-size-dependent manner in vivo, we detected a higher number of IL-1β-positive neutrophils in the lungs of mice infected with WT *C. albicans* than mice infected with the YL strain, in the absence of restimulation (Figures 3C and S4C). Similarly, affinity-purified neutrophils from mice infected with WT *C. albicans*

(D) Percent of total neutrophils attached to fungal filaments that released NETs.

(E) *C. albicans* growth inhibition by neutrophils compared to fungus alone monitored by an enzymatic XTT assay at different neutrophil to microbe ratios. Data are means ± SD of technical duplicates. Representative of three independent experiments. Statistics by two-tailed Student's t test comparing changes over baseline (*C. albicans* alone).

(F) Fold increase in hyphal length during a 10 hr incubation (at 12 hr) plotted against the number of attached neutrophils at 2 hr, analyzed by time-lapse microscopy.

(G) Immunofluorescence confocal micrographs depicting YL and WT *C. albicans* hyphae (cyan) and MPO-positive neutrophils (magenta) in thick lung sections 12 hr after infection. Scale bar represents 30 μm.

(H) Number of neutrophils in lungs of naive WT mice or 24 hr after infection with yeast-locked (YL) or WT *C. albicans*, measured by FACS, gated on live $CD45^+$, $CD11b^+$, and $Ly6G^+$. Each point represents one animal. Statistics by one-way ANOVA and Tukey's multiple comparison post-test. Please also see Figure S1A.

(I) WT or yeast-locked (YL) *C. albicans* (green) distribution in lungs after 24 hr. Scale bars represent 100 μm.

(J) Immunofluorescence confocal micrographs depicting dispersed and clustering neutrophils in lung sections of mice infected with yeast-locked or WT *C. albicans*, 24 hr after infection stained for DNA (DAPI, blue), neutrophils (Ly6G, yellow), and *C. albicans* (green). Scale bars represent 250 μm. Please also see Figure S1B.

(K) Number of neutrophil clusters, average cluster area per lung section, and *C. albicans* colony forming units (cfu) 24 hr after infection with YL or WT *C. albicans*. Each point represents one animal. Statistics by two-tailed Student's t test.

(L) Number of yeast (Y) and hyphae (H) in 50 randomly selected neutrophil clusters in mice infected with WT *C. albicans* (left) and the distribution of clusters with respect to the number of hyphae per cluster (right). Data are means ± SD.

Statistics by two-tailed Student's t test. ****$p < 0.0001$, ***$p < 0.001$, **$p < 0.01$.

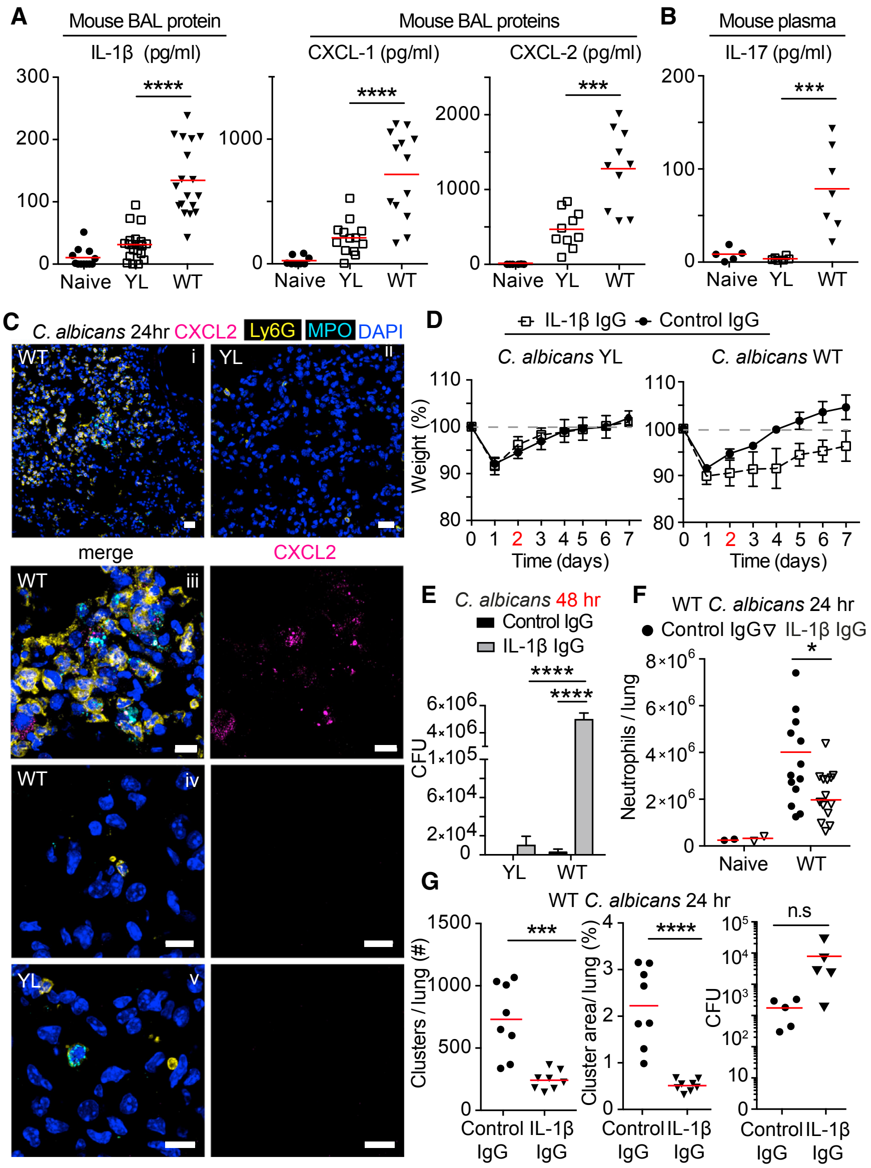

Figure 2. IL-1β Is Critical for Clustering to Control Hyphae

(A and B) Cytokine analysis in WT mice after intratracheal infection with 10^5 yeast-locked (YL) or WT *C. albicans*. IL-1β, CXCL-1, and CXCL-2 protein in the BAL 24 hr after infection (A). IL-17 protein in plasma 48 hr after infection (B). Each point represents one animal. Statistics by one-way ANOVA followed by Tukey's multiple comparison post-test. Please also see Figures S2A and S2B.

(C) Immunofluorescence confocal micrographs of lung sections from WT mice infected with 10^5 yeast-locked (YL; ii, zoomed image in v) or WT *C. albicans* (i, zoomed image in iii, zoomed image from area with dispersed neutrophils in iv) after 24 hr, stained for DNA (DAPI, blue), neutrophils (Ly6G, yellow), and CXCL2 (magenta) and MPO (cyan). Scale bars in (i) and (ii) represent 20 μm; in (iii)–(v) 10 μm.

(D–G) WT mice treated with isotype control or anti-IL-1β neutralizing antibody after intratracheal infection of 10^5 yeast-locked (YL) or WT *C. albicans* 24 hr after infection. Please also see Figure S2C.

(D) Mean weight monitoring for seven animals per group. Representative of two experiments. Data are means ± SD.

(E) *C. albicans* colony forming units (cfu) in the lung 48 hr after infection. Mean ± SD of six animals per group. Statistics by two-way ANOVA followed by Sidak's multiple comparison post-test.

(F) Number of neutrophils per lung analyzed by FACS. Each point represents one animal. Statistics by two-way ANOVA followed by Sidak's multiple comparison post-test.

(G) Number of neutrophil clusters, average cluster area per lung section, and fungal burden. Each point represents one animal. Please also see Figure S3A.

Statistics by two-tailed Student's t test. ****$p < 0.0001$, ***$p < 0.001$, *$p < 0.05$.

contained higher concentrations of IL-1β mRNA and protein than neutrophils purified from mice infected with the yeast-locked strain (Figure 3D). These data identified neutrophils as important sources of IL-1β in fungal infection, adjusting cytokine production according to the size of the microbe they encountered.

ROS Localization Regulates IL-1β-Driven Neutrophil Clustering

Next, we explored how microbe size tunes cytokine expression. Neutrophil-derived NETs regulate IL-1β expression during sterile inflammation (Warnatsch et al., 2015). NETs are selectively induced by hyphae (Branzk et al., 2014) and could amplify IL-1β induction in a microbe-size-specific manner. To test this hypothesis, we employed mice deficient in the ROS-producing enzyme myeloperoxidase (MPO) that is required for NET formation (Branzk et al., 2014; Kolaczkowska et al., 2015; Metzler et al., 2011, 2014; Papayannopoulos et al., 2010). MPO deficiency led to elevated pulmonary IL-1β expression in response to WT *C. albicans* (Figure S5A) indicating that during infection, neutrophils upregulate IL-1β production in an alternative ROS-dependent manner. Consistently, infection with YL *C. albicans* strains yielded elevated IL-1β protein in *Cybb*-deficient mice, which lack the gp91phox subunit of the NADPH oxidase and do not produce superoxide (Figure 4A; Pollock et al., 1995), and a 9-fold upregulation of IL-1β transcript compared to only a 2-fold non-significant increase in response to WT *C. albicans* (Figure 4B) likely due to a minor yeast population (Figure 1L). Therefore, ROS suppressed cytokine transcription in a microbe-size-dependent manner in vivo.

Since elevated IL-1β expression drove neutrophil clustering, we examined whether the lack of ROS would upregulate neutrophil clustering in response to YL *C. albicans*. Consistently, neutrophil numbers (Figure S5B) and clustering were significantly higher in *Cybb*-deficient animals infected with YL *C. albicans* (Figures

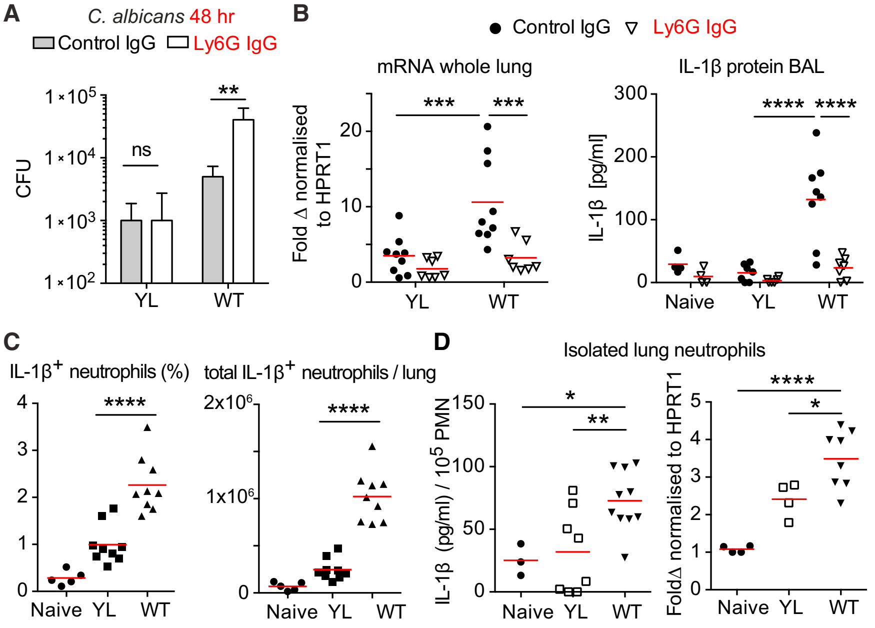

Figure 3. Neutrophils Produce IL-1β in a Microbe-Size-Dependent Manner

(A and B) WT mice treated with isotype control IgG or anti-Ly6G antibody prior to intratracheal infection with 10^5 yeast-locked (YL) or WT *C. albicans*. Please also see Figure S4A.

(A) *C. albicans* burden in the lung 48 hr after infection. Mean ± SD of three animals per group. Statistics by two-way ANOVA followed by Sidak's multiple comparison post-test.

(B) IL-1β mRNA in the lung normalized to HPRT1 expression and IL-1β protein expression in bronchoalveolar lavage 24 hr after infection. Each point represents one animal. Statistics by two-way ANOVA followed by Sidak's multiple comparison post-test.

(C) Fraction and total number of lung neutrophils positive for intracellular IL-1β staining measured by FACS. Neutrophils were gated as live $CD45^+$, $CD11b^+$, $Ly6G^+$ cells. Each point represents one animal. Statistics by one-way ANOVA followed by Tukey's multiple comparison post-test. Please also see Figure S4C.

(D) IL-1β protein and mRNA in neutrophils isolated by negative selection from lungs of WT mice infected with 10^5 *C. albicans* YL or WT. Each point represents one animal. Statistics by one-way ANOVA followed by Tukey's multiple comparison post-test.

$^{****}p < 0.0001$, $^{***}p < 0.001$, $^{**}p < 0.01$, $^{*}p < 0.05$.

4C and S5C). The elevated fungal burden in *Cybb*-deficient mice is consistent with impaired phagocytosis and pathogen clearance due to the lack of ROS. In contrast there was no increase in clustering in *Cybb*-deficient mice upon infection with WT *C. albicans* (Figure S5D). Similarly, neutrophil clusters were nearly absent in the lungs of WT mice infected with the small bacterium *S. pneumonia* 24 hr after infection but potent clustering was elicited in *Cybb*-deficient animals (Figure 4D) along with elevated IL-1β, CXCL1, and CXCL2 expression without a significant increase in bacterial burden (Figures 4D, right, and S5E). Overall, we found no correlation between fungal burden and neutrophil clustering at 24 hr but consistent correlation with microbe size, IL-1β signaling, or the absence of ROS (Figure S5F). Moreover, blood circulating neutrophil numbers were identical in *Cybb*-deficient mice (Figure S5G). Together, these data indicate that ROS regulated neutrophil clustering by selectively suppressing cytokine expression in response to small microbes, rather than via the amplification of expression in response to large hyphae.

To investigate whether neutrophils were sufficient to generate IL-1β in a microbe-size-dependent manner, we incubated histopaque- and percoll-purified primary human neutrophils with YL yeast or WT pre-formed hyphae in vitro. Hyphae triggered potent IL-1β expression and protein release into cell culture supernatants within 4 hr of stimulation. By comparison, YL *C. albicans* triggered significantly lower concentrations of IL-1β (Figure 4E). Pretreatment of neutrophils with the NOX2 inhibitor diphenyleneiodonium (DPI) led to a substantial increase in IL-1β protein release upon infection with YL yeast to concentrations that were comparable to induction by WT hyphae. In contrast, DPI had no effect on cytokine release by neutrophils activated with hyphae. Similar results were obtained with murine bone marrow neutrophils isolated from WT or *Cybb*-deficient mice (Figure S5H). Since the differential cytokine expression was completely abrogated in the absence of ROS, these data pointed to the absence of mechanisms that depend on differences in microbe surface molecules.

To confirm the dependence of cytokine expression on microbe size rather than other factors such as difference in microbial surface molecules or released metabolites, we incubated human neutrophils with fragmented hyphae of sizes comparable to yeast. Fragmentation of hyphae disrupted their ability to potently upregulate mature IL-1β protein and transcript (Figure 4F). The ability of fragmented hyphae to induce IL-1β release was rescued by blocking NOX2 activity with DPI, indicating that hyphal fragments had not lost their ability to activate neutrophils but instead had suppressed cytokine expression in a ROS-dependent manner (Figure 4G). Moreover, using ultra-pure human neutrophils (Figure S6A), we found that phagocytosis of small 4 μm beads blocked cytokine expression in response to hyphae in a ROS-dependent manner (Figure 4H, left). Similarly, large *M. bovis* Bacillus Calmette-Guérin (BCG) aggregates, but not single bacteria, upregulated IL-1β and *Saccharomyces cerevisiae cbk1Δ* mutants, which fail to separate during cell division and form large multinucleated cells (Racki et al., 2000), selectively induced IL-1β expression compared to a control single-cell strain. Importantly, inhibiting the ROS burst with DPI restored IL-1β upregulation in response to small BCG and *S. cerevisiae* single cells (Figure 4H, middle and right). Furthermore, large β-glucan-coated beads induced higher cytokine expression than small beads, where inhibition of expression could be alleviated with DPI. This ROS-mediated suppression of cytokine expression depended on the phagocytosis of small

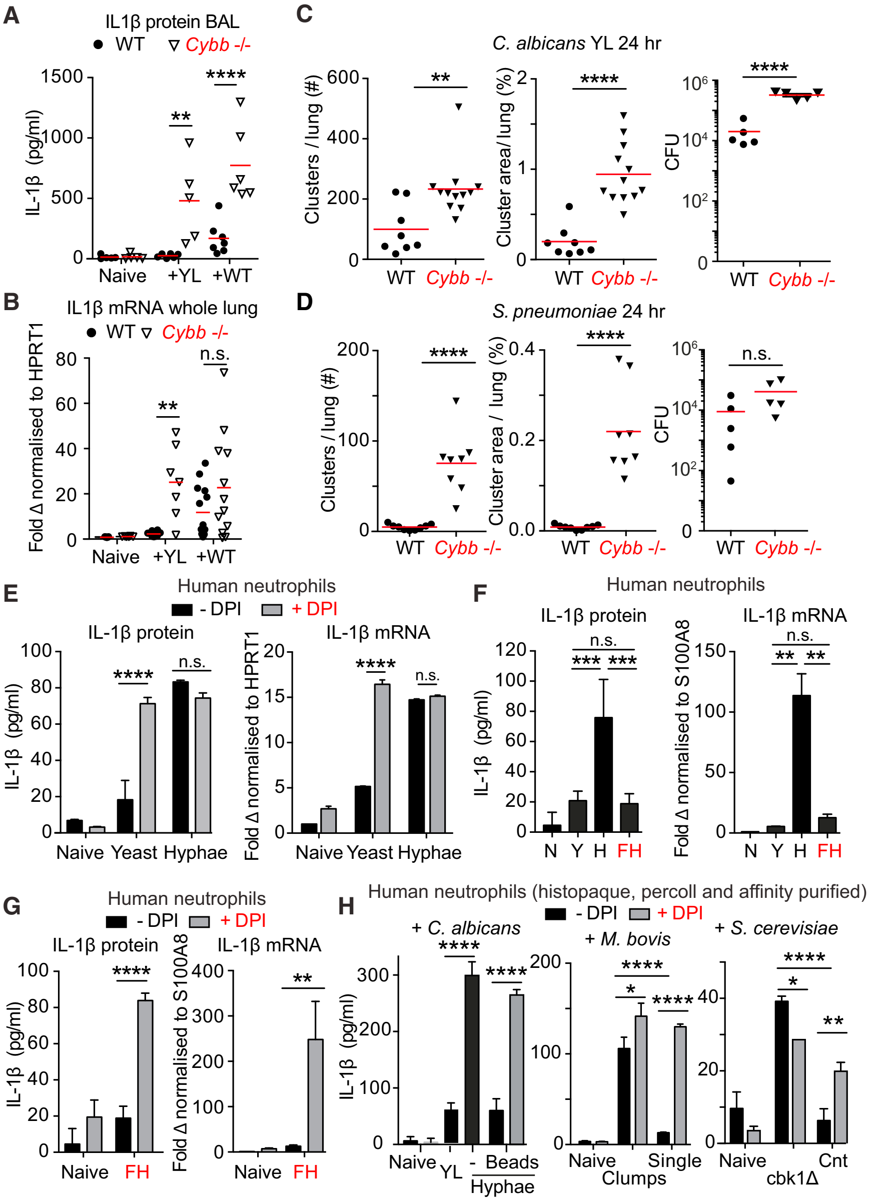

Figure 4. ROS Regulates Inflammation in a Microbe-Size-Dependent Manner

(A and B) IL-1β protein in the BAL (A) and mRNA in whole lung (B) of WT and *Cybb*$^{-/-}$ mice, 24 hr after infection with 10^5 yeast-locked (YL) or WT *C. albicans*. Each point represents one animal. Statistics by two-way ANOVA followed by Sidak's multiple comparison post-test.

(C and D) Number of neutrophil clusters, average cluster size per lung section, and microbe burden 24 hr after infection in WT and *Cybb*$^{-/-}$ mice infected with (C) 10^5 yeast-locked (YL) *C. albicans* or (D) 10^4 *S. pneumonia*. Each point represents one animal. Statistics by two-tailed Student's t test. Please also see Figures S5C and S5E.

(E) IL-1β protein released after 4 hr (left) and IL-1β transcript after 1 hr (right) from purified human neutrophils untreated or treated with DPI and yeast or preformed hyphae.

(F) IL-1β protein after 4 hr and transcript after 1 hr from naive purified human neutrophils or treated with yeast (Y), hyphae (H), or fragmented hyphae (FH).

(G) IL-1β protein after 4 hr and transcript after 1 hr in naive purified human neutrophils untreated or treated with DPI or fragmented hyphae (FH).

(H) IL-1β protein released by neutrophils purified over Histopaque and Percoll as well as an affinity-based negative selection kit (99.2% pure, please see Figure S6A). Naive neutrophils or activated with *C. albicans* yeast or hyphae in the presence of 4 μm opsonized polystyrene-coated beads (left), with single bacteria or large aggregates of *M. bovis* BCG (middle), or with aggregates of *cbk1Δ S. cerevisiae* or single cells of the control strain (right) in the absence or presence of DPI. Total IL-1β protein was measured in response to *S. cerevisiae*.

(E–H) Stimulation at MOI 10. Data are means ± SD of technical duplicates. Representative of three independent experiments.

Statistics by two-way ANOVA followed by Sidak's multiple comparison post-test. ****$p < 0.0001$, ***$p < 0.001$, **$p < 0.01$, *$p < 0.05$.

particles because treatment with cytochalasin D, which blocks phagocytosis, upregulated mature IL-1β release in response to yeast (Figure S6B). These data uncover a phagocytosis-dependent oxidative mechanism that enables neutrophils to tune cytokine production to microbe size. Consistently, coinfection with YL fungi reduced cytokine expression (Figure S6C) and led to more diffuse neutrophil clustering in response to WT fungi in vivo (Figure S6D), confirming the ability of phagocytosis to suppress inflammation.

Microbes of Different Size Induce ROS at Different Locations

Next, we explored whether there were fundamental differences in the ROS burst elicited by small and large microbes via two chemiluminescent probes: luminol, which is cell permeable and detects both intracellular and extracellular ROS, and isoluminol, which detects only extracellular ROS. Both yeast and hyphae elicited a potent ROS burst, albeit with somewhat different dynamics. However, only hyphae were able to induce strong extracellular ROS (Figure 5A). The initial delay in ROS detection in response to yeast during the reaction was consistent with ROS being consumed intracellularly. In contrast, in response to hyphae, ROS were released extracellularly where they could interact more efficiently with luminol. To confirm this hypothesis, we detected ROS by monitoring nitro blue tetrazolium (NBT) oxidation by microscopy. Neutrophils phagocytizing yeast exhibited slightly slower ROS dynamics but nevertheless yielded a powerful and persistent ROS burst within 20–60 min (Figure 5B). In contrast, neutrophils interacting with hyphae yielded faster but highly transient ROS bursts that relocated dynamically as the points of contact between neutrophil and filament shifted (Figure 5B, white arrows). Next, we investigated the impact

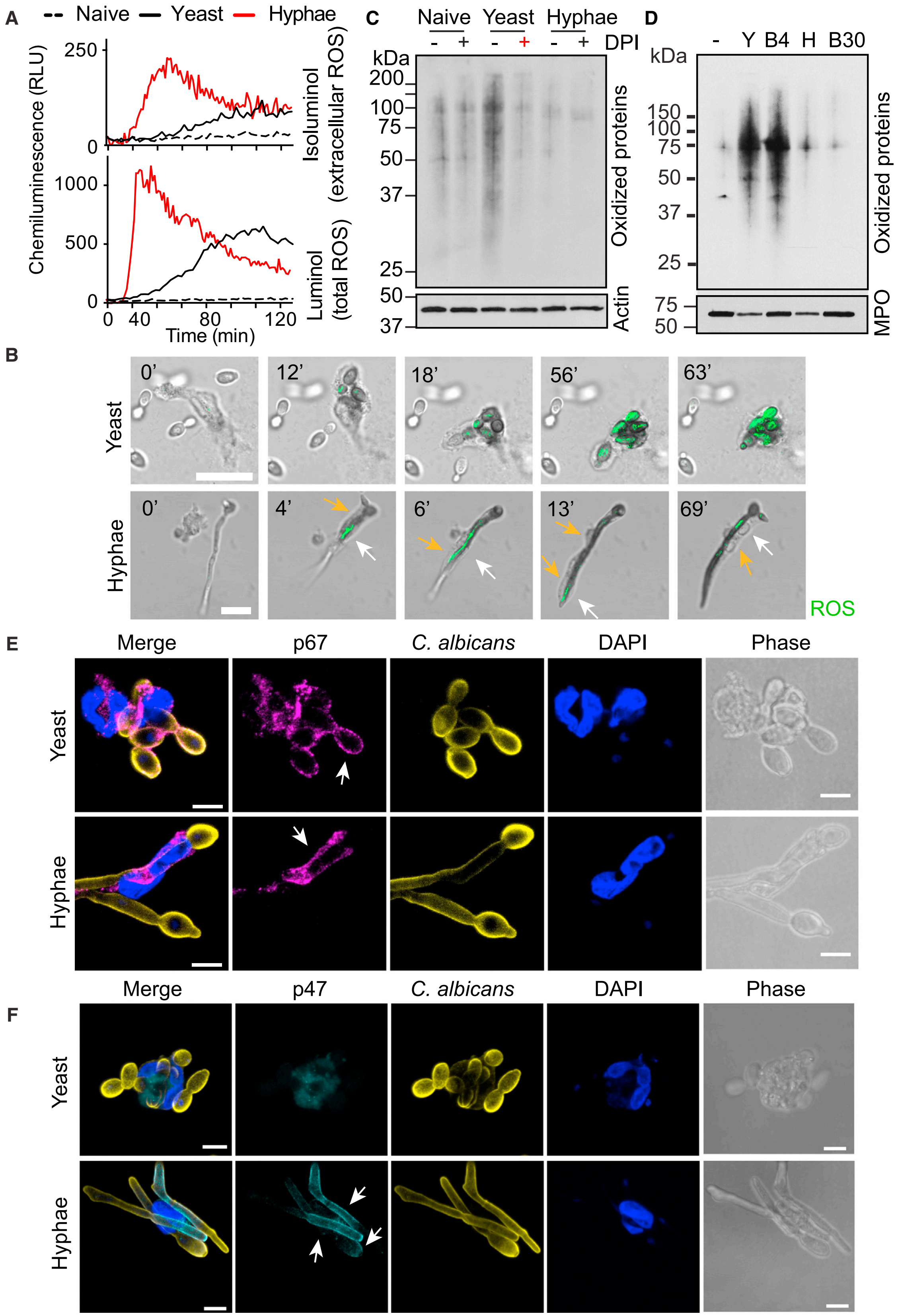

(legend on next page)

of ROS localization on protein oxidation by analyzing protein carbonylation in neutrophil lysates. Yeast triggered potent protein oxidation, which was blocked by DPI and absent in neutrophils incubated with hyphae (Figure 5C). The vast majority of the total protein content was of neutrophil origin in these reactions (Figure S6E), suggesting that it is the neutrophil and not fungal proteins being oxidized. This hypothesis was validated by the strong protein oxidation of neutrophil proteins in cells treated with small 4 μm β-glucan-coated beads. Large 30 μm beads or hyphae did not trigger neutrophil protein oxidation (Figure 5D).

To uncover the basis for the differential ROS localization, we examined the localization of the NADPH oxidase. During neutrophil activation, cytoplasmic NOX2 oxidase subunits associate with membrane-bound subunits to assemble the active oxidase enzyme. p67phox colocalized both with the plasma membrane regions contacting hyphae and with phagosomal membranes surrounding yeast (Figure 5E). p47phox associates with the NADPH oxidase complex on the plasma membrane, but not the phagosome (Ueyama et al., 2007). As neutrophils stretched in an attempt to phagocytize hyphae, the NADPH oxidase complex assembled selectively on plasma membrane areas contacting hyphae as indicated by the co-localization of p47phox with the fungal cell wall staining (Figure 5F, arrows). In contrast, p47phox did not colocalize with phagocytized yeast. Hence, the differential assembly of the NADPH oxidase allowed yeast to trigger intracellular ROS driving potent cellular oxidation and hyphae to elicit ROS extracellularly, sparing neutrophil proteins from oxidation.

ROS Localization Regulates NF-κB in a Microbe-Size-Dependent Manner

To investigate whether ROS regulated cytokines in a microbe-size-dependent manner at the transcriptional level, we incubated human neutrophils with the translation inhibitor cycloheximide (CHX) and stimulated them with yeast or hyphae. CHX efficiently blocked mature IL-1β protein release in response to hyphae as well as yeast upon NOX2 inhibition with DPI (Figure 6A). Furthermore, TPCA-1, an inhibitor of IKK1 and IKK2 kinases upstream of NF-κB, also suppressed mature IL-1β release in DPI-treated neutrophils in response to yeast (Figure 6A). These data suggest that ROS suppresses NF-κB activity selectively in response to yeast and hyphae. IL-1β is transcribed by a heterodimer composed of the NF-κB p50 and p65 subunits. Among all the potential targets for oxidation in the pathway, we focused on p50 because oxidation inhibits its transcriptional activity (Pineda-Molina et al., 2001; Toledano and Leonard, 1991) and would override any upstream regulatory events. Assuming ROS might regulate IL-1β release by interfering with NF-κB signaling directly, we analyzed p50 protein expression in neutrophil lysates 30 min after *C. albicans* exposure. In neutrophils treated with YL *C. albicans*, p50 was not upregulated. In contrast, p50 was strongly upregulated in response to hyphae (Figure 6B). Inhibiting the ROS burst with DPI was sufficient to partially restore p50 in yeast-infected neutrophils. To examine whether the partial restoration of p50 expression in DPI-treated neutrophils was sufficient to restore NF-κB transcriptional activity, we employed a transcription factor-binding assay that measures the binding of active p50 to its consensus sequence. In contrast to neutrophils incubated with hyphae, p50 activity was not upregulated in yeast-treated neutrophils (Figure 6C). Inhibition of ROS production was sufficient to restore p50 transcriptional activity in yeast-treated cells comparable to neutrophils treated with hyphae (Figure 6C). Hence, upon phagocytosis of small microbes, intracellular oxidation attenuates NF-κB activity.

To establish whether p50 was differentially oxidized and degraded in response to stimulation with different fungal forms, we pretreated neutrophils with the proteasome inhibitor epoxomicin prior to incubation with *C. albicans*, which was sufficient to prevent p50 degradation (Figure 6D, IP input). Subsequent to fungal exposure, we collected neutrophil lysates, immunoprecipitated p50, and analyzed its oxidation. Treatment with yeast resulted in potent p50 oxidation (Figure 6D) and ubiquitination (Figure S6F) that could be blocked with DPI and restored to concentrations that were comparable to neutrophils stimulated with hyphae. These data confirmed that yeast but not hyphae drove ROS-mediated p50 oxidation, targeting the protein for degradation. Thus, NF-κB oxidation and transcriptional activity depend on the differential localization of ROS.

Finally, we investigated which neutrophil enzymes process IL-1β into its mature p17 form in response to hyphae using pharmacological inhibitors. Inhibitors of neutrophil elastase (NE) and cathepsin G (CG) did not affect IL-1β release, but caspase inhibition with z-VAD-fmk completely abolished cytokine production (Figure 6E), confirming macrophage data showing that ROS regulate IL-1β production by suppressing caspase-1 (Meissner et al., 2008, 2010). However, the effect of ROS on the inflammasome has been demonstrated with soluble agonists in macrophages that generate only a fraction of the ROS produced by neutrophils. Therefore, we investigated how the differential ROS localization generated by live fungi affected inflammasome activation in neutrophils. Pro-caspase-1 was cleaved into its active p10 subunit upon incubation with hyphae, accompanied by maturation of the p17 IL-1β subunit. Inflammasome activation

Figure 5. Differential ROS Localization Induces Selective Neutrophil Protein Oxidation in a Microbe-Size-Dependent Manner

(A) ROS production by naive human blood-derived neutrophils or in the presence of YL yeast or WT preformed hyphae, detected by chemiluminescence of the cell-impermeable isoluminol to detect extracellular ROS (top) or by the cell-permeable luminol to detect both intracellular and extracellular ROS (bottom), in the presence of exogenous horseradish peroxidase to detect superoxide production.

(B) Representative time-lapse microscopy depicting neutrophil ROS production in response to yeast or hyphae monitored by NBT staining. Dynamic ROS production in response to hyphae denoted by white arrows. Neutrophil cell body indicated by yellow arrows. Scale bars represent 10 μm.

(C) Carbonylated proteins in lysates of human neutrophils 2 hr after incubation with yeast (YL) or hyphae untreated or treated with DPI and analyzed by western immunoblotting. Representative of five independent experiments.

(D) Carbonylated proteins in lysates of human neutrophils treated with either yeast (Y), hyphae (H), 4 μm (B4), or 30 μm (B30) β-glucan-coated beads.

(E and F) Phase contrast and immunofluorescence staining of human neutrophils interacting with yeast or hyphae stained for (E) p67-phox (magenta), *C. albicans* (yellow), DNA (DAPI, blue), or (F) p47-phox (cyan). Arrows indicate assembly of the NADPH oxidase components on membranes associated with *C. albicans*. Scale bars represent 5 μm.

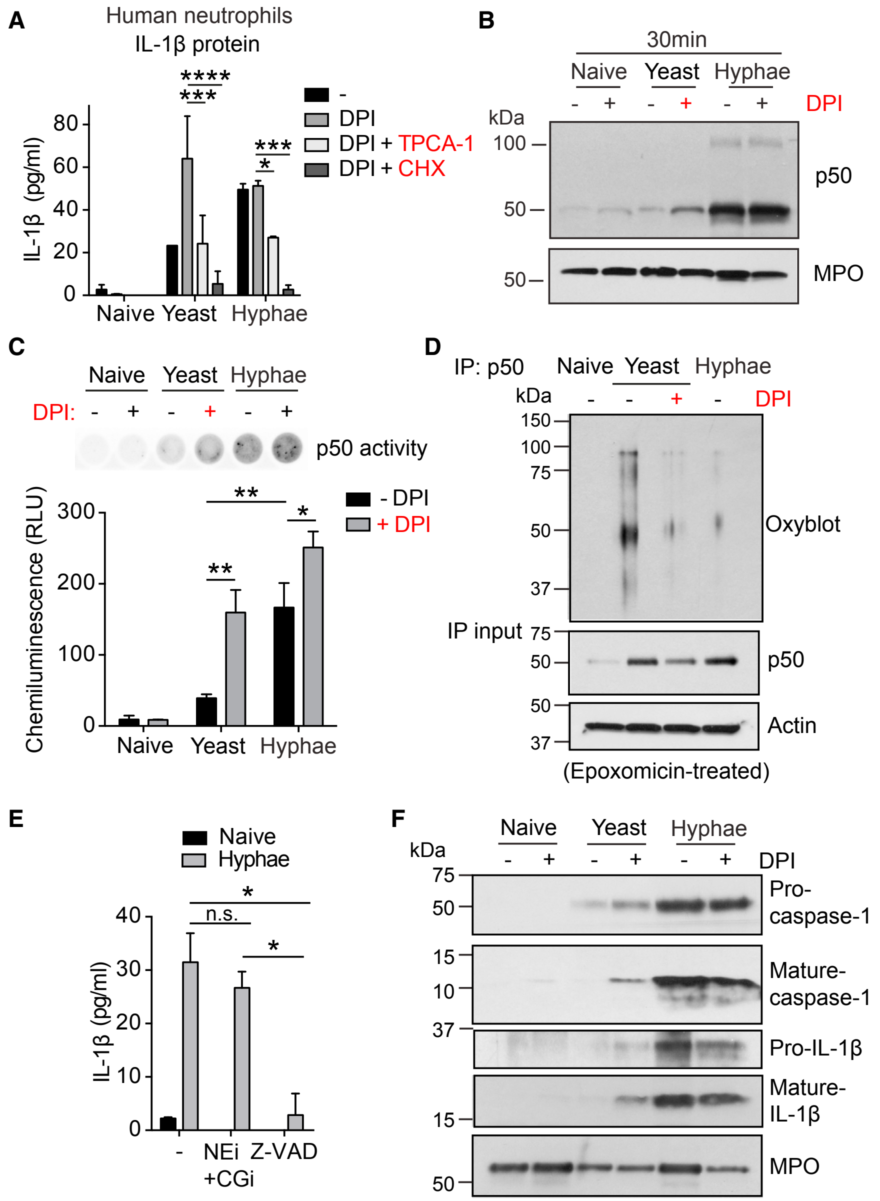

Figure 6. ROS Localization Regulates NF-κB in a Microbe-Size-Dependent Manner

(A) IL-1β release in response to yeast or hyphae by human neutrophils treated with NOX2 inhibitor DPI or in combination with IKK1 and 2 inhibitor TPCA-1 or translation inhibitor cycloheximide (CHX). Data are means ± SD of technical duplicates. Representative of three independent experiments.

(B) Immunoblot analysis of NF-κB p50 and myeloperoxidase (MPO) in human neutrophil lysates 30 min after exposure to yeast or hyphae.

(C) p50 activity in human neutrophil lysates 45 min after *C. albicans* exposure in the presence or absence of DPI measured by p50 binding to its consensus DNA sequence in coated 96-well plates. Data are means ± SD of technical duplicates. Representative of three independent experiments.

(D) Carbonylation of p50 immunoprecipitated from lysates of epoxomicin-treated human neutrophils 45 min after exposure to yeast or hyphae. Representative of three independent experiments.

(E) Effect of protease inhibitors on IL-1β release. Human neutrophils untreated or treated with the neutrophil elastase inhibitor GW311616A (NEi), in combination with cathepsin G inhibitor (CGi) or the pan-caspase inhibitor Z-VAD-fmk and exposed to hyphae. Data are means ± SD of technical duplicates. Representative of three independent experiments.

(F) Western immunoblot of caspase-1 and IL-1β maturation in lysates of human neutrophils after 1 hr of exposure to yeast or hyphae untreated or treated with DPI. MPO serves as loading control. Representative of two independent experiments.

Statistics by two-way ANOVA followed by Sidak's multiple comparison post-test. ****$p < 0.0001$, ***$p < 0.001$, **$p < 0.01$, *$p < 0.05$.

was insensitive to ROS suppression as it remained unaffected by DPI in response to hyphae (Figure 6F). In contrast, yeast failed to induce caspase-1 activation and IL-1β maturation but ROS inhibition partially restored mature Caspase-1 p10 and IL-1β p17 to 5% and 36% of the concentrations induced by hyphae. Therefore, both NF-κB signaling and inflammasome activation are sensitive to ROS localization.

DISCUSSION

Our findings demonstrated that neutrophils were the major drivers of microbe-size-dependent IL-1β amplification during pulmonary infection but that alveolar macrophages are likely to recruit the first wave of neutrophils. The selective oxidative regulation of neutrophil-derived cytokines tuned inflammation to microbe size. Neutrophils ingested small microbes and produced ROS intracellularly by assembling the NADPH oxidase on the phagosome membrane. ROS permeated into the cytosol and oxidized p50, promoting its degradation and limiting IL-1β release to attenuate excessive neutrophil recruitment and minimize the risk for immune pathology. In response to large pathogens, the NADPH oxidase assembled on the plasma membrane, pumping ROS in the extracellular space, thus sparing p50 from oxidation. Consequently, active NF-κB amplified IL-1β expression to recruit additional neutrophils and organize them into clusters required to clear large pathogens. Similarly, phagocytosis regulates NET formation by sequestration of neutrophil elastase (Branzk et al., 2014). These findings place phagocytosis as a central regulator of microbe-size-dependent neutrophil responses via different effector mechanisms.

This model counters the hypothesis that "frustrated phagocytosis" augments phagocyte activation by allowing prolonged receptor signaling from the phagocyte cell surface. Instead, receptor signaling in response to microbes of different size is comparable in neutrophils (Branzk et al., 2014), but selective phagocytosis of small microbes suppresses cytokine transcription by

generating ROS intracellularly. Furthermore, β-glucan purified from *C. albicans* hyphae cell walls is more potent than yeast-derived β-glucan (Lowman et al., 2014). Yet, β-glucan is buried under a mannan layer in hyphae which is likely to counter these intrinsic properties (Steele et al., 2005). Given that soluble microbial agonists are likely to generate ROS extracellularly as they are not phagocytized (Goodridge et al., 2011), experiments with these compounds might not take into account the effects of ROS localization. Employing ROS as a signaling intermediate enables the immune system to sense the size in a variety of microbes irrespectively of specific microbial surface attributes.

Neutrophil clustering has also been reported in response to bacterial skin infection with *S. aureus* (Abtin et al., 2014) and sterile focal skin injury (Lämmermann et al., 2013). While we also observed some degree of neutrophil clustering upon infection with small microbes, the response is dramatically upregulated against large pathogens. Important differences exist between pulmonary infection that spreads microbes across the lung and the skin models that generate a challenge at a single location. Bacterial aggregation may also play a role in these responses because coagulates and clumping factor A induce *S. aureus* agglutination in vivo (Malachowa et al., 2016; McAdow et al., 2011). In addition, one can't exclude that skin tissue disruption by growing hyphae, which constitutes a major fungal virulence mechanism, contributes to neutrophil activation in vivo.

Due to the lower amplitude of the ROS burst, macrophages and dendritic cells might be more sensitive to differences in the activation of innate immune receptors. Consistently, experiments with *C. albicans* skin infections show that Langerhans cells (LCs) distinguish yeast from hyphae by detecting β-glucan on the surface of yeast via the c-type lectin receptor Dectin-1 (Kashem et al., 2015). Selective IL-6 expression in response to yeast drives adaptive T helper 17 (Th17) cell responses. However, we detected lower concentrations of IL-17 upon infection with the yeast-locked mutant, reflecting potential differences between innate lymphoid cell and γδ-T cell responses that depend on IL-1β and adaptive Th17 cell polarization. However, neutrophil-derived extracellular ROS might influence neighboring DCs (Huang et al., 2015).

Recent work in apoptosis-associated speck-like protein containing a carboxy-terminal CARD (ASC)-deficient mice suggested that IL-1β plays no role in neutrophil recruitment and clearance of *A. fumigatus*, implicating monocyte-derived IL-1α instead (Caffrey et al., 2015). The study employed 500-fold higher infectious doses, which is likely to mask the requirement for neutrophil organization by saturating the lung with neutrophils as a result of an overwhelming fungal burden.

The sensitivity to the location of ROS may regulate many transcripts. ROS suppresses cytokine production by activating ataxia-telangiectasia mutated (ATM) (Harbort et al., 2015) to inhibit the p38 MAP kinase, which triggers the alternative NF-κB pathway. Patients with mutations in ATM suffer from chronic inflammation and it will be interesting to investigate whether they fail to respond selectively to microbe size. Moreover, neutrophils produce IL-17 upon *A. fumigatus* infection. IL-17 expression is unaltered in NOX2-deficient neutrophils, which might be consistent with RORγt being insensitive to ROS (Taylor et al., 2014).

Defending against microbes of different size relies on distinct innate inflammatory programs that may be relevant in helminth infection (Sutherland et al., 2014). This model explains the inflammatory pathology associated with CGD and potentially other conditions. Without ROS to detect microbe size, the innate immune system of CGD patients responds to every infection by erroneously mounting a hyper-inflammatory program designed to clear large pathogens.

EXPERIMENTAL PROCEDURES

Mice

All experiments were conducted with age-matched, 8- to 12-week-old male or female C57BL/6J, gp91phox (*Cybb*)-deficient (B6.129S6-*Cybb*^{tm1Din}/J), and MPO-deficient (B6.129X1-*Mpo*^{tm1Lus}/J) mice, according to local guidelines and UK Home Office regulations under the Animals Scientific Procedures Act 1986 (ASPA).

Microbes

Wild-type *C. albicans* (clinical isolate SC5314) and yeast-locked *C. albicans* *Δhgc1* (Zheng et al., 2004) were cultured overnight at 37°C in yeast extract peptone dextrose (YEPD) medium. YL *C. albicans* was subcultured in YEPD medium. WT *C. albicans* was subcultured for 4 hr either in RPMI medium to induce hyphal growth or in YEPD medium for in vivo infection experiments. Subcultures were centrifuged and resuspended in phosphate-buffered saline (PBS) immediately prior to infection. Fragmented hyphae approximately 5 μm long were generated by heat inactivation for 30 min at 90°C and shearing in an EmulsiFlex-C5 high-pressure homogenizer (Avestin). *S. pneumoniae* TIGR4 (Serotype 2) was cultured in brain-heart infusion broth at 37°C overnight, subcultured to an optical density of 0.4, and resuspended in PBS. *S. cerevisiae cbk1*Δ mutant (T74) (Racki et al., 2000) and control strains (PT31-52A) were cultured overnight at 30°C in YEPD medium. *M. bovis* BCG single cells were grown at 37°C, with shaking at 100 rpm, to an absorbance of 0.8 at 600 nm in Middlebrook 7H9 medium supplemented with 10% oleic acid, albumin, dextrose, and catalase (OADC supplement), 0.05% Tween-80, 0.4% glycerol, and 50 μg/mL hygromycin. Bacterial cultures were centrifuged and supernatants were repeatedly passed through a syringe for the removal of large aggregates. Large aggregates were grown without shaking for 3 days and centrifuged through a 50% Percoll bed containing 1× PBS to separate from single bacteria. Wild-type *A. fumigatus* (clinical isolate 13073) was grown on sabourad dextrose agar plates. When plates were fully covered, conidia were harvested by adding 0.1% Tween-20 in PBS and scraping them off. To obtain swollen conidia, *A. fumigatus* was subcultured for 4 hr at 37°C in RPMI medium. Swollen conidia were washed, counted, and resuspended in PBS for in vivo infection experiments.

Pulmonary Infection Studies

To study the effects of blocking antibodies on mouse weight and fungal load at 48 hr after infection, mice were infected intratracheally with 1×10^4 *C. albicans*. For analysis of cytokines in BAL, expression in lung homogenates, and immunofluorescence microscopy-based neutrophil clustering, 24 hr after infection, 1×10^5 *C. albicans*, 1×10^5 *S. pneumonia*, or 1×10^6 *A. fumigatus* were employed. To analyze the effect of mixed *C. albicans* infection, a suspension of 5×10^4 wild-type and 5×10^4 yeast-locked *C. albicans* in PBS were administered intratracheally.

Cell Culture

Peripheral blood was collected from healthy adult volunteers according to protocols approved by the ethics board of the Francis Crick Institute. Human neutrophils were freshly isolated over Histopaque 1119 gradient followed by a discontinuous Percoll gradient (Aga et al., 2002) if indicated followed by negative selection with the EasySep human neutrophil enrichment kit (Stemcell). Mouse neutrophils were isolated from *C. albicans*-infected lung cell suspensions or from bone marrow using the EasySep mouse neutrophil enrichment kit (Stemcell).

Mouse and human neutrophils were plated in Hank's balanced-salt solution plus Ca^{2+} and Mg^{2+} supplemented with 3% human plasma or 10% fetal calf serum for mouse neutrophils, respectively. Cells were pretreated for 1 hr with 10 μM NADPH oxidase inhibitor diphenyleneiodonium (DPI, Sigma), 10 μM Neutrophil elastase inhibitor NEi (GW311616A, Sigma), 5 μM Cathepsin G inhibitor CGi (Millipore), 20 μM pan-caspase inhibitor (Z-VAD-fmk, Calbiochem), 5 μM IKK2 inhibitor 2-[(Aminocarbonyl)amino]-5-(4-fluorophenyl)-3-thiophenecarboxamide (TPCA-1, Biovision), 50 μg/mL translation inhibitor cycloheximide (CHX, Sigma), or 200 nM proteasome inhibitor Epoxomicin (Calbiochem). Neutrophils were stimulated with *C. albicans* yeast-locked, preformed hyphae or fragmented hyphae, as well as *S. cerevisiae* or *M. bovis* at a MOI 10 unless otherwise stated. For bead inhibition studies, neutrophils were pre-incubated with 4 μm polystyrene-coated flow cytometry beads (Kisker Biotech) that were opsonized for 1 hr in 100% human plasma and washed 3× in PBS, 30 min prior to stimulation with *C. albicans* preformed hyphae. For stimulation with β-glucan-coated beads, 4 μm (Kisker Biotech) and 30 μm (Sigma) polystyrene-based beads were incubated for 1 hr with 2 mg/mL β-glucan in PBS/EDTA and subsequently washed 3× in PBS/EDTA to remove soluble β-glucan.

SUPPLEMENTAL INFORMATION

Supplemental Information includes six figures, one table, and Supplemental Experimental Procedures and can be found with this article online at http://dx.doi.org/10.1016/j.immuni.2017.02.013.

AUTHOR CONTRIBUTIONS

A.W. designed and performed experiments, analyzed data, and wrote the manuscript with V.P., who designed and supervised the project. N.B. and T.-D.T. performed live imaging and fungal growth inhibition assays. S.H. and M.G. prepared BCG samples. T.-D.T., Q.W., and S.R. contributed to experiments.

ACKNOWLEDGMENTS

We thank B. Stockinger, G. Kassiotis, and D. Anastasiou for comments, H. Aegerter for help with BCG *S. pneumoniae*, and P. Thorpe and E. Herrero-Serrano for *S. cerevisiae* strains. This work was supported by the Francis Crick Institute which receives its core funding from the UK Medical Research Council (MC_UP_1202/13, FC001129, MC_UP_1202/11, FC001092), Cancer Research UK (FC001129, FC001092), and the Wellcome Trust (FC001129, FC001092).

Received: September 27, 2016
Revised: December 22, 2016
Accepted: January 13, 2017
Published: March 14, 2017

REFERENCES

Abtin, A., Jain, R., Mitchell, A.J., Roediger, B., Brzoska, A.J., Tikoo, S., Cheng, Q., Ng, L.G., Cavanagh, L.L., von Andrian, U.H., et al. (2014). Perivascular macrophages mediate neutrophil recruitment during bacterial skin infection. Nat. Immunol. *15*, 45–53.

Aga, E., Katschinski, D.M., van Zandbergen, G., Laufs, H., Hansen, B., Müller, K., Solbach, W., and Laskay, T. (2002). Inhibition of the spontaneous apoptosis of neutrophil granulocytes by the intracellular parasite *Leishmania major*. J. Immunol. *169*, 898–905.

Amulic, B., Cazalet, C., Hayes, G.L., Metzler, K.D., and Zychlinsky, A. (2012). Neutrophil Function: From Mechanisms to Disease. In Annual Review of Immunology, *Volume 30*, pp. 459–489.

Bagaitkar, J., Pech, N.K., Ivanov, S., Austin, A., Zeng, M.Y., Pallat, S., Huang, G., Randolph, G.J., and Dinauer, M.C. (2015). NADPH oxidase controls neutrophilic response to sterile inflammation in mice by regulating the IL-1α/G-CSF axis. Blood *126*, 2724–2733.

Bianchi, M., Niemiec, M.J., Siler, U., Urban, C.F., and Reichenbach, J. (2011). Restoration of anti-Aspergillus defense by neutrophil extracellular traps in human chronic granulomatous disease after gene therapy is calprotectin-dependent. J. Allergy Clin. Immunol. *127*, 1243–1252.e7.

Branzk, N., Lubojemska, A., Hardison, S.E., Wang, Q., Gutierrez, M.G., Brown, G.D., and Papayannopoulos, V. (2014). Neutrophils sense microbe size and selectively release neutrophil extracellular traps in response to large pathogens. Nat. Immunol. *15*, 1017–1025.

Brown, G.D., Denning, D.W., Gow, N.A., Levitz, S.M., Netea, M.G., and White, T.C. (2012). Hidden killers: human fungal infections. Sci. Transl. Med. *4*, 165rv13.

Caffrey, A.K., Lehmann, M.M., Zickovich, J.M., Espinosa, V., Shepardson, K.M., Watschke, C.P., Hilmer, K.M., Thammahong, A., Barker, B.M., Rivera, A., et al. (2015). IL-1α signaling is critical for leukocyte recruitment after pulmonary *Aspergillus fumigatus* challenge. PLoS Pathog. *11*, e1004625.

Chen, K.W., Groß, C.J., Sotomayor, F.V., Stacey, K.J., Tschopp, J., Sweet, M.J., and Schroder, K. (2014). The neutrophil NLRC4 inflammasome selectively promotes IL-1β maturation without pyroptosis during acute *Salmonella* challenge. Cell Rep. *8*, 570–582.

Cho, J.S., Guo, Y., Ramos, R.I., Hebroni, F., Plaisier, S.B., Xuan, C., Granick, J.L., Matsushima, H., Takashima, A., Iwakura, Y., et al. (2012). Neutrophil-derived IL-1β is sufficient for abscess formation in immunity against *Staphylococcus aureus* in mice. PLoS Pathog. *8*, e1003047.

Devalon, M.L., Elliott, G.R., and Regelmann, W.E. (1987). Oxidative response of human neutrophils, monocytes, and alveolar macrophages induced by unopsonized surface-adherent *Staphylococcus aureus*. Infect. Immun. *55*, 2398–2403.

Gantner, B.N., Simmons, R.M., and Underhill, D.M. (2005). Dectin-1 mediates macrophage recognition of *Candida albicans* yeast but not filaments. EMBO J. *24*, 1277–1286.

Goodridge, H.S., Reyes, C.N., Becker, C.A., Katsumoto, T.R., Ma, J., Wolf, A.J., Bose, N., Chan, A.S., Magee, A.S., Danielson, M.E., et al. (2011). Activation of the innate immune receptor Dectin-1 upon formation of a 'phagocytic synapse'. Nature *472*, 471–475.

Han, W., Li, H., Cai, J., Gleaves, L.A., Polosukhin, V.V., Segal, B.H., Yull, F.E., and Blackwell, T.S. (2013). NADPH oxidase limits lipopolysaccharide-induced lung inflammation and injury in mice through reduction-oxidation regulation of NF-κB activity. J. Immunol. *190*, 4786–4794.

Harbort, C.J., Soeiro-Pereira, P.V., von Bernuth, H., Kaindl, A.M., Costa-Carvalho, B.T., Condino-Neto, A., Reichenbach, J., Roesler, J., Zychlinsky, A., and Amulic, B. (2015). Neutrophil oxidative burst activates ATM to regulate cytokine production and apoptosis. Blood *126*, 2842–2851.

Huang, X., Li, J., Dorta-Estremera, S., Di Domizio, J., Anthony, S.M., Watowich, S.S., Popkin, D., Liu, Z., Brohawn, P., Yao, Y., et al. (2015). Neutrophils regulate humoral autoimmunity by restricting interferon-γ production via the generation of reactive oxygen species. Cell Rep. *12*, 1120–1132.

Joly, S., Ma, N., Sadler, J.J., Soll, D.R., Cassel, S.L., and Sutterwala, F.S. (2009). Cutting edge: *Candida albicans* hyphae formation triggers activation of the Nlrp3 inflammasome. J. Immunol. *183*, 3578–3581.

Karmakar, M., Katsnelson, M., Malak, H.A., Greene, N.G., Howell, S.J., Hise, A.G., Camilli, A., Kadioglu, A., Dubyak, G.R., and Pearlman, E. (2015). Neutrophil IL-1β processing induced by pneumolysin is mediated by the NLRP3/ASC inflammasome and caspase-1 activation and is dependent on K+ efflux. J. Immunol. *194*, 1763–1775.

Kashem, S.W., Igyártó, B.Z., Gerami-Nejad, M., Kumamoto, Y., Mohammed, J., Jarrett, E., Drummond, R.A., Zurawski, S.M., Zurawski, G., Berman, J., et al. (2015). *Candida albicans* morphology and dendritic cell subsets determine T helper cell differentiation. Immunity *42*, 356–366.

Kolaczkowska, E., Jenne, C.N., Surewaard, B.G., Thanabalasuriar, A., Lee, W.Y., Sanz, M.J., Mowen, K., Opdenakker, G., and Kubes, P. (2015). Molecular mechanisms of NET formation and degradation revealed by intravital imaging in the liver vasculature. Nat. Commun. *6*, 6673.

Lämmermann, T., Afonso, P.V., Angermann, B.R., Wang, J.M., Kastenmüller, W., Parent, C.A., and Germain, R.N. (2013). Neutrophil swarms require LTB4 and integrins at sites of cell death in vivo. Nature *498*, 371–375.

Latz, E., Xiao, T.S., and Stutz, A. (2013). Activation and regulation of the inflammasomes. Nat. Rev. Immunol. *13*, 397–411.

Lowman, D.W., Greene, R.R., Bearden, D.W., Kruppa, M.D., Pottier, M., Monteiro, M.A., Soldatov, D.V., Ensley, H.E., Cheng, S.C., Netea, M.G., and Williams, D.L. (2014). Novel structural features in *Candida albicans* hyphal glucan provide a basis for differential innate immune recognition of hyphae versus yeast. J. Biol. Chem. *289*, 3432–3443.

Malachowa, N., Kobayashi, S.D., Porter, A.R., Braughton, K.R., Scott, D.P., Gardner, D.J., Missiakas, D.M., Schneewind, O., and DeLeo, F.R. (2016). Contribution of *Staphylococcus aureus* coagulases and clumping factor A to abscess formation in a rabbit model of skin and soft tissue Infection. PLoS ONE *11*, e0158293.

McAdow, M., Kim, H.K., Dedent, A.C., Hendrickx, A.P., Schneewind, O., and Missiakas, D.M. (2011). Preventing *Staphylococcus aureus* sepsis through the inhibition of its agglutination in blood. PLoS Pathog. *7*, e1002307.

Medzhitov, R. (2008). Origin and physiological roles of inflammation. Nature *454*, 428–435.

Meissner, F., Molawi, K., and Zychlinsky, A. (2008). Superoxide dismutase 1 regulates caspase-1 and endotoxic shock. Nat. Immunol. *9*, 866–872.

Meissner, F., Seger, R.A., Moshous, D., Fischer, A., Reichenbach, J., and Zychlinsky, A. (2010). Inflammasome activation in NADPH oxidase defective mononuclear phagocytes from patients with chronic granulomatous disease. Blood *116*, 1570–1573.

Metzler, K.D., Fuchs, T.A., Nauseef, W.M., Reumaux, D., Roesler, J., Schulze, I., Wahn, V., Papayannopoulos, V., and Zychlinsky, A. (2011). Myeloperoxidase is required for neutrophil extracellular trap formation: implications for innate immunity. Blood *117*, 953–959.

Metzler, K.D., Goosmann, C., Lubojemska, A., Zychlinsky, A., and Papayannopoulos, V. (2014). A myeloperoxidase-containing complex regulates neutrophil elastase release and actin dynamics during NETosis. Cell Rep. *8*, 883–896.

Miller, L.S., O'Connell, R.M., Gutierrez, M.A., Pietras, E.M., Shahangian, A., Gross, C.E., Thirumala, A., Cheung, A.L., Cheng, G., and Modlin, R.L. (2006). MyD88 mediates neutrophil recruitment initiated by IL-1R but not TLR2 activation in immunity against *Staphylococcus aureus*. Immunity *24*, 79–91.

Miller, L.S., Pietras, E.M., Uricchio, L.H., Hirano, K., Rao, S., Lin, H., O'Connell, R.M., Iwakura, Y., Cheung, A.L., Cheng, G., and Modlin, R.L. (2007). Inflammasome-mediated production of IL-1beta is required for neutrophil recruitment against *Staphylococcus aureus* in vivo. J. Immunol. *179*, 6933–6942.

Morgenstern, D.E., Gifford, M.A., Li, L.L., Doerschuk, C.M., and Dinauer, M.C. (1997). Absence of respiratory burst in X-linked chronic granulomatous disease mice leads to abnormalities in both host defense and inflammatory response to *Aspergillus fumigatus*. J. Exp. Med. *185*, 207–218.

Murphy, M.P., Holmgren, A., Larsson, N.G., Halliwell, B., Chang, C.J., Kalyanaraman, B., Rhee, S.G., Thornalley, P.J., Partridge, L., Gems, D., et al. (2011). Unraveling the biological roles of reactive oxygen species. Cell Metab. *13*, 361–366.

Nathan, C., and Shiloh, M.U. (2000). Reactive oxygen and nitrogen intermediates in the relationship between mammalian hosts and microbial pathogens. Proc. Natl. Acad. Sci. USA *97*, 8841–8848.

Netea, M.G., Brown, G.D., Kullberg, B.J., and Gow, N.A. (2008). An integrated model of the recognition of *Candida albicans* by the innate immune system. Nat. Rev. Microbiol. *6*, 67–78.

Papayannopoulos, V., Metzler, K.D., Hakkim, A., and Zychlinsky, A. (2010). Neutrophil elastase and myeloperoxidase regulate the formation of neutrophil extracellular traps. J. Cell Biol. *191*, 677–691.

Park, H., Li, Z., Yang, X.O., Chang, S.H., Nurieva, R., Wang, Y.H., Wang, Y., Hood, L., Zhu, Z., Tian, Q., and Dong, C. (2005). A distinct lineage of CD4 T cells regulates tissue inflammation by producing interleukin 17. Nat. Immunol. *6*, 1133–1141.

Pineda-Molina, E., Klatt, P., Vázquez, J., Marina, A., García de Lacoba, M., Pérez-Sala, D., and Lamas, S. (2001). Glutathionylation of the p50 subunit of NF-kappaB: a mechanism for redox-induced inhibition of DNA binding. Biochemistry *40*, 14134–14142.

Plato, A., Hardison, S.E., and Brown, G.D. (2015). Pattern recognition receptors in antifungal immunity. Semin. Immunopathol. *37*, 97–106.

Pollock, J.D., Williams, D.A., Gifford, M.A., Li, L.L., Du, X., Fisherman, J., Orkin, S.H., Doerschuk, C.M., and Dinauer, M.C. (1995). Mouse model of X-linked chronic granulomatous disease, an inherited defect in phagocyte superoxide production. Nat. Genet. *9*, 202–209.

Racki, W.J., Bécam, A.M., Nasr, F., and Herbert, C.J. (2000). Cbk1p, a protein similar to the human myotonic dystrophy kinase, is essential for normal morphogenesis in *Saccharomyces cerevisiae*. EMBO J. *19*, 4524–4532.

Rieber, N., Hector, A., Kuijpers, T., Roos, D., and Hartl, D. (2012). Current concepts of hyperinflammation in chronic granulomatous disease. Clin. Dev. Immunol. *2012*, 252460.

Saïd-Sadier, N., Padilla, E., Langsley, G., and Ojcius, D.M. (2010). *Aspergillus fumigatus* stimulates the NLRP3 inflammasome through a pathway requiring ROS production and the Syk tyrosine kinase. PLoS ONE *5*, e10008.

Silva, M.T. (2010). When two is better than one: macrophages and neutrophils work in concert in innate immunity as complementary and cooperative partners of a myeloid phagocyte system. J. Leukoc. Biol. *87*, 93–106.

Steele, C., Rapaka, R.R., Metz, A., Pop, S.M., Williams, D.L., Gordon, S., Kolls, J.K., and Brown, G.D. (2005). The beta-glucan receptor dectin-1 recognizes specific morphologies of *Aspergillus fumigatus*. PLoS Pathog. *1*, e42.

Sutherland, T.E., Logan, N., Rückerl, D., Humbles, A.A., Allan, S.M., Papayannopoulos, V., Stockinger, B., Maizels, R.M., and Allen, J.E. (2014). Chitinase-like proteins promote IL-17-mediated neutrophilia in a tradeoff between nematode killing and host damage. Nat. Immunol. *15*, 1116–1125.

Taylor, P.R., Roy, S., Leal, S.M., Jr., Sun, Y., Howell, S.J., Cobb, B.A., Li, X., and Pearlman, E. (2014). Activation of neutrophils by autocrine IL-17A-IL-17RC interactions during fungal infection is regulated by IL-6, IL-23, RORγt and dectin-2. Nat. Immunol. *15*, 143–151.

Toledano, M.B., and Leonard, W.J. (1991). Modulation of transcription factor NF-kappa B binding activity by oxidation-reduction in vitro. Proc. Natl. Acad. Sci. USA *88*, 4328–4332.

Ueyama, T., Tatsuno, T., Kawasaki, T., Tsujibe, S., Shirai, Y., Sumimoto, H., Leto, T.L., and Saito, N. (2007). A regulated adaptor function of p40phox: distinct p67phox membrane targeting by p40phox and by p47phox. Mol. Biol. Cell *18*, 441–454.

van der Graaf, C.A., Netea, M.G., Verschueren, I., van der Meer, J.W., and Kullberg, B.J. (2005). Differential cytokine production and Toll-like receptor signaling pathways by *Candida albicans* blastoconidia and hyphae. Infect. Immun. *73*, 7458–7464.

Warnatsch, A., Ioannou, M., Wang, Q., and Papayannopoulos, V. (2015). Inflammation. Neutrophil extracellular traps license macrophages for cytokine production in atherosclerosis. Science *349*, 316–320.

Zheng, X., Wang, Y., and Wang, Y. (2004). Hgc1, a novel hypha-specific G1 cyclin-related protein regulates *Candida albicans* hyphal morphogenesis. EMBO J. *23*, 1845–1856.

Immunity, Volume *46*

Supplemental Information

Reactive Oxygen Species Localization Programs Inflammation to Clear Microbes of Different Size

Annika Warnatsch, Theodora-Dorita Tsourouktsoglou, Nora Branzk, Qian Wang, Susanna Reincke, Susanne Herbst, Maximiliano Gutierrez, and Venizelos Papayannopoulos

Figure S1

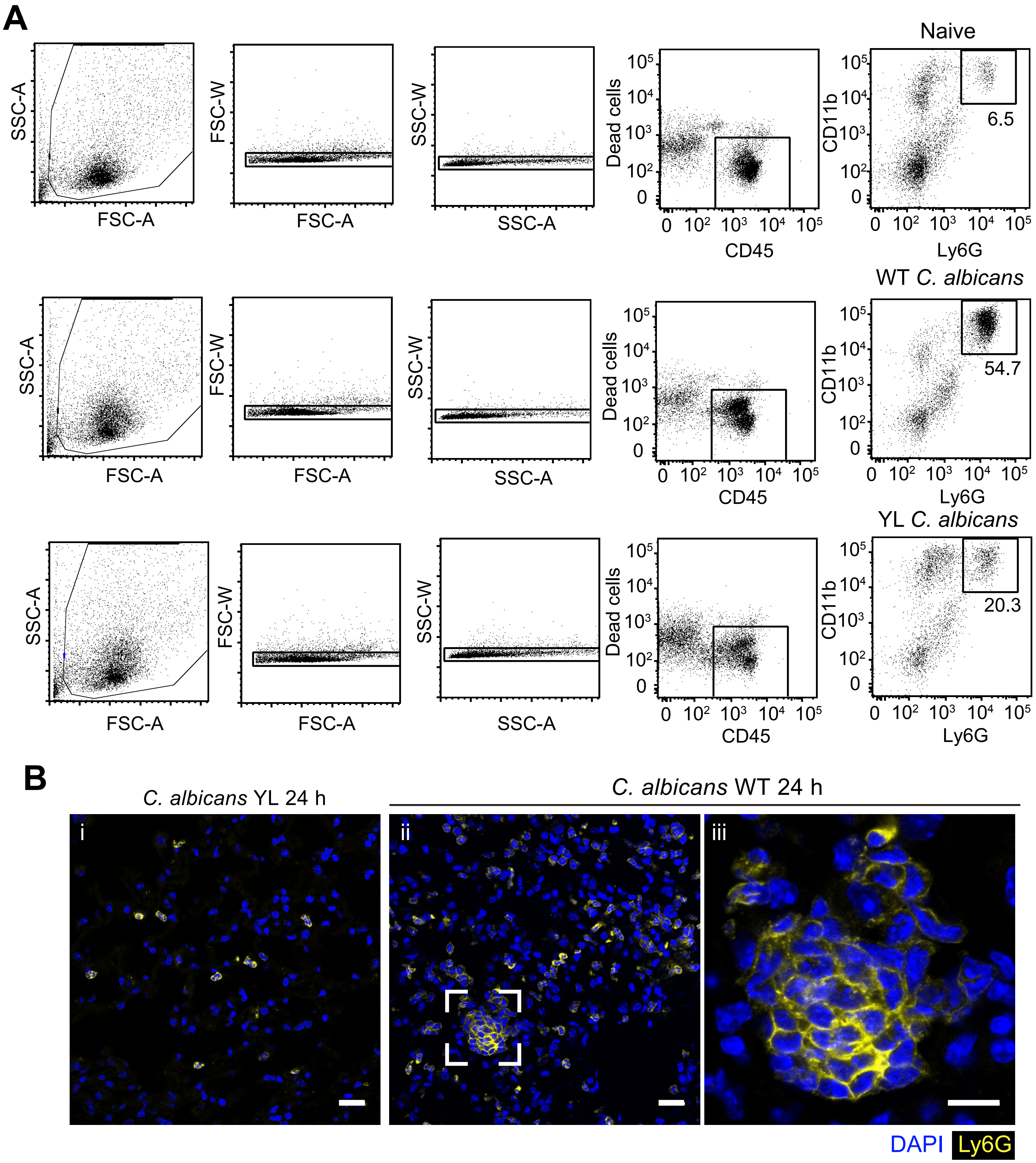

Figure S1, related to Figure 1H. Gating strategy for neutrophil count in infected lungs.

(A) FACS gating strategy for counting neutrophils in whole lungs of mice infected with yeast-locked (YL) or WT *C. albicans*. Neutrophils were gated as live, CD45+, CD11b+, Ly6G+ cells.
(B) Dispersed and clustered neutrophils 24 hrs post infection with YL or WT *C. albicans*. Lung sections stained for Ly6G (yellow), and DAPI (blue). Scale bars: 20µm (i and ii), 10µm (iii).

Figure S2

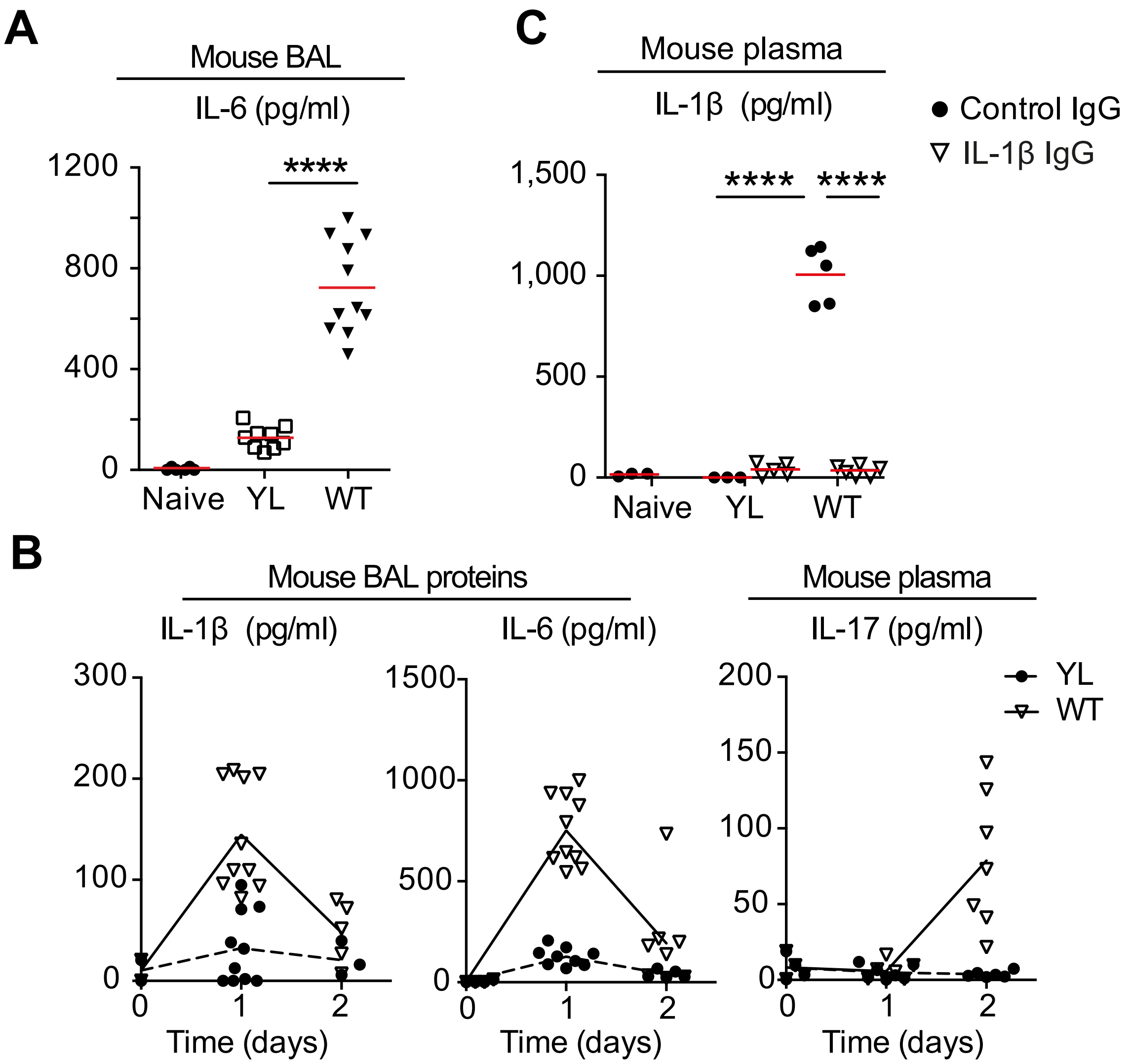

Figure S2, related to Figure 2. Neutrophil clustering and cytokine analysis in mice infected with *C. albicans*.

(A) Differential induction of IL-6 in the BAL of mice infected with 10^5 yeast-locked (YL) or WT *C. albicans*, 24 hrs post-infection. Each point represents one animal. Statistics by 2-way ANOVA and Tukey's post-test. **** $p < 0.0001$.
(B) IL-1β and IL-6 protein in the BAL and IL-17 protein in the plasma of mice infected with 10^5 yeast-locked (YL) or WT *C. albicans* 1 and 2 days post infection. Each point represents one animal.
(C) Upon pulmonary infection with yeast-locked (YL) or WT *C. albicans*, mice treated with an anti-IL-1β antibody do not bear significant amounts of circulating IL-1β in their blood plasma 24 hrs post-infection as compared to isotype control treated mice. Cytokine protein was detected by ELISA. Each point represents one animal. Statistics by 2-way ANOVA and Tukey's post-test. **** $p < 0.0001$.

Figure S3

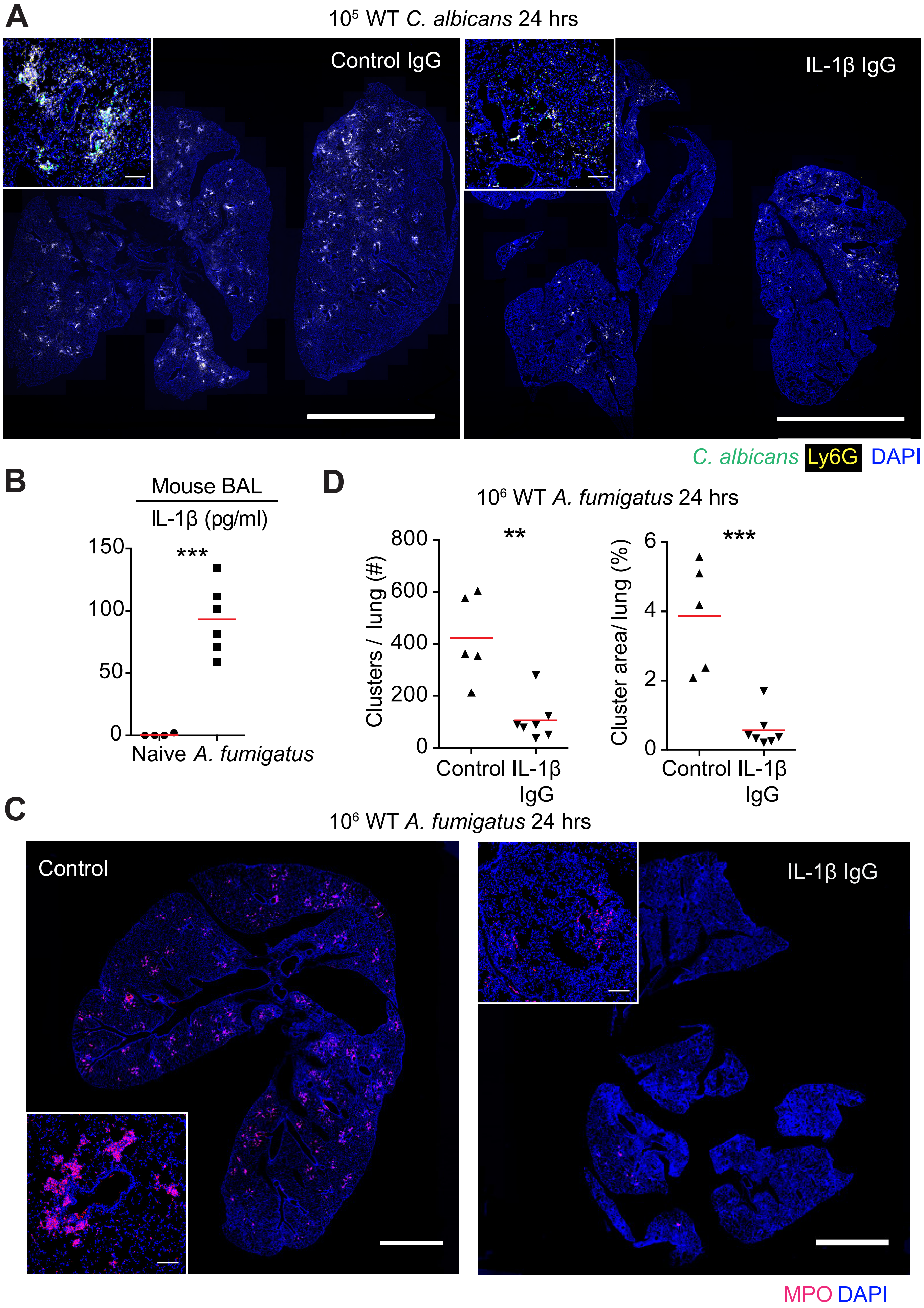

A
10⁵ WT C. albicans 24 hrs
Control IgG
IL-1β IgG
C. albicans Ly6G DAPI
B
Mouse BAL
IL-1β (pg/ml)
150
100
50
0
***
Naive A. fumigatus
D
10⁶ WT A. fumigatus 24 hrs
Clusters / lung (#)
800
600
400
200
0
**
Control IL-1β IgG
Cluster area/ lung (%)
6
4
2
0
***
Control IL-1β IgG
C
10⁶ WT A. fumigatus 24 hrs
Control
IL-1β IgG
MPO DAPI

Figure S3, related to Figure 2. Neutrophil responses to *C. albicans* and *A. fumigatus* pulmonary infection.

(A) Immunofluorescence confocal micrographs of lung sections stained for DNA (DAPI, blue), neutrophils (Ly6G, yellow) and *C. albicans* (green) from WT mice treated with an isotype control or an antibody blocking IL-1β, 24 hrs post infection and infected with 10^5 WT *C. albicans* yeast, which switches to hyphal growth *in vivo*. Scale bars: (A) 5 mm, (B) 2 mm, (a and b) inserts 100 μm.
(B) IL-1β production in the BAL of WT mice infected with 10^6 *A. fumigatus* swollen conidia 24 hrs post-infection measured by ELISA. Each point represents one animal. Statistics by two-tailed student's t-test.
(C) As in (A) but infected with 10^6 swollen *A. fumigatus* conidia, stained for DNA (DAPI, blue), neutrophils (MPO, red). Scale bars: 2 mm, inserts 100 μm.
(D) Number of neutrophil clusters and average cluster size per lung section in (C) for mice infected with 10^6 swollen *A. fumigatus* conidia. Each point represents one animal. Statistics by two-tailed student's t-test. *** $p < 0.001$, ** $p < 0.01$.

Figure S4

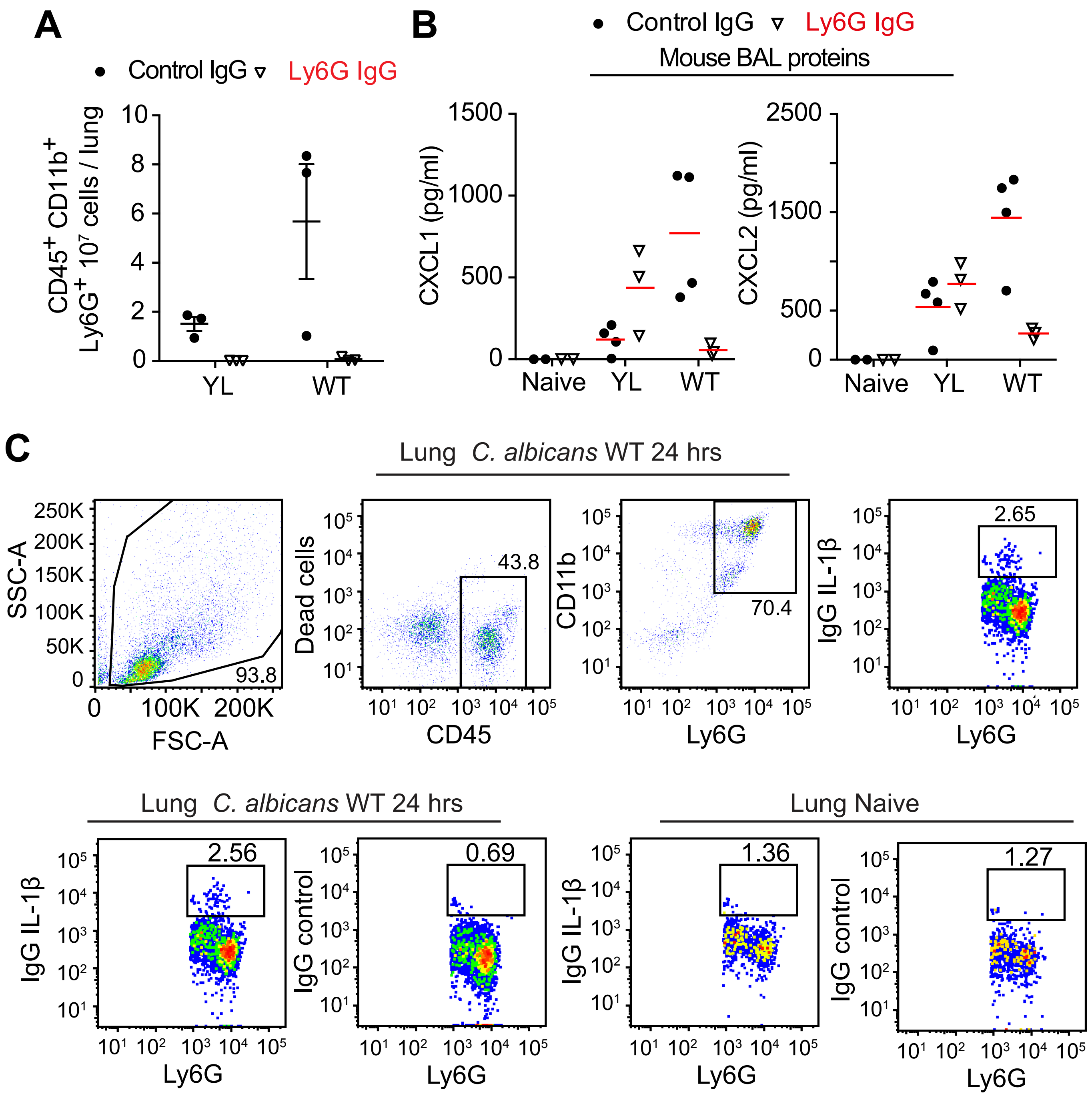

Figure S4, related to Figure 3. Effect of neutrophil depletion on pulmonary neutrophils and neutrophil chemokines.

(A) Effectiveness of neutrophil depletion in lungs of mice infected with yeast-locked (YL) or WT *C. albicans* and pretreated with anti-Ly6G antibody or an isotype control 24 hrs post-infection. Each point represents one animal.
(B) Effect of neutrophil depletion on the neutrophil chemokines CXCL1 and CXCL2 24 hrs post-infection, in the BAL of WT mice infected with yeast-locked (YL) or WT *C. albicans* and pre-treated with anti-Ly6G antibody or an isotype control. Each point represents one animal.
(C) FACS gating strategy for counting IL-1β+ lung neutrophils purified from the lungs of mice either uninfected or infected with WT *C. albicans*.

Figure S5

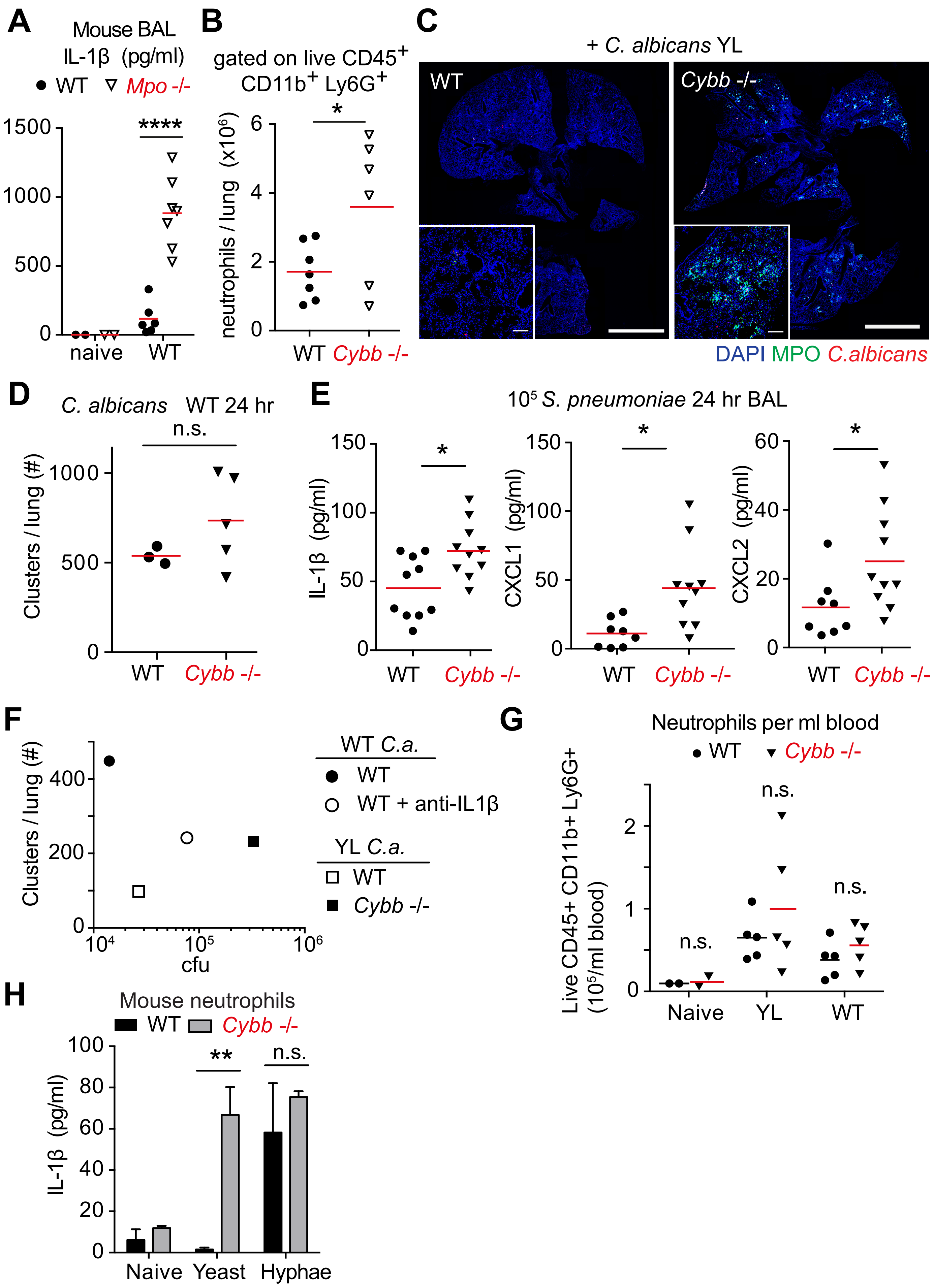

A
Mouse BAL
IL-1β (pg/ml)
WT
Mpo -/-
****
1500
1000
500
0
naive
WT
B
gated on live CD45+ CD11b+ Ly6G+
neutrophils / lung (x10^6)
*
6
4
2
0
WT
Cybb -/-
C
+ C. albicans YL
WT
Cybb -/-
DAPI MPO C.albicans
D
C. albicans WT 24 hr
n.s.
Clusters / lung (#)
1000
500
0
WT
Cybb -/-
E
10^5 S. pneumoniae 24 hr BAL
IL-1β (pg/ml)
CXCL1 (pg/ml)
CXCL2 (pg/ml)
*
150
100
50
0
60
40
20
WT
Cybb -/-
F
Clusters / lung (#)
400
200
0
10^4
10^5
10^6
cfu
WT C.a.
WT
WT + anti-IL1β
YL C.a.
WT
Cybb -/-
G
Neutrophils per ml blood
WT
Cybb -/-
Live CD45+ CD11b+ Ly6G+ (10^5/ml blood)
n.s.
2
1
0
Naive
YL
WT
H
Mouse neutrophils
WT
Cybb -/-
IL-1β (pg/ml)
**
n.s.
100
80
60
40
20
0
Naive
Yeast
Hyphae

Figure S5, related to Figure 4. Effect of ROS on IL-1β production and neutrophil recruitment.

(A) IL-1β protein in the BAL of MPO-deficient mice infected with WT *C. albicans* 24 hrs post-infection. Each point represents one animal. Statistics by 2-way ANOVA and Sidak's post-test.
(B) Number of neutrophils recruited to the lung of WT and *Cybb* -/- mice analyzed by FACS 24 hrs after infection with yeast-locked (YL) or WT *C. albicans*. Each point represents one animal. Statistics by 2-way ANOVA and Sidak's post-test.
(C) Immunofluorescence micrographs of lung sections of WT and *Cybb* -/- mice infected with YL *C. albicans* 24 hrs post-infection, stained for DNA (DAPI, blue), neutrophils (MPO, green) and *C. albicans* (red). Scale bar: 2.5mm, scale bar of inserts: 100μm.
(D) Number of neutrophil clusters in lungs of WT and *Cybb* -/- mice infected with WT *C. albicans*. Each point represents one animal. Statistics by two-tailed student's t-test.
(E) IL-1β, CXCL1 and CXCL2 protein in the BAL of WT and *Cybb* -/- mice infected with *S. pneumoniae* 24 hrs post infection. Each point represents one animal. Statistics by two-tailed student's t-test.
(F) Number of neutrophil clusters plotted against the corresponding WT or YL *C. albicans* lung burden (CFU) in WT (C57/B6) or *Cybb* -/- mice. IL-1β blocking antibody was added were indicated.
(G) FACS measurement of blood neutrophils in WT versus *Cybb* -/- mice either naive or infected with YL or WT *C. albicans* after 24 hrs. Each point represents one animal. Statistics by 2-way ANOVA and Sidak's post-test.
(H) IL-1β protein production by purified bone-marrow neutrophils from WT or *Cybb* -/- mice exposed to yeast or preformed hyphae. Cytokine production was detected by ELISA. Statistics by 2-way ANOVA and Sidak's post-test.
**** $p < 0.0001$, ** $p < 0.01$, * $p < 0.05$.

Figure S6

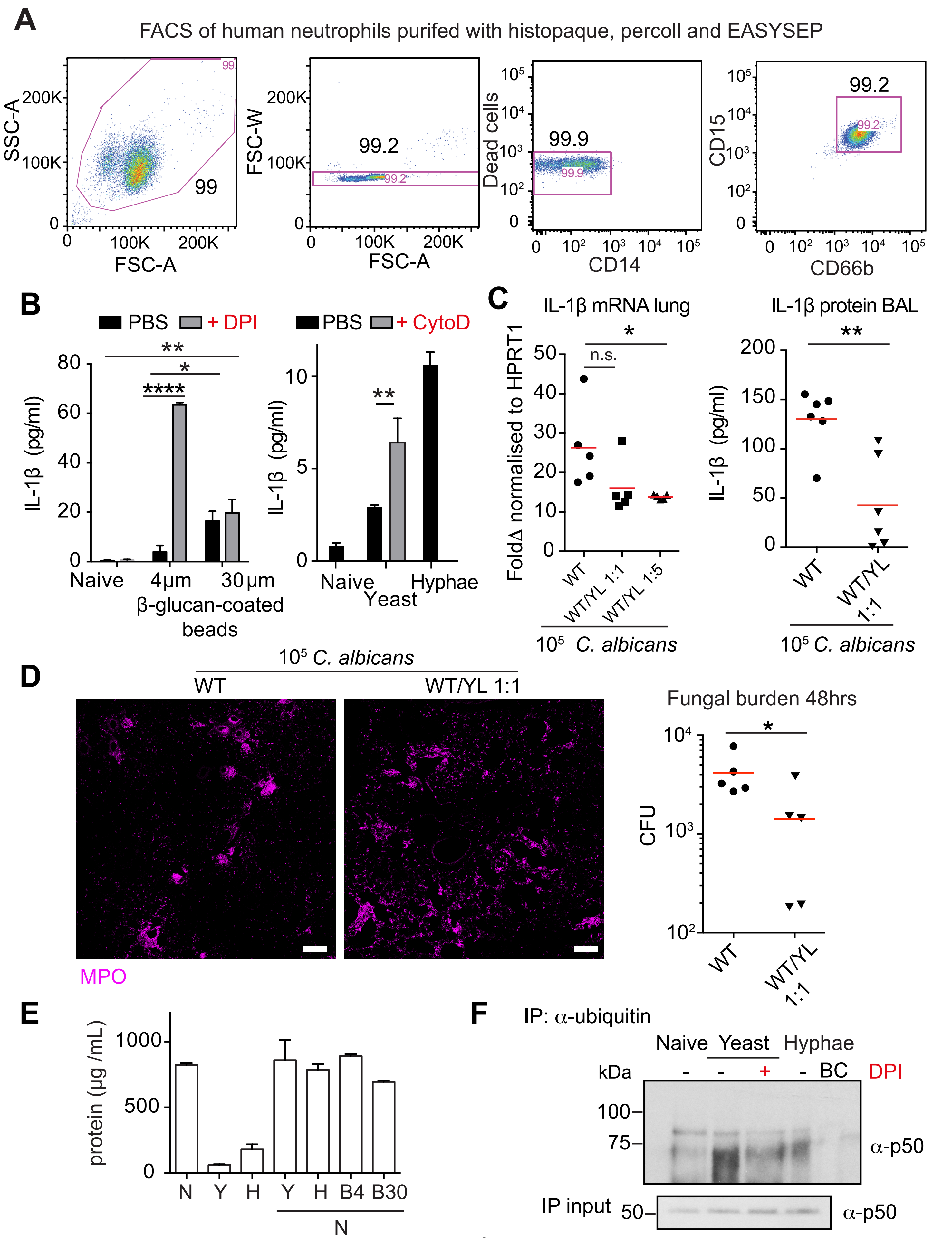
A
FACS of human neutrophils purifed with histopaque, percoll and EASYSEP
SSC-A
FSC-A
FSC-W
Dead cells
CD14
CD15
CD66b
99
99.2
99.9
B
PBS
+ DPI
+ CytoD
IL-1β (pg/ml)
Naive
4µm
30µm
β-glucan-coated beads
Yeast
Hyphae
C
IL-1β mRNA lung
IL-1β protein BAL
FoldΔ normalised to HPRT1
n.s.
WT
WT/YL 1:1
WT/YL 1:5
10⁵ C. albicans
D
WT
WT/YL 1:1
MPO
Fungal burden 48hrs
CFU
E
protein (µg /mL)
N
Y
H
B4
B30
F
IP: α-ubiquitin
Naive
Yeast
Hyphae
kDa
BC
DPI
α-p50
IP input

Figure S6, related to Figures 4, 5 and 6. Effect of microbe size on immune defense and neutrophil signalling.

(A) FACS gating strategy for counting neutrophils purified by Histopaque and Percoll followed by EASYSEP negative selection affinity purification. Neutrophils were gated as live, $CD14^{-}$, $CD15^{+}$, $CD66b^{+}$ cells.
(B) IL-1β protein released by either naive neutrophils or neutrophils treated with 4μm or 30μm wide β-glucan-coated beads (left panel) or yeast and hyphae in the absence or presence of cytochalasin D (right panel). Shown is the mean ±SD of technical duplicates. Statistics by two-way ANOVA and Sidak's post-test. Representative of 3 independent experiments.
(C) IL-1β mRNA in lung or protein in BAL of mice infected with the same inoculum of WT *C. albicans* or a combination of WT and YL strains at 1:1 or 1:5 ratios. Each point represents one animal. Statistics by one-way ANOVA and Tukey's post-test.
(D) Neutrophil clustering in the lung sections of mice from (C) at 24 hrs post infection. Neutrophils were stained for MPO (magenta). Scale bars: 100μm. Fungal burden from the same experiment at 48 hrs post infection. Each point represents one animal. Statistics by one-way ANOVA and Tukey's post-test.
(E) Protein content measured by BCA of neutrophils, yeast or hyphae alone, or neutrophils in combination with either yeast, hyphae at 10:1 MOI (conditions employed in oxidation studies), 4μm or 30μm wide β-glucan-coated beads.
(F) Higher molecular weight, ubiquitinated p50 in lysates of epoxomicin-treated human neutrophils 45 min after exposure to yeast or hyphae untreated or treated with DPI. BC bead control (lysate + beads without anti-ubiquitin antibody). Ubiquitinated proteins were immunoprecipitated with an antibody against ubiquitin, analysed by SDS-PAGE electrophoresis and immunoblotted for p50. p50 content in lysates prior to immunoprecipitation shown below.
**** $p < 0.0001$, ** $p < 0.01$, * $p < 0.05$.

Supplemental experimental procedures

Lung microbe load. After 48 hrs lungs and spleens were homogenized in sterile saline and *C. albicans*-infected tissue plated onto sabourad dextrose agar plates, *S. pneumonia*-infected lungs on blood agar plates.

Depletion studies. Neutrophils were depleted by intraperitoneal administration of 150 µg anti-mouse Ly6G (clone 1A8) or isotype control IgG (clone 2A3) (BioXcell) one day prior to infection and repeated daily. IL-1β was depleted by daily intraperitoneal injections of 100µg anti-mouse IL-1β antibody (clone 1400.24.17, Thermo Scientific) starting 30 min prior to infection.

Analysis of cytokine production in cell culture. 1×10^5 human or mouse neutrophils were plated in 96-well plates and stimulated with *C. albicans*. After 18 hrs supernatant or total cell lysate, obtained by adding 0.1% Nonidet P-40 was collected and cleared by centrifugation at 2000 rpm for 10 minutes. IL-1β content was assessed using mouse and human IL-1β ELISA Ready-SET-Go Kits (eBioscience). **In vivo.** Bronchoalveolar lavage was collected 24 and 48 hrs after infection with 1×10^5 *C. albicans* in 500µl sterile PBS, cleared by centrifugation at 2000 rpm for 10 minutes and stored at -80°C. Cytokine and chemokine protein expression was assayed using the following kits: mouse IL-1β ELISA Ready-SET-Go (eBioscience), mouse IL-6 ELISA (Invitrogen), mouse CXCL1/KC and CXCL2/MIP-2 Quantikine ELSIA kits (R&D) as well as IL-17 Quantikine ELISA kit (R&D) according to the manufacturer's instructions.

Immunoblot analysis. 1×10^6 human peripheral neutrophils were plated in 6-well plates and stimulated with *C. albicans*. At appropriate time points, cells were lysed in SDS sample buffer and separated by SDS-PAGE and western-blotted with anti-human MPO (Dako), anti-human p50 (Millipore), anti-human caspase-1 and IL-1β (R&D) or anti-actin (clone C4, Chemicon) antibodies followed by secondary rabbit anti-mouse IgG or goat anti-rabbit IgG antibodies coupled to HRP (horseradish peroxidase; Thermo Scientific). Oxidized proteins were detected with an OxyBlot™ kit (Chemicon). Samples were lysed in ice-cold

radioimmunoprecipitation assay (RIPA) buffer (50mM Tris-HCl, 150mM NaCl, 1% Nonidet P-40, 0.5% sodium deoxycholate (NaDoc), 0.1% sodium dodecyl sulfate (SDS), 1x Complete (Roche), 1µM Epoxomicin (Calbiochem), 10mM N-ethylmaleimide) and centrifuged at 14000 rpm for 15 min to separate cell debris. Protein concentration was determined using Micro BCA protein assay kit (Pierce) and lysate containing 5µg total protein was employed per reaction.

Immunoprecipitation. 7×10^6 human neutrophils were plated in 10cm cell culture dishes, pre-incubated with 200nM Epoxomicin (Calbiochem) for 1 hr to block degradation of oxidized proteins and stimulated for 1 hr with *C. albicans*. Cells were lysed in RIPA buffer and 150µg whole cell lysate was adjusted to 1ml volume in 20mM Tris-HCl, 10mM ethylenediaminetetraacetic acid (EDTA, Sigma-Aldrich), 100mM NaCl, 1% Nonidet P-40, 1x Complete, 10mM N-ethylmaleimide, 1µM Epoxomicin and 1mg/ml bovine serum albumin. 5µg anti-human p50 (Millipore) or anti-ubiquitin (Thermo Scientific, PA1-187) antibody was added per sample and precipitated over G sepharose 4FF, washed 3X with 0.1% Nonidet P-40/PBS and MilliQ water. For analysis of oxidation beads were taken up in 15µl RIPA buffer and subjected to a modified 2,4-Dinitrophenylhydrazine (DNPH)-derivatisation reaction of the OxyBlot™ protein oxidation detection kit (Chemicon): 6.4µl 20% SDS and 21µl 1x DNPH were added per sample for 15 min at RT. The reaction was terminated with 12.7µl neutralization solution and 5.5µl 1mM Dithiothreitol (DTT). Immune complexes were eluted by incubation at 80°C for 10 min. For analysis of ubiquitination, beads were eluted by adding 2x SDS sample buffer and incubation at 96°C for 5 min. 30µl sample were separated by SDS-PAGE and immunoblotted with the OxyBlot™ protein oxidation detection kit or anti-p50 antibody, respectively.

Subcellular fractionation and p50 binding assay. 5×10^6 human neutrophils were seeded in 10cm cell culture dishes. After stimulation with *C. albicans* for 45 min cells were lysed in 500µl fractionation buffer per plate (250mM sucrose, 20mM 4-(2-hydroxyethyl)-1-piperazineethanesulfonic acid (HEPES), 10mM KCl, 1.5mM $MgCl_2$, 1mM ethylenediaminetetraacetic acid (EDTA), 1mM ethyleneglycoltetraacetic acid (EGTA), 1mM Dithiothreitol (DTT), 1x Complete

(Roche), 1x PhosSTOP (Roche), 1μM Epoxomicin, 10mM N-ethylmaleimide). Lysates were passed 10 times through a 25G needle, incubated for 20 min on ice and centrifuged 5 min at 3000rpm. Pelleted nuclei were washed in 500µl fractionation buffer and resuspended in 50µl RIPA buffer. Nuclear lysate containing 10µg protein was used in a Pierce™ NFκB p50 Transcription Factor Assay (Thermo Scientific).

Quantitative real-time RT-PCR. Total cellular RNA from 1×10^6 human neutrophils in 6-well plates stimulated with *C. albicans* for 1 hr or tissue was isolated using TriReagent-Chloroform extraction followed by isopropanol precipitation (all chemicals from Sigma-Aldrich). 2µg RNA were reverse transcribed to generate cDNA with the Transcriptor high fidelity cDNA synthesis kit from Roche using anchored-oligo$(dT)_{18}$ primer. Gene expression was measured in duplicates using TaqMan Universal PCR Master Mix with gene-specific primers and probes for human and mouse IL-1β, IL-1α and IL-6 as well as HPRT1 or S100A8 on a 7900HT Fast Real Time PCR System (all from Applied Biosystems). The cycling-threshold (CT) for each gene was measured and normalized to that of the housekeeping gene HPRT1 or S100A8, respectively. The relative gene expression was calculated by the change-in-cycling-threshold (ΔΔCT) method using RQ manager software version 1.4 (Applied Biosystems).

Confocal microscopy of fixed cells and tissue. 5×10^4 human peripheral neutrophils were plated on glass coverslips. 1hr after stimulation, cells were fixed for 20 min at 37°C with 2% paraformaldehyde and permeabilized with 0.5% Triton X-100. Cells or lung parafinized tissues fixed in 4% paraformaldehyde were blocked with 2% bovine serum albumin and 2% donkey serum in PBS and incubated with anti-human p47-phox and anti-human p67-phox antibodies (Santa Cruz), anti-human myeloperoxidase (BD Bioscience), anti-mouse myeloperoxidase (R&D) and anti-*C. albicans* (Acris) antibodies followed by Alexa Fluor 488–conjugated donkey anti-mouse, Alexa Fluor 568–conjugated donkey anti-rabbit or Alexa Fluor 568–conjugated donkey anti-goat (Invitrogen), as well as Alexa Fluor 647-conjugated anti-Ly6G (Biolegend) and DAPI (4′,6-diamidino-2-phenylindole dihydrochloride; Life Technologies) before

being mounted in ProLong Gold (Molecular Probes) and imaged by confocal microscopy. For analysis of 200µm thick lung sections, tissue was frozen in OCT compound (VWR), sectioned on a Leica CM3050 S Cryostat, collected in PBS and stained floating before being mounted on glass slides. Images were analyzed with ImageJ v2.0 software. For analysis of neutrophil clustering whole lungs were imaged on an Olympus Slidescanner VS-120 and analysed with OlyVia 2.6 software (Olympus). The lung coverage of clusters was calculated using ImageJ v2.0 software proportional to the area of the whole lung determined by DAPI staining.

ROS and hyphal growth by time-lapse microscopy. 3×10^5 human peripheral neutrophils were plated on glass-bottom Petri dishes (MatTEK) and incubated with heat-inactivated yeast or hyphae at an MOI of 30 and imaged by confocal microscopy every 30 sec at 37°C and 5% CO_2 in the presence of SYTOX (Invitrogen) for NET release or nitroblue tetrazolium chloride (NBT, Sigma-Aldrich) for detection of ROS. Hyphal growth inhibition and the number of associated neutrophils and their NET release were measured by imaging live preformed hyphae and neutrophils every 2 min as above by epifluorescence time-lapse video microscopy. Hyphal length was measured between 2 and 12 hrs of incubation using ImageJ software and correlated to the number of associated neutrophils at 2 hrs.

Flow cytometry. Lungs were harvested, finely minced and digested with 0.4 mg/ml Liberase TL (Roche) for 45 min at 37°C. Subsequently, tissue was meshed through cell strainers to obtain single cell suspensions and erythrocytes were lysed by incubation in ammonium-chloride-potassium (ACK)-lysis buffer for 5 min at room temperature. For intracellular cytokine staining Golgi Stop (BD Bioscience) was added during the isolation of single cells followed by 2 hrs incubation at 37°C. 2×10^6 cells were used for FACS staining with the following antibodies: mouse CD45-APC-Cy7, CD11b-APC (both BD Bioscience), Ly6G-FITC and Ly6C-PE (both purchased from BioLegend) and IL-1β-APC (R&D), human CD14-PE, CD15-Pacific blue and CD66b-FITC (all BioLegend). Dead cells were excluded with the Live/Dead Fixable Aqua Dead Cell Stain Kit purchased from

Life Technologies. Multi-colour cytometry was acquired on BD LSR II Flow Cytometer (BD Bioscience) and analysed with FlowJo v10 (Tree Star) software.

ROS measurements. 1×10^6 human neutrophils were plated in white Nunclon 96-well plates. After 1 hr preincubation at 37°C cells were stimulated with *C. albicans* yeast or preformed hyphae at MOI 2.5. Total ROS production was measured by adding cell-permeable luminol (100µM), extracellular ROS were assessed by adding cell-impermeable isoluminol (100µM) both in the presence of 1.2 U/ml horseradish peroxidase (all chemicals Sigma-Aldrich). Chemiluminescence was measured immediately every 30 seconds over 2 hrs on a FLUOstar Omega plate reader (BMG Labtech).

XTT viability assay. Neutrophils (5×10^3 – 5×10^5) were seeded in 200µl medium in 96-well plates. Subsequently, neutrophils were stimulated with 5×10^4 *C. albicans* preformed hyphae (MOI 10 to 0.1 respectively). At indicated time points 50µl of 6mg/ml 2,3-Bis-(2-Methoxy-4-Nitro-5-Sulfophenyl)-2H-Tetrazolium-5-Carboxanilide (XTT) was added. The plate was incubated for 30 min at 37°C and 100µl of the reaction were transferred into a new 96-well plate and absorbance was read at 450nm. Viability was expressed as fold change compared to *C. albicans* hyphae alone.

Statistical analysis. Statistical significance was assessed by an unpaired, two-tailed Student's *t*-test for single comparison and a two-way analysis of variance (ANOVA) followed by Sidak's multiple comparison post test for multiple comparisons. *P* values of less than 0.05 were considered significant.
